# Exposure of the lungs in breast cancer radiotherapy: A systematic review of lung doses published 2010–2015

**DOI:** 10.1016/j.radonc.2017.11.022

**Published:** 2018-01

**Authors:** Marianne C. Aznar, Frances K. Duane, Sarah C. Darby, Zhe Wang, Carolyn W. Taylor

**Affiliations:** a Clinical Trial Service Unit, Nuffield Department of Population Health, University of Oxford, UK; b Department of Oncology, Rigshospitalet, Copenhagen University Hospital, Denmark; c Medical Research Council Population Health Research Unit, Nuffield Department of Population Health, University of Oxford, UK

**Keywords:** Breast cancer, Lung dose, Imrt, Breathing adaptation

## Abstract

**Background and purpose:**

We report a systematic review of lung radiation doses from breast cancer radiotherapy.

**Methods and materials:**

Studies describing breast cancer radiotherapy regimens published during 2010–2015 and reporting lung dose were included. Doses were compared between different countries, anatomical regions irradiated, techniques and use of breathing adaptation.

**Results:**

471 regimens from 32 countries were identified. The average mean ipsilateral lung dose (MLD_ipsi_) was 9.0 Gy. MLD_ipsi_ for supine radiotherapy with no breathing adaption was 8.4 Gy for whole breast/chest wall (WB/CW) radiotherapy, 11.2 Gy when the axilla/supraclavicular fossa was irradiated, and 14.0 Gy with the addition of internal mammary chain irradiation; breathing adaptation reduced MLD_ipsi_ by 1 Gy, 2 Gy and 3 Gy respectively (*p* < 0.005). For WB/CW radiotherapy, MLD_ipsi_ was lowest for tangents in prone (1.2 Gy) or lateral decubitus (0.8 Gy) positions. The highest MLD_ipsi_ was for IMRT in supine position (9.4 Gy).

The average mean contralateral lung dose (MLD_cont_) for WB/CW radiotherapy was higher for IMRT (3.0 Gy) than for tangents (0.8 Gy).

**Conclusions:**

Lung doses from breast cancer radiotherapy varied substantially worldwide, even between studies describing similar regimens. Lymph node inclusion and IMRT use increased exposure, while breathing adaptation and prone/lateral decubitus positioning reduced it.

Radiotherapy improves survival in several categories of women with early breast cancer [1], [2]. When irradiating the breast or chest wall with or without the regional lymph nodes exposing the lungs is unavoidable and this incidental exposure may increase the risk of subsequent primary lung cancer [3], [4], [5], [6], pneumonitis [7] and lung fibrosis [8]. The risk of these side-effects increases with lung radiation exposure, so knowledge of the doses received by the lungs with modern breast cancer radiotherapy is important. This topic is particularly relevant today, as three large studies recently demonstrated that in women with high risk breast cancer, radiotherapy to the regional lymph nodes including the internal mammary chain (IMC) significantly reduces breast cancer recurrence [9], [10], [11]. These nodes are close to the lungs, so irradiating them can increase lung radiation exposure.

For lung cancer, radiation approximately multiplies a woman’s pre-existing risk, so the absolute risk of primary lung cancer from radiotherapy will be much higher for a smoker than for a non-smoker. In an individual patient data meta-analysis of 75 randomised trials of breast cancer radiotherapy, the risk of radiation-related lung cancer increased by ∼11 per cent (95% confidence interval 6–19) per Gy mean lung dose [6]. The estimated absolute risk of radiation-related lung cancer from 5 Gy mean whole lung dose was ∼4% for a smoker but <1% for a non-smoker. Hence particular attention needs to be paid to minimising lung doses in smokers [6], [12].

Pneumonitis is another possible complication of breast cancer radiotherapy, occurring within three months of radiotherapy [7] and leading to lung fibrosis several months later [8]. As with radiation-related lung cancer, the risk of pneumonitis increases with increasing lung radiation dose [7], [13]. It is also more common in women who receive chemotherapy in addition to radiotherapy [7], [14], [15], [16].

In order to assist with weighing the benefit against the risks of different breast cancer radiotherapy regimens, we present a systematic review of lung doses from breast cancer radiotherapy dosimetry studies published during 2010–2015. We describe how lung exposure varies according to country, anatomical regions irradiated, technique, treatment position and the use of breathing adaptation. In addition, we discuss the likely future absolute risks of radiation-related lung cancer for women irradiated recently.

## Methods

### Study identification

Studies were identified following the Preferred Reporting Items for Systematic Reviews and Meta-Analyses (PRISMA) guidelines [17]. Embase and Scopus were queried using search terms (“dos∗ AND breast∗ AND cancer∗/carcinom∗/tumor∗/tumour∗ AND radiation/radiotherapy∗”) to retrieve breast cancer radiotherapy dosimetry studies published between 1 January 2010 and 18 September 2015. All studies reporting any measure of lung dose were eligible, regardless of whether the plans were actually delivered. Reference sections of eligible papers were scanned to identify additional eligible studies. Studies reporting only dose from a tumour bed boost were excluded because this is nearly always given alongside whole breast radiotherapy, which delivers higher lung doses. Studies of bilateral breast cancer were also excluded.

### Data abstraction

Eligible studies were categorised according to radiotherapy technique (Table E1-E2). The information abstracted from each study included: author, year, country of first author, patient position, radiotherapy planning technique (e.g. conformal vs IMRT), field type (e.g. static vs rotational), beam energy, breathing adaptation, whether or not the radiotherapy plans were delivered, region(s) irradiated, prescription dose to the target and number of fractions, number of CT planning scans per regimen included in the study, the type of dose calculation algorithm, the presence of unfavourable anatomy and cancer laterality. Lung dose measures abstracted were: mean ipsilateral lung dose (MLD_ipsi_), mean contralateral lung dose (MLD_cont_), mean whole lung dose (i.e. where both lungs were considered a single organ) (MLD_whole_), V5_ipsi_ (i.e. percent ipsilateral lung volume irradiated to 5 Gy or more), V10_ipsi_, V20_ipsi_, V30_ipsi_ and V40_ipsi_. Dose calculation algorithms [18] were categorised as “type A” (no/poor modelling of lateral electron transport), “type B” (some modelling of lateral electron transport), Monte Carlo and “other”. Each regimen was classified as to whether or not it was reported for a high income country [19].

### Data analyses

Analyses considered the average of the lung dose measures from the CT plans included for each regimen described in each study. We term these “average lung doses”. Average lung doses were compared between countries, region(s) irradiated, techniques used, patient treatment positions and whether breathing adaption was used. Variation in the average doses within each of these categories was assessed using chi-squared tests for heterogeneity, difference or trend, as appropriate.

## Results

**Fig. 1:**
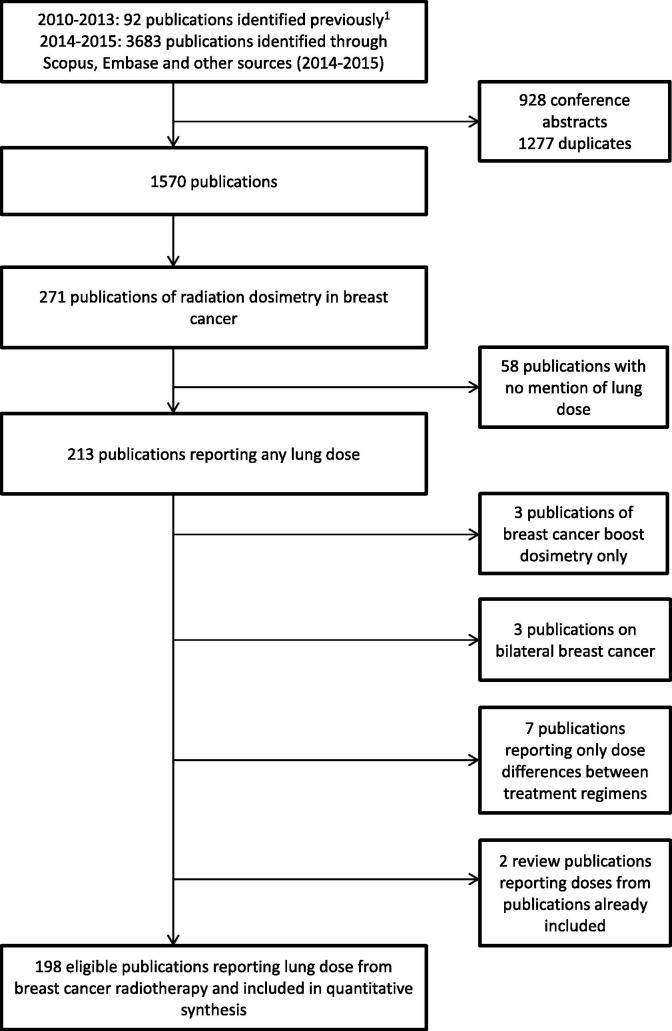
The process of study identification for the review.

Radiation doses to the lungs from breast cancer radiotherapy were reported in 198 studies from 32 countries including 579 regimens (Fig. 1, Table 1, Tables E3,E4). The year in which the CT plans were generated was reported in 64/198 studies: it was before 2005 in four studies, 2005–09 in 29 studies and 2010–15 in 31 studies. 3D–treatment planning was used in all 198 studies; one study simulated a 2D regimen on 3D CT scans with field borders based on bony structures and midline wire marking [20]. MLD_ipsi_ was available for 471 regimens in 153 studies and was the commonest dose measure reported (Table 1). Among those 153 studies, 94 reported MLD_ipsi_ separately for left-sided and right-sided breast cancer radiotherapy regimens. The difference in average MLD_ipsi_ according to laterality was small and not significant (left-sided 9.5 Gy in 287 regimens, right-sided 8.7 Gy in 38 regimens, *p* for difference = 0.94). Therefore, left-sided and right-sided regimens were considered together in our analyses.

**Table 1:** Studies reporting lung doses from breast cancer radiation therapy regimens and published during 2010–2015.

Breast cancer laterality	Dose measure	Number of Studies*	Number of regimens†	CT plans per regimen‡		Lung dose	
				Average	Range	Average	Range
Left	MLD_ipsi_	90	287	16	1–148	9.5 Gy	0.3–27.5§
	MLD_cont_	39	147	11	1–31	2.5 Gy	0–15.0§
	V20_ipsi_	81	258	17	1–148	16.8%	0–44.5
	V5_ipsi_	52	162	18	1–148	41.8%	0.8–94
Right	MLD_ipsi_	16	38	7	1–45	8.7 Gy	1.2–16.1
	MLD_cont_	6	13	3	1–14	2.7 Gy	0.2–6.5
	V20_ipsi_	12	25	11	1–45	16.1%	4.7–35.6
	V5_ipsi_	6	14	6	1–47	42.4%	1.5–88.7
Unspecified	MLD_ipsi_	60	146	33	6–494	7.9 Gy	0.3–22.9
	MLD_cont_	21	58	24	6–246	1.6 Gy	0–6.4
	V20_ipsi_	58	135	40	6–494	13.9%	0.1–44.5
	V5_ipsi_	27	57	34	8–431	32.2%	0.7–99.3
All studies reporting MLD_ipsi_		153	471	21	1–494	9.0 Gy	0.3–27.5‡
All studies reporting MLD_cont_		62	218	14	1–246	2.3 Gy	0–15.0‡
All studies reporting V20_ipsi_		139	417	19	6–494	15.8%	0–44.5
All studies reporting V5_ipsi_		78	233	21	1–431	39.5%	0.7–99.3
All studies		198|	579	–	–	–	–

Definitions: MLD_ipsi_: mean dose to the ipsilateral lung, MLD_cont_: mean dose to the contralateral lung, V20_ipsi_: percent volume of the ipsilateral lung receiving 20 Gy or more, V5_ipsi_: percent volume of the ipsilateral lung receiving 5 Gy or more.

* Some studies reported doses for both left-sided and right-sided regimens and so contribute more than once.

† Some regimens reported several dose measures (e.g. both MLD_ipsi_ and V20_ipsi_).

‡ For four regimens in one study the number of CT planning scans was not reported.

§ Four regimens (three from Al-Rabhi 2013, one from Ares 2010, see full references in Table E4) were excluded because doses reported were inconsistent with values presented elsewhere in the publication.

| This total represents the number of unique studies, without the multiple contributions from studies which reported doses for both left-sided and right-sided regimens.

### Tangential regimens

**Fig. 2:**
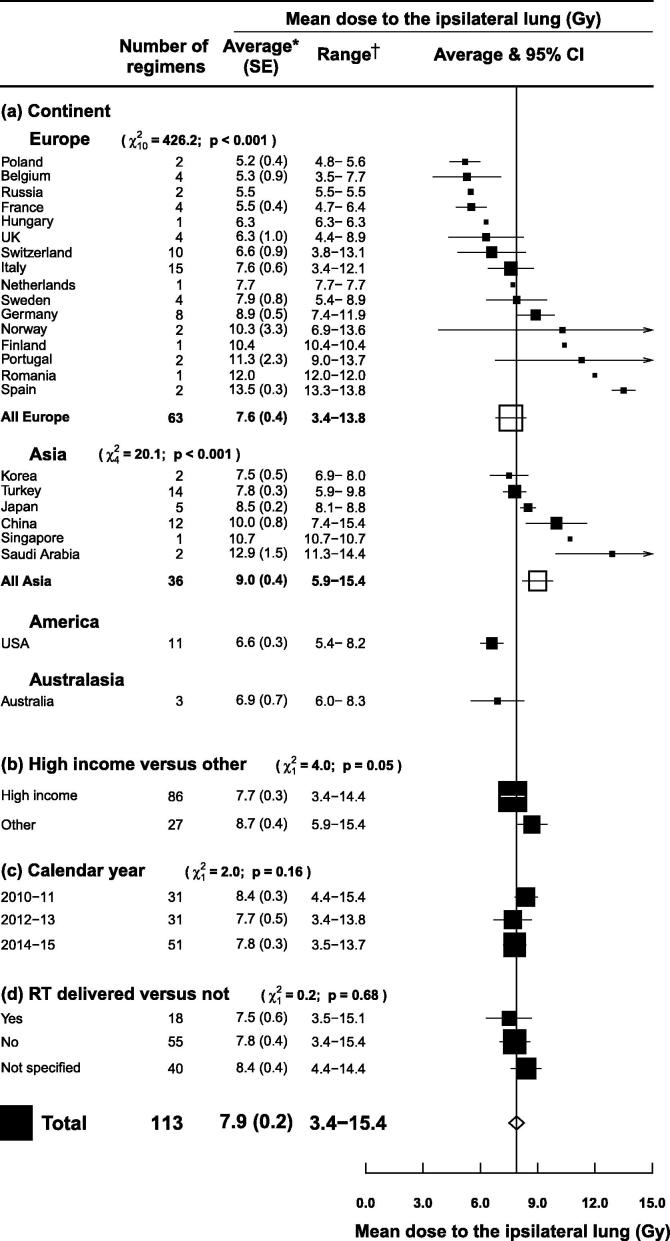
Mean ipsilateral lung dose (MLD_ipsi_) from left or right tangential breast cancer radiotherapy according to country, gross national income per person in the country concerned, calendar year, and whether the radiotherapy plans were actually delivered or just planned. Regimens that irradiated the internal mammary chain, partial breast, axilla, or supraclavicular fossa were excluded, as were regimens with breathing adaptation, prone or lateral decubitus positioning and studies of women with unfavourable anatomy. *Average of mean ipsilateral lung doses for reported regimens. †Range of mean ipsilateral lung doses for reported regimens. *χ*^2^ and *p* values are for: heterogeneity (a), difference (b, d) or trend (c). For (d), the category “Not specified” was omitted from the *χ*^2^. *Abbreviations:* SE: standard error; CI: confidence interval.

Whole breast/chest wall tangential radiotherapy in supine position with no breathing adaption was the most commonly reported combination of technique and regions irradiated (113 regimens in 70 studies) (Fig. 2). The average MLD_ipsi_ for all 113 regimens was 7.9 Gy (SE 0.2) and it varied significantly according to continent (*p* for heterogeneity <0.001). There was also substantial variation according to country within Europe and Asia (*p* for heterogeneity <0.001 for both). The average MLD_ipsi_ was lowest for Poland and Korea (5.2 and 7.5 Gy respectively) and highest for Spain and Saudi Arabia (13.5 and 12.9 Gy respectively). The average MLD_ipsi_ in “high income” countries was lower than in other countries (7.7 versus 8.7 Gy, *p* for difference = 0.05), but it did not vary significantly according to calendar year of publication (*p* for trend = 0.16) or whether the treatment plans were actually delivered (*p* for difference = 0.68).

### Regions irradiated

**Fig. 3:**
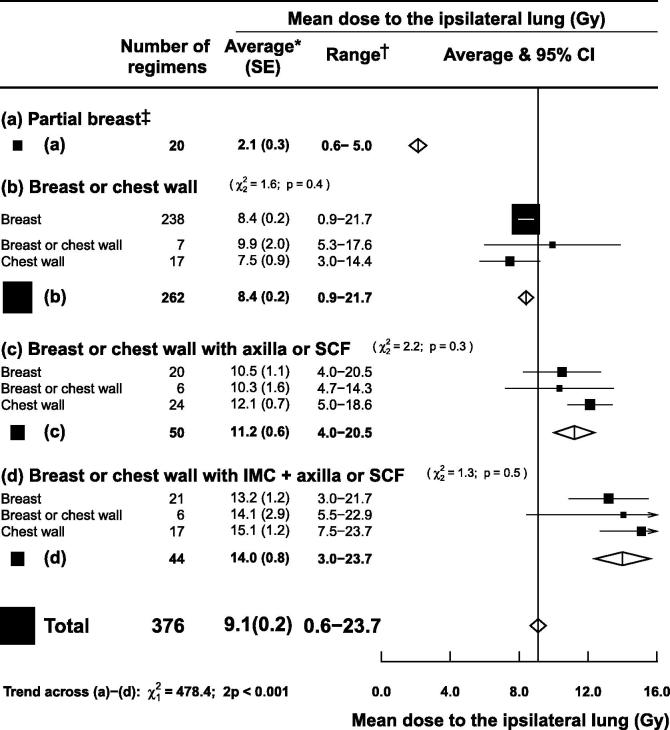
Mean ipsilateral lung dose (MLD_ipsi_) from left or right breast cancer radiotherapy according to regions irradiated. Studies of women with unfavourable anatomy were excluded as were regimens using prone or lateral decubitus positioning, and regimens irradiating the internal mammary chain (IMC) but not the axilla or the supraclavicular fossa (SCF). *Average of mean ipsilateral lung doses for reported regimens. †Range of mean ipsilateral lung doses for reported regimens. *χ*^2^ and *p* values are for heterogeneity. *Abbreviations:* SE: standard error; CI: confidence interval.

After excluding studies of women with unfavourable anatomy and regimens in the prone or lateral decubitus positions, 376 of the 471 regimens remained, with an average MLD_ipsi_ of 9.1 Gy (SE 0.2). The average MLD_ipsi_ was highly correlated with the extent of the regions irradiated (2p for trend <0.001, Fig. 3). For partial breast irradiation (20 regimens) average MLD_ipsi_ was 2.1 Gy (SE 0.3) and for whole breast/chest wall irradiation (262 regimens) it was 8.4 Gy (SE 0.2). The addition of axilla/supraclavicular fossa irradiation (50 regimens) increased it to 11.2 Gy (SE 0.6) and inclusion of the internal mammary chain (IMC) (44 regimens) further increased it to 14.0 Gy (SE 0.8).

### Technique and treatment position

**Fig. 4:**
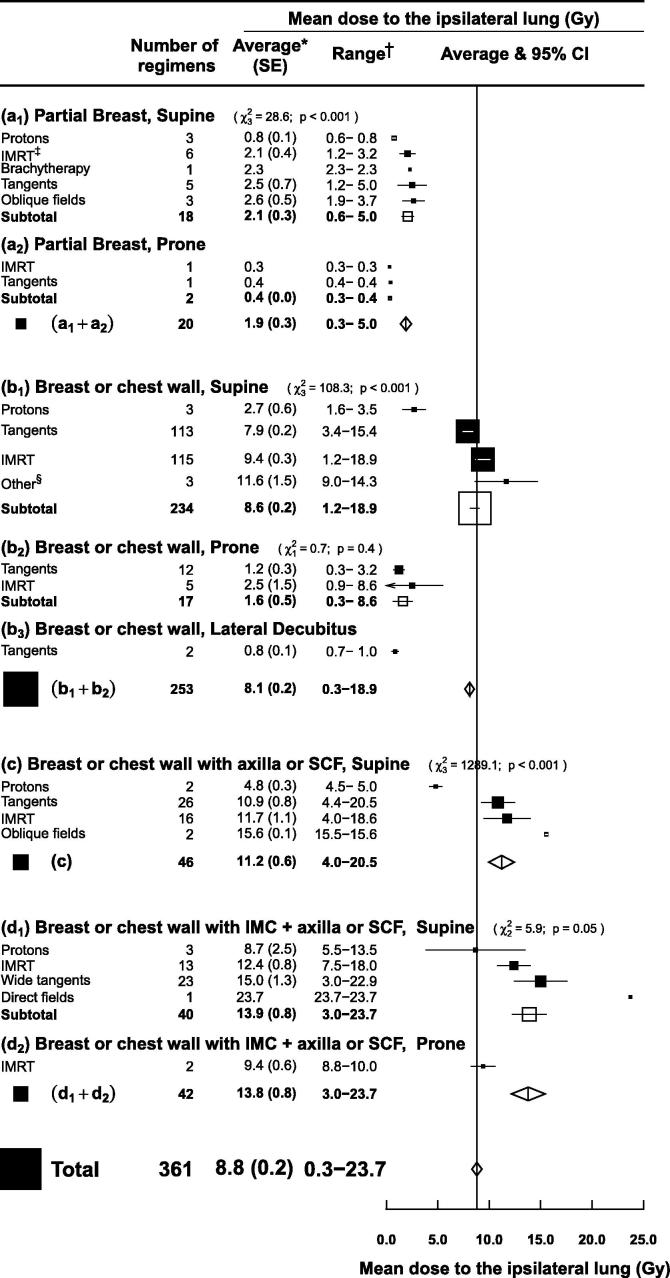
Mean ipsilateral lung dose (MLD_ipsi_) from left or right breast cancer radiotherapy according to regions irradiated and technique used. Regimens using breathing adaptation (e.g. deep inspiration breath hold) were excluded, as were studies of women with unfavourable anatomy and regimens irradiating the internal mammary chain (IMC) but not the axilla or the supraclavicular fossa (SCF). *Average of mean ipsilateral lung doses for reported regimens. †Range of mean ipsilateral lung doses for reported regimens. ‡Static field IMRT and rotational IMRT are included jointly as “IMRT”. § “Other” techniques included two dynamic conformal arc therapy regimens and one unspecified 3D conformal regimen. *χ*^2^ and *p* values are for heterogeneity. *Abbreviations*: SE: standard error; CI: confidence interval; IMRT: intensity modulated radiotherapy. IMC: internal mammary chain. SCF: supraclavicular fossa.

For each combination of region(s) irradiated, the average MLD_ipsi_ varied according to both technique and treatment position (Fig. 4). The commonest treatment position was supine (338 regimens). The commonest techniques were tangents (159 regimens) and intensity modulated radiotherapy (IMRT) (156 regimens). Average MLD_ipsi_ was similar for static field and rotational IMRT so these two types of IMRT were considered together.

For 20 partial breast regimens (Fig. 4, panels a_1_ and a_2_), the average MLD_ipsi_ for the different techniques and treatment positions varied from 0.3 to 2.6 Gy. For 234 whole breast/chest wall regimens in supine position (Fig. 4, panel b_1_) the MLD_ipsi_ was 7.9 (SE 0.2) from tangents and 9.4 (SE 0.3) from IMRT (*p* for difference <0.001). Doses for these techniques from 17 whole breast/chest wall regimens in prone position were lower: MLD_ipsi_ 1.6 (SE 0.5). The lowest doses were from two whole breast/chest wall regimens in lateral decubitus position: MLD_ipsi_ = 0.8 (SE 0.1).

When the axilla/SCF was also irradiated (Fig. 4, panel c), the commonest regimens were tangents (26 regimens) which delivered MLD_ipsi_ = 10.9 (SE 0.8) and IMRT (16 regimens), which delivered MLD_ipsi_ = 11.7 (SE 1.1). When the IMC was also included (Fig. 4, panels d_1_ and d_2_), there were 23 wide tangential regimens which delivered MLD_ipsi_ = 15.0 (SE 1.3) and 13 IMRT regimens, which delivered MLD_ipsi_ = 12.4 (SE 0.8). The highest reported MLD_ipsi_ values were for rare techniques that included oblique or direct fields and were not actually delivered to patients.

Doses from proton therapy were reported for 11 regimens. Within each category of regions irradiated, proton therapy resulted in the lowest average MLD_ipsi_: partial breast 0.8 Gy (SE 0.07), whole breast/chest wall 2.7 Gy (SE 0.6), whole breast/chest wall and axilla/supraclavicular fossa 4.8 Gy (SE 0.3), whole breast/chest wall and axilla/supraclavicular fossa with IMC 8.7 Gy (SE 2.5) (Fig. 4, panels a_1_, b_1_, c_1_ and d_1_).

### Breathing adaptation

**Fig. 5:**
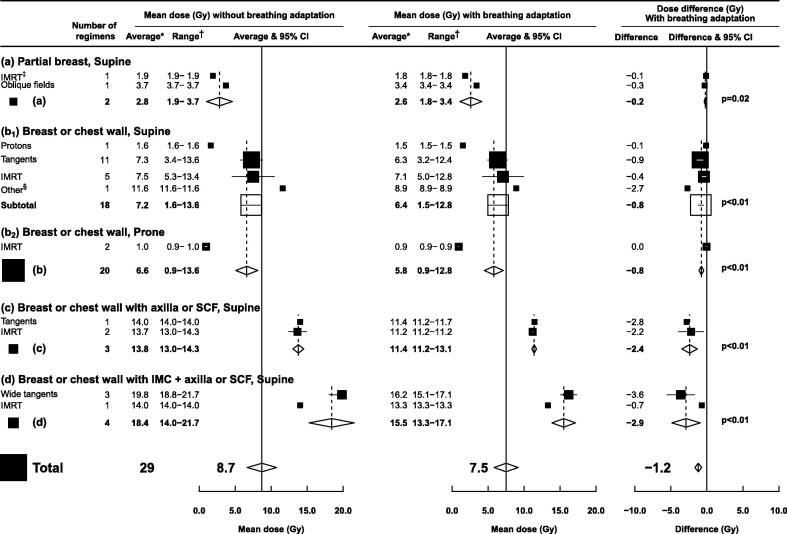
Mean ipsilateral lung doses (MLD_ipsi_) from left or right breast cancer radiotherapy with or without breathing adaptation according to regions irradiated and regimens used. Only studies providing doses with and without breathing adaptation in the same woman were included. Studies of women with unfavourable anatomy were excluded. *Average of mean ipsilateral lung doses for reported regimens. †Range of mean ipsilateral lung doses for reported regimens. ‡Static field IMRT and rotational IMRT are included jointly as “IMRT”. § “Other” includes one unspecified 3D conformal regimen. *p* values are calculated using a paired *t*-test. *Abbreviations:* CI: confidence interval; IMRT: intensity modulated radiotherapy; IMC: internal mammary chain; SCF: supraclavicular fossa.

For 29 regimens (21 studies) the average MLD_ipsi_ was reported for a given regimen both with and without breathing adaption in the same women (Fig. 5). Breathing adaptation reduced the average MLD_ipsi_ significantly in all regimens, with the magnitude of the reduction depending on the extent of the regions irradiated. For partial breast irradiation the average reduction was 0.2 Gy; for whole breast/chest wall radiotherapy it was 0.8 Gy; for regimens that included the axilla/supraclavicular fossa it was 2.5 Gy; and for regimens that also included the IMC it was 2.9 Gy. These reductions were seen regardless of radiotherapy technique or patient positioning.

### Other measures of lung dose

The average V20_ipsi_ (311 regimens, 139 studies) was 15.9% and the average V5_ipsi_ (174 regimens, 77 studies) was 40.9% (Fig. E1, E2). For treatment in the supine position not including the IMC, there were no material differences in V20_ipsi_ between IMRT and tangents (Fig. E1 panels a, b_1,_ c). When the IMC was included, IMRT led to a substantially lower V20_ipsi_ than wide tangents (*p* < 0.001) (Fig. E1 panel d_1_). Prone positioning was effective at reducing V20_ipsi_, and for each region category for which it was reported average V20_ipsi_ for protons was lower than for other techniques. V5_ipsi_ was higher for IMRT than for tangential regimens regardless of the regions irradiated (Fig. E2). Other volumetric dose measures such as V30_ipsi_ and V40_ipsi_ were each reported in fewer than 50 studies.

The average MLD_cont_ (168 regimens, 62 studies) was 2.2 Gy (Fig. E3). MLD_cont_ was higher for IMRT than for tangential regimens, regardless of the regions irradiated. For partial breast radiotherapy, the average MLD_cont_ was 0.2 Gy. For the other region categories, average MLD_cont_ varied from 1.9 to 2.6 Gy, and did not increase according to the extent of the ipsilateral regions irradiated. The lowest contralateral lung doses within each region category were from proton therapy.

The average MLD_whole_ (218 regimens, 88 studies) was 6.0 Gy (Fig. E4). Variation according to regions irradiated, technique, and patient position was similar to MLD_ipsi_ (Fig. 4).

### Dose calculation algorithm

The dose calculation algorithm used for external beam photon radiotherapy was reported in 147/198 (74%) of the studies. Of these, 108 studies used a type B algorithm, 30 studies used type A and 9 studies used Monte Carlo. No significant differences in MLD_ipsi_, V20_ipsi_, V5_ipsi_, MLD_cont_ or MLD_whole_ were observed according to algorithm (*p* for heterogeneity >0.8 for all dose measures) (data not shown).

## Discussion

This systematic review of lung doses from breast cancer radiotherapy regimens published during 2010–2015 shows that lung exposure varied substantially worldwide but that several factors influenced it systematically. Lung doses increased with more extensive regions irradiated and reduced with the use of breathing adaptation or prone or lateral decubitus positioning. Lung doses also depended on the technique used, with the highest doses from IMRT and lowest doses from proton therapy.

### Methods to reduce lung exposure

There are several ways of reducing lung dose in breast cancer radiotherapy today. First, in our study, limiting the extent of irradiated regions reduced MLD_ipsi_ (Fig. 3). This is often done in current practice, for example regional nodal irradiation is only usually recommended in women at high risk of cancer recurrence, while in some women at very low risk of recurrence, radiotherapy may be omitted altogether [21]. Second, lung-sparing techniques may be used. The lowest ipsilateral lung doses for breast/chest wall irradiation were from proton therapy (<2.7 Gy) or from prone or lateral decubitus positioning (<2.5 Gy) (Fig. 4). These techniques also reduce mean heart doses [22] but are not yet widely used: proton therapy is expensive and available in only a few centres [23], while among the papers included in this review only one reported lung doses for lateral decubitus positioning and 15 reported doses for prone positioning. The limited implementation of new positioning techniques is due to the need for specialised equipment and concerns about the reproducibility of daily patient set-up [24]. For regimens that did not include the IMC, tangents spared the lungs better than IMRT (Fig. 4). When the IMC was irradiated, IMRT resulted in lower mean ipsilateral lung dose than tangents (Fig. 4, Fig. E1) but it increased contralateral lung dose (Fig. E2, Fig. E3). In clinical practice, contralateral lung dose may be reduced by limiting the number of beams – or using a partial arc in rotational treatments – to avoid beam entry through the contralateral lung. In addition, strict optimisation constraints and strategic beam placement may minimise low doses in the ipsilateral lung. Finally, lung dose can be reduced using breathing adaptation [25]. Historically this has been used to reduce cardiac exposure [24] but our study suggests that it also reduces lung exposure, with the magnitude of the gain increasing with more extensive regions irradiated (Fig. 5).

### Strengths and limitations

Our study has several strengths. First, this comprehensive systematic review included 471 regimens and 153 studies, with the main analyses based on the most commonly reported measure of lung exposure, MLD_ipsi_, Second, our doses are likely to represent normal radiotherapy practice, since most included studies focussed on target volume coverage or heart dose and lung doses were reported only for completeness rather than as a primary endpoint. As such, it is unlikely that the groups who published these doses paid more attention to minimising lung exposure than other groups who did not publish their lung doses. Third, this systematic review was carried out by both a radiation oncologist and a physicist to ensure that all regimens were reliably categorised.

Our study also has some limitations. For a given regimen, there was variation in the lung doses received by individual patients. Kim et al [26] reported lung doses for a tangential field-in-field regimen delivered to 157 women and found that the MLD_ipsi_ for individual women ranged from 2.8-18.7 Gy. Therefore, although our study provides a summary of the average doses from various regimens, lung doses also vary substantially from patient to patient. In addition, lung doses were presented separately for women with left-sided and right-sided breast cancer in only 38 regimens (16 studies), which limited our investigation of the potential differences between left- and right-sided regimens.

### Absolute benefits and risks

Our results may be helpful in weighing the benefit against the risks of different breast cancer regimens. Large meta-analyses have shown that in most women irradiated for breast cancer according to current guidelines, the absolute benefits of radiotherapy outweigh the risks [1], [2], [26]. The risk of lung cancer increases with lung dose (i.e. MLD_whole_). In our study, the typical MLD_whole_ from modern radiotherapy was around 5 Gy from whole breast or chest wall regimens (Fig E4). Estimates of absolute risk based on data from 40,000 women in 75 randomised trials suggest the absolute 30-year risk of radiation-related lung cancer from MLD_whole_ 5 Gy for a typical non-smoker aged 50 years at irradiation would be only ∼0.3%. For a long-term continuing smoker it is considerably higher, at ∼4% [6], although this would be reduced if the woman stopped smoking [12], [27]. In our study, regimens that irradiated the axilla/supraclavicular fossa and IMC increased the MLD_whole_ to around 9 Gy, which would increase the typical absolute 30-year risk of radiation-related lung cancer to ∼0.6% for a non-smoker and ∼10% for a long-term continuing smoker. Hence, for some long-term smokers, the risks of irradiating the SCF, axilla and IMC may outweigh the benefits, especially if they continue to smoke [6]. In applying these estimated risks, it should be remembered that they are for typical modern radiotherapy in typical populations of women. But, as we have shown, lung doses in breast cancer regimens vary from patient to patient, as do population disease-rates, so the absolute risks, and the effect of smoking cessation for individual women will vary around these typical values.

## Conclusions

Lung exposure in breast cancer radiotherapy varied substantially between different countries and regimens, so the radiation-related risks of lung cancer, pneumonitis and lung fibrosis will also vary. Exposure may be reduced by minimising the extent of the irradiated region, using breathing adaptation and using prone or lateral decubitus patient positioning or proton therapy. These lung doses may help oncologists tailor treatment for women who have high estimated risks of radiation-related lung cancer and fibrosis.
