# Supplementary material for: Exposure of the lungs in breast cancer radiotherapy: A systematic review of lung doses published 2010–2015
Source: Radiother Oncol. 2018 Jan;126(1):148–54. doi: 10.1016/j.radonc.2017.11.022 (PMC5807032; doi:10.1016/j.radonc.2017.11.022)
Supplement: Supplementary data 1 [file mmc1.pdf]

Table E1. Definitions of radiotherapy planning and delivery used in review

Category	Sub-category	Definition
Patient position	Supine	Patient lying on back
	Prone	Patient lying on front
	Lateral decubitus	Patient lying on side
Treatment planning	2D Conformal	Single slice patient contour information with shaped fields (usually simple block)
	3D Conformal	Multi-CT patient contour slices used, with shaped fields
	3D Conformal with FIF	Multi-CT patient contour slices used, with shaped fields and multiple segments (field in field)
	3D IMRT	Multi-CT patient contour slices used, with inverse-planned IMRT
	Other	Other type of treatment planning
Field type	Tangents	Fixed gantry angles, tangential fields with medial border on midline or to the ipsilateral side
	Tangents plus electron field	Fixed gantry angles, tangential fields with medial border on midline or to the ipsilateral side, plus a single electron field
	Tangents plus boost/direct fields	Fixed gantry angles, tangential fields with medial border on midline or to the ipsilateral side, plus one or more direct photon field(s)
	Wide tangents	Fixed gantry angles, tangential fields with medial border to the contralateral side
	Wide tangents plus boost/direct fields	Fixed gantry angles, tangential fields, with medial border to the contralateral side plus one or more direct photon fields
	Wide tangents plus electron field	Fixed gantry angles, tangential fields with medial border to the contralateral side plus a single electron field
	Partially wide tangents	Fixed gantry angles, tangential fields, with part of medial border to the contralateral side
	Rotational fields	Gantry rotating during treatment, either using tomotherapy or a linear accelerator (e.g. volumetric modulated arc therapy). Topotherapy was defined as static fields, even though the patient moves through the machine aperture during treatment
	Direct fields	Single or multiple direct fields (photon, electron or proton) “en face”
	Oblique fields	Single or multiple fields (photon, electron or proton), at an oblique angle to the skin
	Other	Other field type
Beam energy & Modality	Low energy MV photons	Single energy MV photons, with an energy between 4-8MV
	Mixed energy MV photons	Multiple energy MV photons e.g. 6 and 15MV in the same treatment plan
	High energy MV photons	Single energy MV photons, with an energy between 10-18MV
	MV photons	MV photon beams, with energy not specified
	Mixed photons & electrons	MV photon and MeV electron beams
	Electrons	MeV electron beams
	Protons	Proton beams
	Protons plus photons	Proton plus photon beams
	Brachytherapy	Kilovoltage energies from a brachytherapy source
	Carbon ions	Carbon ion beams
Breathing adaptation	No breathing adaptation	No breathing adaptation used at either planning or treatment stages
	Breathing adaptation	Breathing adaptation used at either planning or treatment stages (multiple methods were used e.g. deep inspiratory breath hold, gating, active breathing control)
RT plan delivered	Yes	The radiotherapy plans were delivered to the women
	No	The radiotherapy plans were not delivered
	Some	Some of the radiotherapy plans were delivered

Category	Sub-category	Definition
Calculation algorithm	Type A	No modelling of lateral electron transport, MV photons
	Type B	Lateral electron transport modelled, MV photons
	Brachytherapy	Brachytherapy treatment planning
	Protons	Proton treatment planning
	MC	Monte Carlo (MV photons)
	NS	Not specified

Abbreviations:
3D: 3-dimensional
2D: 2-dimensional
FIF: field-in-field
IMRT: intensity modulated radiation therapy
MeV: mega electron volt
MV: megavoltage

Table E2. Grouping of sub-categories used to create the types of "radiotherapy technique" shown in figures 3, 4 and 5:

Radiotherapy technique	Sub-categories included
Protons	Beam energy & modality: Protons, Protons plus photons
Brachytherapy	Beam energy & modality: Brachytherapy
Tangents	Treatment planning: 3D Conformal, 3D Conformal with FIF, 2D Conformal plan, NS<br>Field type: Tangents, Tangents plus electron fields, Tangents plus boost/direct field
Wide tangents	Treatment planning: 3D Conformal, 3D Conformal with FIF, 2D Conformal plan, NS<br>Field type: Wide tangents, Wide tangents plus boost/direct fields, Partially wide tangents
IMRT	Treatment planning: 3D IMRT<br>(includes static IMRT and rotational fields (e.g. volumetric modulated arc therapy))
Oblique fields	Treatment planning: 3D Conformal, 3D Conformal with FIF, 2D Conformal plan, NS<br>Beam energy & modality: photons, electrons, protons and combinations<br>Non tangential fields arrangements without inverse planning
Others	Anything not in the above categories

Abbreviations:
3D: 3-dimensional
2D: 2-dimensional
FIF: field-in-field
IMRT: intensity modulated radiation therapy

Table E3. Regimens and lung doses from breast cancer radiotherapy published in 2010-2015

Author	Year	Country	Description of regimens*									No. of CT plans	Lung dose measures					
			Patient position	Treatment planning	Field type	Beam energy & modality	Breath. adapt.	RT plan delivered	Target(s)	Prescr. dose	No. of fraction		MLD_{ipsi} (Gy)		MLD_{cont} (Gy)	MLD_{whole} (Gy)	$V5_{ipsi}$ (%)	$V20_{ipsi}$ (%)
													Avg	Range				
Aaron	2014	India	Supine	3D conformal	Static tangents	Mixed energy MV photons	No	No	Chest wall	50	25	10	NS	NS	NS	NS	NS	NS
			Supine	3D IMRT	Oblique fields	Low energy MV photons	No	No	Chest wall	50	25	10	NS	NS	NS	NS	NS	NS
			Supine	3D IMRT	Oblique fields	Low energy MV photons	No	No	Chest wall	50	25	10	NS	NS	NS	NS	NS	NS
			Supine	3D IMRT	Oblique fields	Low energy MV photons	No	No	Chest wall	50	25	10	NS	NS	NS	NS	NS	NS
Abo-Madyan	2014	Germany	Supine	3D Conformal plan	Static tangents	Low energy MV photons	No	No	Breast	50	25	10	7.4	NS	0.4	NS	NS	NS
			Supine	3D Conformal plan with FIF	Static tangents	Low energy MV photons	No	No	Breast	50	25	10	8.5	NS	0.4	NS	NS	NS
			Supine	3D Conformal plan with FIF	Static tangents	Low energy MV photons	No	No	Breast	50	25	10	8.5	NS	0.4	NS	NS	NS
			Supine	3D Conformal plan with FIF	Static tangents	Low energy MV photons	No	No	Breast	50	25	10	8.5	NS	0.4	NS	NS	NS
			Supine	3D IMRT	Static tangents	Low energy MV photons	No	No	Breast	50	25	10	9.5	NS	2.4	NS	NS	NS
			Supine	3D IMRT	Static tangents	Low energy MV photons	No	No	Breast	50	25	10	9.5	NS	2.4	NS	NS	NS
			Supine	3D IMRT	Static tangents	Low energy MV photons	No	No	Breast	50	25	10	9.5	NS	2.4	NS	NS	NS
			Supine	3D IMRT	Rotational fields	Low energy MV photons	No	No	Breast	50	25	10	9.9	NS	2.0	NS	NS	NS
			Supine	3D IMRT	Rotational fields	Low energy MV photons	No	No	Breast	50	25	10	9.9	NS	2.0	NS	NS	NS
			Supine	3D IMRT	Rotational fields	Low energy MV photons	No	No	Breast	50	25	10	9.9	NS	2.0	NS	NS	NS
Adam	2015	Romania	Supine	3D Conformal with FIF	Static tangents	Low energy MV photons	No	No	Breast	50	25	1	12.0	NS	NS	NS	NS	24.0
			Supine	3D IMRT	Rotational fields	Low energy MV photons	No	No	Breast	50	25	1	10.0	NS	NS	NS	NS	19.0
			Supine	3D IMRT	Rotational fields	Low energy MV photons	No	No	Breast	50	25	1	10.0	NS	NS	NS	NS	19.0
			Supine	3D IMRT	Rotational fields	Low energy MV photons	No	No	Breast	50	25	1	10.0	NS	NS	NS	NS	19.0
Akamatsu	2014	Japan	Supine	Carbon ions	Carbon ions	Carbon ions	Yes	Yes	Partial breast	52.8	4	1	NS	NS	NS	NS	NS	0.0
Alco	2010	Turkey	Supine	3D Conformal	Static tangents	Low energy MV photons	No	No	Breast	50	25	30	6.5	2.5-11.2	NS	NS	17.2	10.5
			Supine	3D Conformal	Static tangents	Low energy MV photons	No	No	Breast; axilla	50	25	30	9.6	6.1-15.8	NS	NS	30.3	16.8
			Supine	3D Conformal	Static tangents	Low energy MV photons	No	No	Breast; axilla	50	25	30	9.6	6.1-15.8	NS	NS	30.3	16.8
			Supine	3D Conformal	Static tangents	Low energy MV photons	No	No	Breast; axilla	50	25	30	9.6	6.1-15.8	NS	NS	30.3	16.8
Al-Rahbi	2013	Oman	Supine	3D Conformal	Static tangents	Low energy MV photons	No	Yes	Breast or chest wall	45-50	20-25	20	NS	NS	NS	NS	NS	NS
			Supine	3D Conformal with FIF	Static tangents	Low energy MV photons	No	No	Breast or chest wall	45-50	20-25	20	NS	NS	NS	NS	NS	NS
			Supine	3D Conformal with FIF	Static tangents	Low energy MV photons	No	No	Breast or chest wall	45-50	20-25	20	NS	NS	NS	NS	NS	NS
			Supine	3D Conformal with FIF	Static tangents	Low energy MV photons	No	No	Breast or chest wall	45-50	20-25	20	NS	NS	NS	NS	NS	NS
			Supine	3D IMRT	Static tangents	Low energy MV photons	No	No	Breast or chest wall	45-50	20-25	20	NS	NS	NS	NS	NS	NS
			Supine	3D IMRT	Static tangents	Low energy MV photons	No	No	Breast or chest wall	45-50	20-25	20	NS	NS	NS	NS	NS	NS
			Supine	3D IMRT	Static tangents	Low energy MV photons	No	No	Breast or chest wall	45-50	20-25	20	NS	NS	NS	NS	NS	NS
Aly	2015	Germany	Supine	3D Conformal with FIF	Static tangents	Low energy MV photons	No	No	Breast + SIB	50.4	28	12	8.4	NS	0.9	NS	NS	14.1
			Supine	3D Conformal with FIF	Static tangents	Low energy MV photons	No	No	Breast + SIB	50.4	28	12	8.8	NS	0.7	NS	NS	13.7
			Supine	3D Conformal with FIF	Static tangents	Low energy MV photons	No	No	Breast + SIB	50.4	28	12	8.8	NS	0.7	NS	NS	13.7
			Supine	3D Conformal with FIF	Static tangents	Low energy MV photons	No	No	Breast + SIB	50.4	28	12	8.8	NS	0.7	NS	NS	13.7
			Supine	3D IMRT	Static tangents	Low energy MV photons	No	No	Breast + SIB	50.4	28	12	9.1	NS	1.1	NS	NS	14.8
			Supine	3D IMRT	Static tangents	Low energy MV photons	No	No	Breast + SIB	50.4	28	12	9.1	NS	1.1	NS	NS	14.8
			Supine	3D IMRT	Static tangents	Low energy MV photons	No	No	Breast + SIB	50.4	28	12	9.1	NS	1.1	NS	NS	14.8
			Supine	3D IMRT	Rotational fields	Low energy MV photons	No	No	Breast + SIB	50.4	28	12	9.5	NS	1.8	NS	NS	13.3
			Supine	3D IMRT	Rotational fields	Low energy MV photons	No	No	Breast + SIB	50.4	28	12	9.5	NS	1.8	NS	NS	13.3

Table E3 continued on the next page.

Table E3 continued from the previous page.

Author	Year	Country	Description of regimens*									No. of CT plans	Lung dose measures					
			Patient position	Treatment planning	Field type	Beam energy & modality	Breath. adapt.	RT plan delivered	Target(s)	Prescr. dose	No. of fraction		MLD_{ipsi} (Gy)		MLD_{cont} (Gy)	MLD_{whole} (Gy)	$V5_{ipsi}$ (%)	$V20_{ipsi}$ (%)
													Avg	Range				
			Supine	3D IMRT	Rotational fields	Low energy MV photons	No	No	Breast + SIB	50.4	28	12	9.5	NS	1.8	NS	NS	13.3
Amoush	2015	USA	Supine	3D Conformal with FIF	Static tangents	MV photons	No	No	Breast; SCF	50	25	15	NS	NS	NS	NS	NS	NS
			Supine	3D Conformal with FIF	Static tangents	MV photons	No	No	Breast; SCF	50	25	15	NS	NS	NS	NS	NS	NS
			Supine	3D Conformal with FIF	Static tangents	MV photons	No	No	Breast; SCF	50	25	15	NS	NS	NS	NS	NS	NS
			Supine	3D Conformal with FIF	Static tangents	MV photons	No	No	Breast; SCF	50	25	15	NS	NS	NS	NS	NS	NS
Anbumani	2012	India	Supine	3D Conformal	Oblique fields	Low energy MV photons	No	No	Partial breast	30	5	15	NS	NS	NS	5.1	NS	NS
			Supine	Brachytherapy	Brachytherapy	Brachytherapy	No	Yes	Partial breast	50	25	15	NS	NS	NS	4.0	NS	NS
			Supine	Brachytherapy	Brachytherapy	Brachytherapy	No	Yes	Partial breast	50	25	15	NS	NS	NS	4.0	NS	NS
			Supine	Brachytherapy	Brachytherapy	Brachytherapy	No	Yes	Partial breast	50	25	15	NS	NS	NS	4.0	NS	NS
Arenas	2014	Spain	Supine	3D Conformal	Static tangents with FIF	Low energy MV photons	No	Yes	Breast	50	25	12	3.0	NS	NS	NS	NS	1.7
			Supine	3D Conformal	Static tangents with FIF	Low energy MV photons	No	No	Breast	50	25	12	8.0	NS	NS	NS	NS	13.7
			Supine	3D Conformal	Static tangents with FIF	Low energy MV photons	No	No	Breast	50	25	12	8.0	NS	NS	NS	NS	13.7
			Supine	3D Conformal	Static tangents with FIF	Low energy MV photons	No	No	Breast	50	25	12	8.0	NS	NS	NS	NS	13.7
Ares	2010	Switzerland	Supine	3D Conformal plan	Tangents plus boost/direct fields	Low energy MV photons	No	No	Breast	50	NS	10	8.5	NS	0.5	NS	NS	14.0
			Supine	3D IMRT	Tangents plus boost/direct fields	Protons	No	No	Breast	50	NS	10	3.5	NS	0.0	NS	NS	7.0
			Supine	3D IMRT	Tangents plus boost/direct fields	Protons	No	No	Breast	50	NS	10	3.5	NS	0.0	NS	NS	7.0
			Supine	3D IMRT	Tangents plus boost/direct fields	Protons	No	No	Breast	50	NS	10	3.5	NS	0.0	NS	NS	7.0
			Supine	3D IMRT	Tangents plus boost/direct fields	Low energy MV photons	No	No	Breast	50	NS	10	7.5	NS	2.0	NS	NS	9.0
			Supine	3D IMRT	Tangents plus boost/direct fields	Low energy MV photons	No	No	Breast	50	NS	10	7.5	NS	2.0	NS	NS	9.0
			Supine	3D IMRT	Tangents plus boost/direct fields	Low energy MV photons	No	No	Breast	50	NS	10	7.5	NS	2.0	NS	NS	9.0
			Supine	3D Conformal plan	Tangents plus boost/direct fields	Low energy MV photons	No	No	Chest wall	50	NS	10	9.0	NS	0.5	NS	NS	16.0
			Supine	3D Conformal plan	Tangents plus boost/direct fields	Low energy MV photons	No	No	Chest wall	50	NS	10	9.0	NS	0.5	NS	NS	16.0
			Supine	3D Conformal plan	Tangents plus boost/direct fields	Low energy MV photons	No	No	Chest wall	50	NS	10	9.0	NS	0.5	NS	NS	16.0
			Supine	3D IMRT	Tangents plus boost/direct fields	Protons	No	No	Chest wall	50	NS	10	3.0	NS	0.0	NS	NS	7.0
			Supine	3D IMRT	Tangents plus boost/direct fields	Protons	No	No	Chest wall	50	NS	10	3.0	NS	0.0	NS	NS	7.0
			Supine	3D IMRT	Tangents plus boost/direct fields	Protons	No	No	Chest wall	50	NS	10	3.0	NS	0.0	NS	NS	7.0
			Supine	3D IMRT	Tangents plus boost/direct fields	Low energy MV photons	No	No	Chest wall	50	NS	10	7.5	NS	2.5	NS	NS	10.0
			Supine	3D IMRT	Tangents plus boost/direct fields	Low energy MV photons	No	No	Chest wall	50	NS	10	7.5	NS	2.5	NS	NS	10.0

Table E3 continued on the next page.

Table E3 continued from the previous page.

Author	Year	Country	Description of regimens*: Patient position	Treatment planning	Field type	Beam energy & modality	Breath. adapt.	RT plan delivered	Target(s)	Prescr. dose	No. of fraction	No. of CT plans	Lung dose measures: MLD_{ipsi} (Gy) Avg	MLD_{ipsi} (Gy) Range	MLD_{cont} (Gy)	MLD_{whole} (Gy)	$V5_{ipsi}$ (%)	$V20_{ipsi}$ (%)
			Supine	3D IMRT	Tangents plus boost/direct fields	Low energy MV photons	No	No	Chest wall	50	NS	10	7.5	NS	2.5	NS	NS	10.0
			Supine	3D Conformal plan	Tangents plus boost/direct fields	Low energy MV photons	No	No	Breast; SCF; axilla	50	NS	10	12.5	NS	1.0	NS	NS	23.0
			Supine	3D Conformal plan	Tangents plus boost/direct fields	Low energy MV photons	No	No	Breast; SCF; axilla	50	NS	10	12.5	NS	1.0	NS	NS	23.0
			Supine	3D Conformal plan	Tangents plus boost/direct fields	Low energy MV photons	No	No	Breast; SCF; axilla	50	NS	10	12.5	NS	1.0	NS	NS	23.0
			Supine	3D IMRT	Tangents plus boost/direct fields	Protons	No	No	Breast; SCF; axilla	50	NS	10	4.5	NS	0.0	NS	NS	9.0
			Supine	3D IMRT	Tangents plus boost/direct fields	Protons	No	No	Breast; SCF; axilla	50	NS	10	4.5	NS	0.0	NS	NS	9.0
			Supine	3D IMRT	Tangents plus boost/direct fields	Protons	No	No	Breast; SCF; axilla	50	NS	10	4.5	NS	0.0	NS	NS	9.0
			Supine	3D IMRT	Tangents plus boost/direct fields	Low energy MV photons	No	No	Breast; SCF; axilla	50	NS	10	9.0	NS	3.0	NS	NS	11.0
			Supine	3D IMRT	Tangents plus boost/direct fields	Low energy MV photons	No	No	Breast; SCF; axilla	50	NS	10	9.0	NS	3.0	NS	NS	11.0
			Supine	3D IMRT	Tangents plus boost/direct fields	Low energy MV photons	No	No	Breast; SCF; axilla	50	NS	10	9.0	NS	3.0	NS	NS	11.0
			Supine	3D Conformal plan	Tangents plus boost/direct fields	Low energy MV photons	No	No	Chest wall; SCF; axilla	50	NS	10	13.5	NS	2.0	NS	NS	28.0
			Supine	3D Conformal plan	Tangents plus boost/direct fields	Low energy MV photons	No	No	Chest wall; SCF; axilla	50	NS	10	13.5	NS	2.0	NS	NS	28.0
			Supine	3D Conformal plan	Tangents plus boost/direct fields	Low energy MV photons	No	No	Chest wall; SCF; axilla	50	NS	10	13.5	NS	2.0	NS	NS	28.0
			Supine	3D IMRT	Tangents plus boost/direct fields	Protons	No	No	Chest wall; SCF; axilla	50	NS	10	9.5	NS	0.0	NS	NS	10.0
			Supine	3D IMRT	Tangents plus boost/direct fields	Protons	No	No	Chest wall; SCF; axilla	50	NS	10	9.5	NS	0.0	NS	NS	10.0
			Supine	3D IMRT	Tangents plus boost/direct fields	Protons	No	No	Chest wall; SCF; axilla	50	NS	10	9.5	NS	0.0	NS	NS	10.0
			Supine	3D IMRT	Tangents plus boost/direct fields	Low energy MV photons	No	No	Chest wall; SCF; axilla	50	NS	10	5.0	NS	3.5	NS	NS	14.0
			Supine	3D IMRT	Tangents plus boost/direct fields	Low energy MV photons	No	No	Chest wall; SCF; axilla	50	NS	10	5.0	NS	3.5	NS	NS	14.0
			Supine	3D IMRT	Tangents plus boost/direct fields	Low energy MV photons	No	No	Chest wall; SCF; axilla	50	NS	10	5.0	NS	3.5	NS	NS	14.0
			Supine	3D Conformal plan	Tangents plus boost/direct fields	Low energy MV photons	No	No	Breast; SCF; axilla; IMC	50	NS	10	21.0	NS	27.5	NS	NS	39.0
			Supine	3D Conformal plan	Tangents plus boost/direct fields	Low energy MV photons	No	No	Breast; SCF; axilla; IMC	50	NS	10	21.0	NS	27.5	NS	NS	39.0
			Supine	3D Conformal plan	Tangents plus boost/direct fields	Low energy MV photons	No	No	Breast; SCF; axilla; IMC	50	NS	10	21.0	NS	27.5	NS	NS	39.0
			Supine	3D IMRT	Tangents plus boost/direct fields	Protons	No	No	Breast; SCF; axilla; IMC	50	NS	10	7.0	NS	0.5	NS	NS	15.0
			Supine	3D IMRT	Tangents plus boost/direct fields	Protons	No	No	Breast; SCF; axilla; IMC	50	NS	10	7.0	NS	0.5	NS	NS	15.0
			Supine	3D IMRT	Tangents plus boost/direct fields	Protons	No	No	Breast; SCF; axilla; IMC	50	NS	10	7.0	NS	0.5	NS	NS	15.0
			Supine	3D IMRT	Tangents plus boost/direct fields	Low energy MV photons	No	No	Breast; SCF; axilla; IMC	50	NS	10	13.0	NS	6.0	NS	NS	19.0
			Supine	3D IMRT	Tangents plus boost/direct fields	Low energy MV photons	No	No	Breast; SCF; axilla; IMC	50	NS	10	13.0	NS	6.0	NS	NS	19.0
			Supine	3D IMRT	Tangents plus boost/direct fields	Low energy MV photons	No	No	Breast; SCF; axilla; IMC	50	NS	10	13.0	NS	6.0	NS	NS	19.0

Table E3 continued on the next page.

Table E3 continued from the previous page.

Author	Year	Country	Description of regimens*										No. of CT plans	Lung dose measures					
			Patient position	Treatment planning	Field type	Beam energy & modality	Breath. adapt.	RT plan delivered	Target(s)	Prescr. dose	No. of fraction			MLD_{ipsi} (Gy)		MLD_{cont} (Gy)	MLD_{whole} (Gy)	$V5_{ipsi}$ (%)	$V20_{ipsi}$ (%)
														Avg	Range				
			Supine	3D Conformal plan	Tangents plus boost/direct fields	Low energy MV photons	No	No	Chest wall; SCF; axilla; IMC	50	NS		10	20.0	NS	1.5	NS	NS	39.0
			Supine	3D Conformal plan	Tangents plus boost/direct fields	Low energy MV photons	No	No	Chest wall; SCF; axilla; IMC	50	NS		10	20.0	NS	1.5	NS	NS	39.0
			Supine	3D Conformal plan	Tangents plus boost/direct fields	Low energy MV photons	No	No	Chest wall; SCF; axilla; IMC	50	NS		10	20.0	NS	1.5	NS	NS	39.0
			Supine	3D IMRT	Tangents plus boost/direct fields	Protons	No	No	Chest wall; SCF; axilla; IMC	50	NS		10	13.5	NS	0.5	NS	NS	15.0
			Supine	3D IMRT	Tangents plus boost/direct fields	Protons	No	No	Chest wall; SCF; axilla; IMC	50	NS		10	13.5	NS	0.5	NS	NS	15.0
			Supine	3D IMRT	Tangents plus boost/direct fields	Protons	No	No	Chest wall; SCF; axilla; IMC	50	NS		10	13.5	NS	0.5	NS	NS	15.0
			Supine	3D IMRT	Tangents plus boost/direct fields	Low energy MV photons	No	No	Chest wall; SCF; axilla; IMC	50	NS		10	7.5	NS	7.0	NS	NS	22.0
			Supine	3D IMRT	Tangents plus boost/direct fields	Low energy MV photons	No	No	Chest wall; SCF; axilla; IMC	50	NS		10	7.5	NS	7.0	NS	NS	22.0
			Supine	3D IMRT	Tangents plus boost/direct fields	Low energy MV photons	No	No	Chest wall; SCF; axilla; IMC	50	NS		10	7.5	NS	7.0	NS	NS	22.0
Arora	2015	USA	Supine	3D Conformal	Static tangents	MV photons	No	Yes	Chest wall	50	25		25	NS	NS	NS	NS	NS	NS
			Supine	3D Conformal	Static tangents	MV photons	No	Yes	Chest wall	50	25		25	NS	NS	NS	NS	NS	NS
			Supine	3D Conformal	Static tangents	MV photons	No	Yes	Chest wall	50	25		25	NS	NS	NS	NS	NS	NS
			Supine	3D Conformal	Static tangents	MV photons	No	Yes	Chest wall	50	25		25	NS	NS	NS	NS	NS	NS
Badakshi	2013	Germany	Supine	3D IMRT	Rotational fields	Low energy MV photons	No	NS	Breast +/- SCF	50	25		8	12.1	NS	0.7	NS	94.2	29.2
			Supine	3D IMRT	Oblique fields	Low energy MV photons	No	NS	Breast +/- SCF	50	25		8	12.1	NS	0.7	NS	68.3	19.9
			Supine	3D IMRT	Oblique fields	Low energy MV photons	No	NS	Breast +/- SCF	50	25		8	12.1	NS	0.7	NS	68.3	19.9
			Supine	3D IMRT	Oblique fields	Low energy MV photons	No	NS	Breast +/- SCF	50	25		8	12.1	NS	0.7	NS	68.3	19.9
			Supine	3D Conformal	Static tangents	Low energy MV photons	No	NS	Breast +/- SCF	50	25		8	12.1	NS	0.7	NS	40.7	22.5
			Supine	3D Conformal	Static tangents	Low energy MV photons	No	NS	Breast +/- SCF	50	25		8	12.1	NS	0.7	NS	40.7	22.5
			Supine	3D Conformal	Static tangents	Low energy MV photons	No	NS	Breast +/- SCF	50	25		8	12.1	NS	0.7	NS	40.7	22.5
Banaei	2015	Iran	Supine	3D Conformal	Static tangents	Low energy MV photons	No	NS	Chest wall; SCF	50	25		18	13.0	9.2-19.3	NS	NS	NS	NS
			Supine	3D Conformal	Static tangents	Low energy MV photons	No	NS	Chest wall; SCF	50	25		18	13.7	9-19.4	NS	NS	NS	NS
			Supine	3D Conformal	Static tangents	Low energy MV photons	No	NS	Chest wall; SCF	50	25		18	13.7	9-19.4	NS	NS	NS	NS
			Supine	3D Conformal	Static tangents	Low energy MV photons	No	NS	Chest wall; SCF	50	25		18	13.7	9-19.4	NS	NS	NS	NS
Barsoum	2015	Egypt	Supine	2D	Static tangents	MV photons	No	No	Breast or Chest wall; SCF	50	25		8	6.8	NS	NS	NS	NS	NS
			Supine	3D Conformal	Static tangents	MV photons	No	No	Breast or Chest wall; SCF	50	25		8	10.1	NS	NS	NS	NS	NS
			Supine	3D Conformal	Static tangents	MV photons	No	No	Breast or Chest wall; SCF	50	25		8	10.1	NS	NS	NS	NS	NS
			Supine	3D Conformal	Static tangents	MV photons	No	No	Breast or Chest wall; SCF	50	25		8	10.1	NS	NS	NS	NS	NS
			Supine	2D	Static tangents	MV photons	No	No	Breast or Chest wall; SCF	50	25		7	7.9	NS	NS	NS	NS	NS
			Supine	2D	Static tangents	MV photons	No	No	Breast or Chest wall; SCF	50	25		7	7.9	NS	NS	NS	NS	NS
			Supine	2D	Static tangents	MV photons	No	No	Breast or Chest wall; SCF	50	25		7	7.9	NS	NS	NS	NS	NS

Table E3 continued on the next page.

Table E3 continued from the previous page.

Author	Year	Country	Description of regimens*									No. of CT plans	Lung dose measures					
			Patient position	Treatment planning	Field type	Beam energy & modality	Breath. adapt.	RT plan delivered	Target(s)	Prescr. dose	No. of fraction		MLD_{ipsi} (Gy)		MLD_{cont} (Gy)	MLD_{whole} (Gy)	$V5_{ipsi}$ (%)	$V20_{ipsi}$ (%)
													Avg	Range				
			Supine	3D Conformal	Static tangents	MV photons	No	No	Breast or Chest wall; SCF	50	25	7	10.2	NS	NS	NS	NS	NS
			Supine	3D Conformal	Static tangents	MV photons	No	No	Breast or Chest wall; SCF	50	25	7	10.2	NS	NS	NS	NS	NS
			Supine	3D Conformal	Static tangents	MV photons	No	No	Breast or Chest wall; SCF	50	25	7	10.2	NS	NS	NS	NS	NS
Bartlett	2014	UK	Supine	3D Conformal	Static tangents	Mixed energy MV photons	Yes	Yes	Breast	40	15	28	3.7	3.4-4.0	NS	1.8	NS	NS
			Prone	3D Conformal	Static tangents	Mixed energy MV photons	No	Yes	Breast	40	15	28	0.3	0.3-0.4	NS	0.2	NS	NS
			Prone	3D Conformal	Static tangents	Mixed energy MV photons	No	Yes	Breast	40	15	28	0.3	0.3-0.4	NS	0.2	NS	NS
			Prone	3D Conformal	Static tangents	Mixed energy MV photons	No	Yes	Breast	40	15	28	0.3	0.3-0.4	NS	0.2	NS	NS
Belkacemi	2014	France	Supine	3D Conformal	Static tangents	MV photons	No	NS	Breast	40-50	NS	25	5.0	NS	NS	NS	20.0	6.0
			Supine	3D Conformal	Static tangents	MV photons	No	NS	Breast	40-50	NS	25	6.0	NS	NS	NS	26.0	7.0
			Supine	3D Conformal	Static tangents	MV photons	No	NS	Breast	40-50	NS	25	6.0	NS	NS	NS	26.0	7.0
			Supine	3D Conformal	Static tangents	MV photons	No	NS	Breast	40-50	NS	25	6.0	NS	NS	NS	26.0	7.0
Blom Glodman Acta	2014	Sweden	Supine	3D Conformal	Static tangents	Mixed photons & electrons	No	Yes	Breast or Chest wall; IMC; axilla; SCF	46-50	23-25	60	NS	NS	NS	NS	NS	30.4
Blom Glodman JRP	2014	Sweden	Supine	3D Conformal	Static tangents	Mixed photons & electrons	No	Yes	Breast or Chest wall; SCF; +/- IMC	46-50	23-25	89	NS	NS	NS	NS	NS	26.0
Bodacs	2014	Hungary	Supine	3D Conformal	Oblique fields	Low energy MV photons	No	No	Partial breast	36.9	9	30	NS	NS	NS	NS	NS	NS
			Supine	3D Conformal	Static tangents	Low energy MV photons	No	No	Breast	50	25	30	NS	NS	NS	NS	NS	NS
			Supine	3D Conformal	Static tangents	Low energy MV photons	No	No	Breast	50	25	30	NS	NS	NS	NS	NS	NS
			Supine	3D Conformal	Static tangents	Low energy MV photons	No	No	Breast	50	25	30	NS	NS	NS	NS	NS	NS
			Supine	Brachytherapy	Brachytherapy	Brachytherapy	No	No	Brachytherapy	30.1	7	30	NS	NS	NS	NS	NS	NS
			Supine	Brachytherapy	Brachytherapy	Brachytherapy	No	No	Brachytherapy	30.1	7	30	NS	NS	NS	NS	NS	NS
			Supine	Brachytherapy	Brachytherapy	Brachytherapy	No	No	Brachytherapy	30.1	7	30	NS	NS	NS	NS	NS	NS
Bolukbasi	2014	Turkey	Supine	3D Conformal with FIF	Static tangents	Mixed energy MV photons	Yes	No	Breast	50	25	10	5.1	3.6-6.9	NS	NS	19.9	9.2
			Supine	3D Conformal with FIF	Static tangents	Mixed energy MV photons	No	No	Breast	50	25	10	5.9	3.0-7.6	NS	NS	22.6	11.3
			Supine	3D Conformal with FIF	Static tangents	Mixed energy MV photons	No	No	Breast	50	25	10	5.9	3.0-7.6	NS	NS	22.6	11.3
			Supine	3D Conformal with FIF	Static tangents	Mixed energy MV photons	No	No	Breast	50	25	10	5.9	3.0-7.6	NS	NS	22.6	11.3
			Supine	3D IMRT	Oblique fields	Low energy MV photons	Yes	No	Breast	50	25	10	6.7	4.7-9.9	NS	NS	28.7	10.3
			Supine	3D IMRT	Oblique fields	Low energy MV photons	Yes	No	Breast	50	25	10	6.7	4.7-9.9	NS	NS	28.7	10.3
			Supine	3D IMRT	Oblique fields	Low energy MV photons	Yes	No	Breast	50	25	10	6.7	4.7-9.9	NS	NS	28.7	10.3
			Supine	3D IMRT	Oblique fields	Low energy MV photons	No	No	Breast	50	25	10	7.2	4.4-10.9	NS	NS	29.0	11.7
			Supine	3D IMRT	Oblique fields	Low energy MV photons	No	No	Breast	50	25	10	7.2	4.4-10.9	NS	NS	29.0	11.7
			Supine	3D IMRT	Oblique fields	Low energy MV photons	No	No	Breast	50	25	10	7.2	4.4-10.9	NS	NS	29.0	11.7
Borca	2012	Italy	Supine	3D Conformal plan	Static tangents	Mixed energy MV photons	No	No	Breast	50	25	17	6.9	NS	NS	NS	21.6	13.2
			Supine	3D Conformal plan with FIF	Static tangents	Low energy MV photons	No	No	Breast	50	25	17	6.3	NS	NS	NS	22.4	11.7
			Supine	3D Conformal plan with FIF	Static tangents	Low energy MV photons	No	No	Breast	50	25	17	6.3	NS	NS	NS	22.4	11.7
			Supine	3D Conformal plan with FIF	Static tangents	Low energy MV photons	No	No	Breast	50	25	17	6.3	NS	NS	NS	22.4	11.7
			Supine	3D IMRT	Static tangents	Low energy MV photons	No	No	Breast	50	25	17	5.6	NS	NS	NS	18.7	9.9
			Supine	3D IMRT	Static tangents	Low energy MV photons	No	No	Breast	50	25	17	5.6	NS	NS	NS	18.7	9.9

Table E3 continued on the next page.

Table E3 continued from the previous page.

Author	Year	Country	Description of regimens*									No. of CT plans	Lung dose measures					
			Patient position	Treatment planning	Field type	Beam energy & modality	Breath. adapt.	RT plan delivered	Target(s)	Prescr. dose	No. of fraction		MLD_{ipsi} (Gy)		MLD_{cont} (Gy)	MLD_{whole} (Gy)	$V5_{ipsi}$ (%)	$V20_{ipsi}$ (%)
													Avg	Range				
			Supine	3D IMRT	Static tangents	Low energy MV photons	No	No	Breast	50	25	17	5.6	NS	NS	NS	18.7	9.9
Borges	2014	Portugal	Supine	3D Conformal with FIF	Static tangents	Low energy MV photons	No	No	Breast	50	25	7	9.0	NS	1.9	NS	23.5	16.3
			Supine	3D IMRT	Static tangents	Low energy MV photons	No	No	Breast	50	25	7	9.5	NS	0.6	NS	27.6	18.6
			Supine	3D IMRT	Static tangents	Low energy MV photons	No	No	Breast	50	25	7	9.5	NS	0.6	NS	27.6	18.6
			Supine	3D IMRT	Static tangents	Low energy MV photons	No	No	Breast	50	25	7	9.5	NS	0.6	NS	27.6	18.6
			Supine	3D IMRT	Oblique fields	Low energy MV photons	No	No	Breast	50	25	7	14.2	NS	9.0	NS	82.1	19.5
			Supine	3D IMRT	Oblique fields	Low energy MV photons	No	No	Breast	50	25	7	14.2	NS	9.0	NS	82.1	19.5
			Supine	3D IMRT	Oblique fields	Low energy MV photons	No	No	Breast	50	25	7	14.2	NS	9.0	NS	82.1	19.5
			Supine	3D Conformal	Rotational fields	Low energy MV photons	No	No	Breast	50	25	7	14.3	NS	0.8	NS	57.5	27.7
			Supine	3D Conformal	Rotational fields	Low energy MV photons	No	No	Breast	50	25	7	14.3	NS	0.8	NS	57.5	27.7
			Supine	3D Conformal	Rotational fields	Low energy MV photons	No	No	Breast	50	25	7	14.3	NS	0.8	NS	57.5	27.7
			Supine	3D Conformal with FIF	Static tangents	Low energy MV photons	No	No	Breast	50	25	7	13.7	NS	3.9	NS	26.5	16.3
			Supine	3D Conformal with FIF	Static tangents	Low energy MV photons	No	No	Breast	50	25	7	13.7	NS	3.9	NS	26.5	16.3
			Supine	3D Conformal with FIF	Static tangents	Low energy MV photons	No	No	Breast	50	25	7	13.7	NS	3.9	NS	26.5	16.3
			Supine	3D IMRT	Static tangents	Low energy MV photons	No	No	Breast	50	25	7	14.0	NS	2.1	NS	30.7	18.7
			Supine	3D IMRT	Static tangents	Low energy MV photons	No	No	Breast	50	25	7	14.0	NS	2.1	NS	30.7	18.7
			Supine	3D IMRT	Static tangents	Low energy MV photons	No	No	Breast	50	25	7	14.0	NS	2.1	NS	30.7	18.7
			Supine	3D IMRT	Oblique fields	Low energy MV photons	No	No	Breast	50	25	7	16.9	NS	9.2	NS	81.8	20.6
			Supine	3D IMRT	Oblique fields	Low energy MV photons	No	No	Breast	50	25	7	16.9	NS	9.2	NS	81.8	20.6
			Supine	3D IMRT	Oblique fields	Low energy MV photons	No	No	Breast	50	25	7	16.9	NS	9.2	NS	81.8	20.6
			Supine	3D Conformal	Rotational fields	Low energy MV photons	No	No	Breast	50	25	7	9.0	NS	8.4	NS	57.4	27.0
			Supine	3D Conformal	Rotational fields	Low energy MV photons	No	No	Breast	50	25	7	9.0	NS	8.4	NS	57.4	27.0
			Supine	3D Conformal	Rotational fields	Low energy MV photons	No	No	Breast	50	25	7	9.0	NS	8.4	NS	57.4	27.0
Bourgier	2010	France	Supine	3D Conformal	Static tangents plus electron field	Mixed photons & electrons	No	Yes	Partial breast	40	10	25	1.6	1.0-2.3	NS	NS	NS	NS
Bruzzaniti	2013	Italy	Supine	3D Conformal	Static tangents	MV photons	Yes	NS	Breast	50	25	8	4.6	3.3-6.1	NS	NS	NS	6.1
			Supine	3D Conformal	Static tangents	MV photons	No	No	Breast	50	25	8	5.5	3.5-8.8	NS	NS	NS	8.1
			Supine	3D Conformal	Static tangents	MV photons	No	No	Breast	50	25	8	5.5	3.5-8.8	NS	NS	NS	8.1
			Supine	3D Conformal	Static tangents	MV photons	No	No	Breast	50	25	8	5.5	3.5-8.8	NS	NS	NS	8.1
			Supine	3D Conformal	Static tangents	MV photons	Yes	NS	Breast	34	3.4	8	3.2	2.3-4.2	NS	NS	NS	5.7
			Supine	3D Conformal	Static tangents	MV photons	Yes	NS	Breast	34	3.4	8	3.2	2.3-4.2	NS	NS	NS	5.7
			Supine	3D Conformal	Static tangents	MV photons	Yes	NS	Breast	34	3.4	8	3.2	2.3-4.2	NS	NS	NS	5.7
			Supine	3D Conformal	Static tangents	MV photons	No	No	Breast	34	3.4	8	3.4	2.4-6.0	NS	NS	NS	7.7
			Supine	3D Conformal	Static tangents	MV photons	No	No	Breast	34	3.4	8	3.4	2.4-6.0	NS	NS	NS	7.7
			Supine	3D Conformal	Static tangents	MV photons	No	No	Breast	34	3.4	8	3.4	2.4-6.0	NS	NS	NS	7.7
Cammarota	2014	Italy	Supine	3D IMRT	Rotational fields	Low energy MV photons	No	No	Breast	42.56	16	12	NS	NS	NS	3.9	NS	NS
			Prone	3D Conformal	Static tangents	Low energy MV photons	No	No	Breast	42.56	16	12	NS	NS	NS	0.4	NS	NS
			Prone	3D Conformal	Static tangents	Low energy MV photons	No	No	Breast	42.56	16	12	NS	NS	NS	0.4	NS	NS
			Prone	3D Conformal	Static tangents	Low energy MV photons	No	No	Breast	42.56	16	12	NS	NS	NS	0.4	NS	NS
Capezzali	2013	France	Lateral Decubitus	3D Conformal	Static tangents	Low energy MV photons	No	Yes	Breast	50	25	33	1.0	0.6-1.6	NS	NS	NS	NS
			Lateral Decubitus	3D Conformal	Static tangents	Low energy MV photons	No	Yes	Breast	41.6	13	17	0.7	0.2-1.9	NS	NS	NS	NS
			Lateral Decubitus	3D Conformal	Static tangents	Low energy MV photons	No	Yes	Breast	41.6	13	17	0.7	0.2-1.9	NS	NS	NS	NS

Table E3 continued on the next page.

Table E3 continued from the previous page.

Author	Year	Country	Patient position	Treatment planning	Field type	Beam energy & modality	Breath. adapt.	RT plan delivered	Target(s)	Prescr. dose	No. of fraction	No. of CT plans	MLD_{ipsi} (Gy) Avg	MLD_{ipsi} (Gy) Range	MLD_{cont} (Gy)	MLD_{whole} (Gy)	$V5_{ipsi}$ (%)	$V20_{ipsi}$ (%)
			Description of regimens*										Lung dose measures					
			Lateral Decubitus	3D Conformal	Static tangents	Low energy MV photons	No	Yes	Breast	41.6	13	17	0.7	0.2-1.9	NS	NS	NS	NS
Catli	2014	Turkey	Supine	3D Conformal	Static tangents	Low energy MV photons	No	NS	Breast	50	25	20	NS	NS	NS	4.5	NS	NS
			Supine	3D Conformal	Static tangents	Low energy MV photons	No	NS	Breast	50	25	20	NS	NS	NS	4.7	NS	NS
			Supine	3D Conformal	Static tangents	Low energy MV photons	No	NS	Breast	50	25	20	NS	NS	NS	4.7	NS	NS
			Supine	3D Conformal	Static tangents	Low energy MV photons	No	NS	Breast	50	25	20	NS	NS	NS	4.7	NS	NS
			Supine	3D Conformal	Oblique fields	Low energy MV photons	No	NS	Breast	50	25	20	NS	NS	NS	3.3	NS	NS
			Supine	3D Conformal	Oblique fields	Low energy MV photons	No	NS	Breast	50	25	20	NS	NS	NS	3.3	NS	NS
			Supine	3D Conformal	Oblique fields	Low energy MV photons	No	NS	Breast	50	25	20	NS	NS	NS	3.3	NS	NS
Caudrelier	2014	Canada	Supine	3D IMRT	Rotational fields	Low energy MV photons	No	Yes	Chest wall or breast; SCF; IMC; axilla	50	25	18	10.2	NS	5.3	NS	49.8	16.0
Cendales	2012	Colombia	Supine	3D IMRT	Static tangents	Low energy MV photons	No	No	Breast	50	25	1	11.8	NS	3.4	7.1	NS	NS
			Supine	3D IMRT	Tangents plus boost/direct fields	Low energy MV photons	No	Yes	Breast	50	25	1	12.5	NS	3.1	7.2	NS	NS
			Supine	3D IMRT	Tangents plus boost/direct fields	Low energy MV photons	No	Yes	Breast	50	25	1	12.5	NS	3.1	7.2	NS	NS
			Supine	3D IMRT	Tangents plus boost/direct fields	Low energy MV photons	No	Yes	Breast	50	25	1	12.5	NS	3.1	7.2	NS	NS
			Supine	3D Conformal	Tangents plus boost/direct fields	Low energy MV photons	No	No	Breast	50	25	1	27.5	NS	0.5	12.3	NS	NS
			Supine	3D Conformal	Tangents plus boost/direct fields	Low energy MV photons	No	No	Breast	50	25	1	27.5	NS	0.5	12.3	NS	NS
			Supine	3D Conformal	Tangents plus boost/direct fields	Low energy MV photons	No	No	Breast	50	25	1	27.5	NS	0.5	12.3	NS	NS
			Supine	3D Conformal	Static tangents plus electron field	Mixed photons & electrons	No	No	Breast	50	25	1	12.7	NS	0.2	5.7	NS	NS
			Supine	3D Conformal	Static tangents plus electron field	Mixed photons & electrons	No	No	Breast	50	25	1	12.7	NS	0.2	5.7	NS	NS
			Supine	3D Conformal	Static tangents plus electron field	Mixed photons & electrons	No	No	Breast	50	25	1	12.7	NS	0.2	5.7	NS	NS
Chamunyonga	2014	UK	Supine	3D Conformal with FIF	Static tangents	Mixed energy MV photons	No	Yes	Breast	50	25	12	8.9	5.4-11.6	NS	NS	NS	16.8
Chan	2015	Singapore	Supine	3D Conformal with FIF	Static tangents	MV photons	No	No	Breast	50	25	15	10.7	NS	NS	NS	35.9	21.8
			Supine	Brachytherapy	Brachytherapy	Brachytherapy	No	Yes	Partial breast	34	10	15	2.3	NS	NS	NS	23.9	1.0
			Supine	Brachytherapy	Brachytherapy	Brachytherapy	No	Yes	Partial breast	34	10	15	2.3	NS	NS	NS	23.9	1.0
			Supine	Brachytherapy	Brachytherapy	Brachytherapy	No	Yes	Partial breast	34	10	15	2.3	NS	NS	NS	23.9	1.0
Chen	2013	China	Supine	3D Conformal	Static tangents	Mixed energy MV photons	No	No	Breast	50	25	21	7.1	NS	NS	NS	25.1	9.4
			Prone	3D Conformal	Static tangents	Mixed energy MV photons	No	No	Breast	50	25	21	2.2	NS	NS	NS	3.1	1.2
			Prone	3D Conformal	Static tangents	Mixed energy MV photons	No	No	Breast	50	25	21	2.2	NS	NS	NS	3.1	1.2
			Prone	3D Conformal	Static tangents	Mixed energy MV photons	No	No	Breast	50	25	21	2.2	NS	NS	NS	3.1	1.2
			Supine	3D Conformal with FIF	Static tangents	Low energy MV photons	No	NS	Breast	50	25	21	6.0	NS	NS	NS	20.8	9.0
			Supine	3D Conformal with FIF	Static tangents	Low energy MV photons	No	NS	Breast	50	25	21	6.0	NS	NS	NS	20.8	9.0
			Supine	3D Conformal with FIF	Static tangents	Low energy MV photons	No	NS	Breast	50	25	21	6.0	NS	NS	NS	20.8	9.0
			Prone	3D Conformal with FIF	Static tangents	Low energy MV photons	No	NS	Breast	50	25	21	1.9	NS	NS	NS	3.0	1.1

Table E3 continued on the next page.

Table E3 continued from the previous page.

Author	Year	Country	Patient position	Treatment planning	Field type	Beam energy & modality	Breath. adapt.	RT plan delivered	Target(s)	Prescr. dose	No. of fraction	No. of CT plans	MLD$_{ipsi}$ (Gy) Avg	MLD$_{ipsi}$ (Gy) Range	MLD$_{cont}$ (Gy)	MLD$_{whole}$ (Gy)	V5$_{ipsi}$ (%)	V20$_{ipsi}$ (%)
			Description of regimens*										Lung dose measures					
			Prone	3D Conformal with FIF	Static tangents	Low energy MV photons	No	NS	Breast	50	25	21	1.9	NS	NS	NS	3.0	1.1
			Prone	3D Conformal with FIF	Static tangents	Low energy MV photons	No	NS	Breast	50	25	21	1.9	NS	NS	NS	3.0	1.1
Chi	2015	China	Supine	3D Conformal plan with FIF	Static tangents	Low energy MV phtons	No	NS	Breast	50	NS	31	8.0	NS	0.2	NS	NS	13.0
			Supine	3D Conformal plan with FIF	Static tangents	Low energy MV phtons	Yes	NS	Breast	50	NS	31	6.5	NS	0.2	NS	NS	9.2
			Supine	3D Conformal plan with FIF	Static tangents	Low energy MV phtons	Yes	NS	Breast	50	NS	31	6.5	NS	0.2	NS	NS	9.2
			Supine	3D Conformal plan with FIF	Static tangents	Low energy MV phtons	Yes	NS	Breast	50	NS	31	6.5	NS	0.2	NS	NS	9.2
			Supine	3D Conformal plan with FIF	Static tangents	Low energy MV phtons	Yes	NS	Breast	50	NS	31	7.0	NS	0.2	NS	NS	9.2
			Supine	3D Conformal plan with FIF	Static tangents	Low energy MV phtons	Yes	NS	Breast	50	NS	31	7.0	NS	0.2	NS	NS	9.2
			Supine	3D Conformal plan with FIF	Static tangents	Low energy MV phtons	Yes	NS	Breast	50	NS	31	7.0	NS	0.2	NS	NS	9.2
Chung M	2015	Korea	Supine	3D IMRT	Static tangents	Low energy MV phtons	No	Yes	Breast	50.4	28	26	5.1	NS	NS	NS	NS	9.0
			Supine	3D Conformal	Static tangents	Mixed energy MV photons	No	No	Breast	50.4	28	26	6.9	NS	NS	NS	NS	13.2
			Supine	3D Conformal	Static tangents	Mixed energy MV photons	No	No	Breast	50.4	28	26	6.9	NS	NS	NS	NS	13.2
			Supine	3D Conformal	Static tangents	Mixed energy MV photons	No	No	Breast	50.4	28	26	6.9	NS	NS	NS	NS	13.2
Chung Y	2015	Korea	Supine	3D Conformal	Tangents plus boost/direct fields	MV photons	No	No	Breast; SCF	NA	20-28	1	7.2	NS	NS	NS	NS	28.6
			Supine	3D Conformal	Wide static tangents	MV photons	No	No	Breast; IMC; SCF	NA	20-28	1	8.8	NS	NS	NS	NS	34.3
			Supine	3D Conformal	Wide static tangents	MV photons	No	No	Breast; IMC; SCF	NA	20-28	1	8.8	NS	NS	NS	NS	34.3
			Supine	3D Conformal	Wide static tangents	MV photons	No	No	Breast; IMC; SCF	NA	20-28	1	8.8	NS	NS	NS	NS	34.3
			Supine	3D Conformal	Tangents plus boost/direct fields	MV photons	No	No	Chest wall; SCF	NA	20-28	1	7.5	NS	NS	NS	NS	30.3
			Supine	3D Conformal	Tangents plus boost/direct fields	MV photons	No	No	Chest wall; SCF	NA	20-28	1	7.5	NS	NS	NS	NS	30.3
			Supine	3D Conformal	Tangents plus boost/direct fields	MV photons	No	No	Chest wall; SCF	NA	20-28	1	7.5	NS	NS	NS	NS	30.3
			Supine	3D Conformal	Wide static tangents	MV photons	No	No	Chest wall; SCF; IMC	NA	20-28	1	8.7	NS	NS	NS	NS	34.6
			Supine	3D Conformal	Wide static tangents	MV photons	No	No	Chest wall; SCF; IMC	NA	20-28	1	8.7	NS	NS	NS	NS	34.6
			Supine	3D Conformal	Wide static tangents	MV photons	No	No	Chest wall; SCF; IMC	NA	20-28	1	8.7	NS	NS	NS	NS	34.6
Cilla Digesu	2014	Italy	Supine	3D Conformal plan	Static tangents	Low energy MV photons	No	NS	Breast	50	25	15	10.0	NS	NS	NS	26.3	18.3
			Supine	3D Conformal plan with FIF	Static tangents	Low energy MV photons	No	NS	Breast	50	25	15	8.1	NS	NS	NS	23.0	14.5
			Supine	3D Conformal plan with FIF	Static tangents	Low energy MV photons	No	NS	Breast	50	25	15	8.1	NS	NS	NS	23.0	14.5
			Supine	3D Conformal plan with FIF	Static tangents	Low energy MV photons	No	NS	Breast	50	25	15	8.1	NS	NS	NS	23.0	14.5
			Supine	3D Conformal plan	Static tangents	Low energy MV photons	No	NS	Breast	50	25	15	9.6	NS	NS	NS	30.6	18.6
			Supine	3D Conformal plan	Static tangents	Low energy MV photons	No	NS	Breast	50	25	15	9.6	NS	NS	NS	30.6	18.6
			Supine	3D Conformal plan	Static tangents	Low energy MV photons	No	NS	Breast	50	25	15	9.6	NS	NS	NS	30.6	18.6
			Supine	3D Conformal plan with FIF	Static tangents	Low energy MV photons	No	NS	Breast	50	25	15	7.9	NS	NS	NS	26.7	14.7
			Supine	3D Conformal plan with FIF	Static tangents	Low energy MV photons	No	NS	Breast	50	25	15	7.9	NS	NS	NS	26.7	14.7
			Supine	3D Conformal plan with FIF	Static tangents	Low energy MV photons	No	NS	Breast	50	25	15	7.9	NS	NS	NS	26.7	14.7
Cilla Kigula	2013	Italy	Supine	3D Conformal	Static tangents	Low energy MV photons	No	Yes	Breast	50	25	14	5.1	NS	NS	NS	NS	8.5
			Supine	3D Conformal	Static tangents	Low energy MV photons	No	No	Breast	50	25	14	10.2	NS	NS	NS	NS	16.7

Table E3 continued on the next page.

Table E3 continued from the previous page.

Author	Year	Country	Description of regimens*									No. of CT plans	Lung dose measures					
			Patient position	Treatment planning	Field type	Beam energy & modality	Breath. adapt.	RT plan delivered	Target(s)	Prescr. dose	No. of fraction		MLD_{ipsi} (Gy)		MLD_{cont} (Gy)	MLD_{whole} (Gy)	$V5_{ipsi}$ (%)	$V20_{ipsi}$ (%)
													Avg	Range				
			Supine	3D Conformal	Static tangents	Low energy MV photons	No	No	Breast	50	25	14	10.2	NS	NS	NS	NS	16.7
			Supine	3D Conformal	Static tangents	Low energy MV photons	No	No	Breast	50	25	14	10.2	NS	NS	NS	NS	16.7
			Supine	3D Conformal	Static tangents	Low energy MV photons	No	No	Breast	50	25	14	12.1	NS	NS	NS	NS	18.5
			Supine	3D Conformal	Static tangents	Low energy MV photons	No	No	Breast	50	25	14	12.1	NS	NS	NS	NS	18.5
			Supine	3D Conformal	Static tangents	Low energy MV photons	No	No	Breast	50	25	14	12.1	NS	NS	NS	NS	18.5
			Supine	3D Conformal with FIF	Static tangents	Low energy MV photons	No	No	Breast	50	25	14	9.8	NS	NS	NS	NS	16.2
			Supine	3D Conformal with FIF	Static tangents	Low energy MV photons	No	No	Breast	50	25	14	9.8	NS	NS	NS	NS	16.2
			Supine	3D Conformal with FIF	Static tangents	Low energy MV photons	No	No	Breast	50	25	14	9.8	NS	NS	NS	NS	16.2
Comsa	2014	Canada	Supine	3D IMRT	Static tangents	Mixed energy MV photons	Yes	Yes	Breast or chest wall	NS	NS	20	5.5	NS	NS	2.6	22.0	11.0
			Supine	3D IMRT	Static tangents	Mixed energy MV photons	No	No	Breast or chest wall	NS	NS	20	5.3	NS	NS	2.5	19.0	11.0
			Supine	3D IMRT	Static tangents	Mixed energy MV photons	No	No	Breast or chest wall	NS	NS	20	5.3	NS	NS	2.5	19.0	11.0
			Supine	3D IMRT	Static tangents	Mixed energy MV photons	No	No	Breast or chest wall	NS	NS	20	5.3	NS	NS	2.5	19.0	11.0
			Supine	3D IMRT	Tangents plus boost/direct fields	Mixed energy MV photons	Yes	Yes	Breast or chest wall; SCF; axilla	NS	NS	30	11.2	NS	NS	5.4	40.0	22.0
			Supine	3D IMRT	Tangents plus boost/direct fields	Mixed energy MV photons	Yes	Yes	Breast or chest wall; SCF; axilla	NS	NS	30	11.2	NS	NS	5.4	40.0	22.0
			Supine	3D IMRT	Tangents plus boost/direct fields	Mixed energy MV photons	Yes	Yes	Breast or chest wall; SCF; axilla	NS	NS	30	11.2	NS	NS	5.4	40.0	22.0
			Supine	3D IMRT	Tangents plus boost/direct fields	Mixed energy MV photons	No	No	Breast or chest wall; SCF; axilla	NS	NS	30	14.3	NS	NS	6.8	45.0	28.0
			Supine	3D IMRT	Tangents plus boost/direct fields	Mixed energy MV photons	No	No	Breast or chest wall; SCF; axilla	NS	NS	30	14.3	NS	NS	6.8	45.0	28.0
			Supine	3D IMRT	Tangents plus boost/direct fields	Mixed energy MV photons	No	No	Breast or chest wall; SCF; axilla	NS	NS	30	14.3	NS	NS	6.8	45.0	28.0
Cuaron	2015	USA	Supine	Uniform scanning	Direct fields	Protons	No	Yes	Breast or chest wall	50.4	25	30	NS	NS	NS	NS	34.4	16.5
Dinclogan	2013	Turkey	Supine	3D Conformal	Static tangents plus electron field	NS	No	No	Chest wall; SCF; axilla; IMC	50	25	27	18.8	13.2-28.4	NS	9.1	NS	32.4
			Supine	3D Conformal	Static tangents plus electron field	NS	Yes	Yes	Chest wall; SCF; axilla; IMC	50	25	27	15.1	10.2-22.8	NS	7.1	NS	27.2
			Supine	3D Conformal	Static tangents plus electron field	NS	Yes	Yes	Chest wall; SCF; axilla; IMC	50	25	27	15.1	10.2-22.8	NS	7.1	NS	27.2
			Supine	3D Conformal	Static tangents plus electron field	NS	Yes	Yes	Chest wall; SCF; axilla; IMC	50	25	27	15.1	10.2-22.8	NS	7.1	NS	27.2
Dogan	2013	Turkey	Supine	3D Conformal	Wide static tangents	Mixed photons & electrons	No	NS	Breast; SCF; IMC	45	25	12	6.3	NS	NS	NS	NS	NS
			Supine	3D Conformal	Static tangents plus electron field	Mixed photons & electrons	No	NS	Breast; SCF; IMC	45	25	12	8.9	NS	NS	NS	NS	NS
			Supine	3D Conformal	Static tangents plus electron field	Mixed photons & electrons	No	NS	Breast; SCF; IMC	45	25	12	8.9	NS	NS	NS	NS	NS
			Supine	3D Conformal	Static tangents plus electron field	Mixed photons & electrons	No	NS	Breast; SCF; IMC	45	25	12	8.9	NS	NS	NS	NS	NS
			Supine	3D Conformal	Static tangents plus electron field	Mixed photons & electrons	No	NS	Breast; SCF; IMC	45	25	12	3.0	NS	NS	NS	NS	NS
			Supine	3D Conformal	Static tangents plus electron field	Mixed photons & electrons	No	NS	Breast; SCF; IMC	45	25	12	3.0	NS	NS	NS	NS	NS
			Supine	3D Conformal	Static tangents plus electron field	Mixed photons & electrons	No	NS	Breast; SCF; IMC	45	25	12	3.0	NS	NS	NS	NS	NS

Table E3 continued on the next page.

Table E3 continued from the previous page.

Author	Year	Country	Description of regimens*									No. of CT plans	Lung dose measures					
			Patient position	Treatment planning	Field type	Beam energy & modality	Breath. adapt.	RT plan delivered	Target(s)	Prescr. dose	No. of fraction		MLD_{ipsi} (Gy) Avg	MLD_{ipsi} (Gy) Range	MLD_{cont} (Gy)	MLD_{whole} (Gy)	$V5_{ipsi}$ (%)	$V20_{ipsi}$ (%)
			Supine	3D Conformal	Wide static tangents plus electron field	Mixed photons & electrons	No	NS	Breast; SCF; IMC	45	25	12	3.3	NS	NS	NS	NS	NS
			Supine	3D Conformal	Wide static tangents plus electron field	Mixed photons & electrons	No	NS	Breast; SCF; IMC	45	25	12	3.3	NS	NS	NS	NS	NS
			Supine	3D Conformal	Wide static tangents plus electron field	Mixed photons & electrons	No	NS	Breast; SCF; IMC	45	25	12	3.3	NS	NS	NS	NS	NS
			Supine	3D Conformal	Static tangents plus electron field	Mixed photons & electrons	No	NS	Breast; SCF	45	25	12	5.4	NS	NS	NS	NS	NS
			Supine	3D Conformal	Static tangents plus electron field	Mixed photons & electrons	No	NS	Breast; SCF	45	25	12	5.4	NS	NS	NS	NS	NS
			Supine	3D Conformal	Static tangents plus electron field	Mixed photons & electrons	No	NS	Breast; SCF	45	25	12	5.4	NS	NS	NS	NS	NS
Donovan	2014	UK	Supine	3D Conformal with FIF	Static tangents	MV photons	No	Yes	Breast	40-44	15	60	5.6	2.6-11.3	NS	NS	NS	9.6
			Supine	3D Conformal with FIF	Static tangents	MV photons	No	No	Breast	40-44	15	60	6.1	2.8-11.5	NS	NS	NS	10.0
			Supine	3D Conformal with FIF	Static tangents	MV photons	No	No	Breast	40-44	15	60	6.1	2.8-11.5	NS	NS	NS	10.0
			Supine	3D Conformal with FIF	Static tangents	MV photons	No	No	Breast	40-44	15	60	6.1	2.8-11.5	NS	NS	NS	10.0
Ducournau	2014	France	Supine	3D Conformal with FIF	Tangents plus boost/direct fields	Low energy MV photons	No	Yes	Breast	50	25	1	NS	NS	NS	NS	NS	3.2
			Supine	3D Conformal with FIF	Tangents plus boost/direct fields	Low energy MV photons	No	Yes	Breast	50	25	1	NS	NS	NS	NS	NS	0.0
			Supine	3D Conformal with FIF	Tangents plus boost/direct fields	Low energy MV photons	No	Yes	Breast	50	25	1	NS	NS	NS	NS	NS	0.0
			Supine	3D Conformal with FIF	Tangents plus boost/direct fields	Low energy MV photons	No	Yes	Breast	50	25	1	NS	NS	NS	NS	NS	0.0
Edvardsson	2015	Sweden	Supine	3D Conformal with FIF	Static tangents	Mixed energy MV photons	No	NS	Breast	50	25	16	5.4	2.1-9.4	NS	NS	NS	9.1
			Supine	3D Conformal with FIF	Static tangents	Mixed energy MV photons	Yes	NS	Breast	50	25	16	5.5	2.6-9.1	NS	NS	NS	9.1
			Supine	3D Conformal with FIF	Static tangents	Mixed energy MV photons	Yes	NS	Breast	50	25	16	5.5	2.6-9.1	NS	NS	NS	9.1
			Supine	3D Conformal with FIF	Static tangents	Mixed energy MV photons	Yes	NS	Breast	50	25	16	5.5	2.6-9.1	NS	NS	NS	9.1
			Supine	3D Conformal with FIF	Static tangents	Mixed energy MV photons	No	NS	Breast or chest wall; SCF; axilla	50	25	16	14.0	6.4-18.9	NS	NS	NS	27.0
			Supine	3D Conformal with FIF	Static tangents	Mixed energy MV photons	No	NS	Breast or chest wall; SCF; axilla	50	25	16	14.0	6.4-18.9	NS	NS	NS	27.0
			Supine	3D Conformal with FIF	Static tangents	Mixed energy MV photons	No	NS	Breast or chest wall; SCF; axilla	50	25	16	14.0	6.4-18.9	NS	NS	NS	27.0
			Supine	3D Conformal with FIF	Static tangents	Mixed energy MV photons	Yes	NS	Breast or chest wall; SCF; axilla	50	25	16	11.2	6.1-16.4	NS	NS	NS	21.5
			Supine	3D Conformal with FIF	Static tangents	Mixed energy MV photons	Yes	NS	Breast or chest wall; SCF; axilla	50	25	16	11.2	6.1-16.4	NS	NS	NS	21.5
			Supine	3D Conformal with FIF	Static tangents	Mixed energy MV photons	Yes	NS	Breast or chest wall; SCF; axilla	50	25	16	11.2	6.1-16.4	NS	NS	NS	21.5
Ekambaram	2015	India	Supine	3D IMRT	Oblique fields	Low energy MV photons	No	No	Chest wall; SCF; axilla	50	25	20	14.3	NS	5.5	NS	NS	20.1
			Supine	3D IMRT	Rotational fields	Low energy MV photons	No	No	Chest wall; SCF; axilla	50	25	20	12.1	NS	5.0	NS	NS	18.4

Table E3 continued on the next page.

Table E3 continued from the previous page.

Author	Year	Country	Patient position	Treatment planning	Field type	Beam energy & modality	Breath. adapt.	RT plan delivered	Target(s)	Prescr. dose	No. of fraction	No. of CT plans	MLD_{ipsi} (Gy) Avg	MLD_{ipsi} (Gy) Range	MLD_{cont} (Gy)	MLD_{whole} (Gy)	$V5_{ipsi}$ (%)	$V20_{ipsi}$ (%)
			*Description of regimens**										*Lung dose measures*					
			Supine	3D IMRT	Rotational fields	Low energy MV photons	No	No	Chest wall; SCF; axilla	50	25	20	12.1	NS	5.0	NS	NS	18.4
			Supine	3D IMRT	Rotational fields	Low energy MV photons	No	No	Chest wall; SCF; axilla	50	25	20	12.1	NS	5.0	NS	NS	18.4
Eldredge	2015	USA	Supine	3D Conformal	Tangents plus boost/direct fields	Mixed energy MV photons	Yes	Yes	Breast or chest wall	42-50	16-28	86	5.9	5.4-6.3	NS	NS	20.9	10.4
			Supine	3D Conformal	Tangents plus boost/direct fields	Mixed energy MV photons	No	No	Breast or chest wall	42-50	16-28	86	6.4	5.7-7.0	NS	NS	22.9	12.0
			Supine	3D Conformal	Tangents plus boost/direct fields	Mixed energy MV photons	No	No	Breast or chest wall	42-50	16-28	86	6.4	5.7-7.0	NS	NS	22.9	12.0
			Supine	3D Conformal	Tangents plus boost/direct fields	Mixed energy MV photons	No	No	Breast or chest wall	42-50	16-28	86	6.4	5.7-7.0	NS	NS	22.9	12.0
Erven	2011	Belgium	Supine	3D Conformal	Wide static tangents	MV photons	No	No	Breast; IMC	50	25	20	NS	NS	NS	8.8	NS	NS
			Supine	3D Conformal	Static tangents	MV photons	No	No	Breast; IMC	50	25	20	NS	NS	NS	9.1	NS	NS
			Supine	3D Conformal	Static tangents	MV photons	No	No	Breast; IMC	50	25	20	NS	NS	NS	9.1	NS	NS
			Supine	3D Conformal	Static tangents	MV photons	No	No	Breast; IMC	50	25	20	NS	NS	NS	9.1	NS	NS
Essers	2014	Netherlands	Supine	3D Conformal	Oblique fields	Mixed energy MV photons	No	No	Partial breast	38.5	10	21	3.7	0.7-8.7	0.1	NS	31.2	NS
			Supine	3D Conformal	Oblique fields	Mixed energy MV photons	Yes	No	Partial breast	38.5	10	21	3.4	1.2-8.0	0.1	NS	27.4	NS
			Supine	3D Conformal	Oblique fields	Mixed energy MV photons	Yes	No	Partial breast	38.5	10	21	3.4	1.2-8.0	0.1	NS	27.4	NS
			Supine	3D Conformal	Oblique fields	Mixed energy MV photons	Yes	No	Partial breast	38.5	10	21	3.4	1.2-8.0	0.1	NS	27.4	NS
			Supine	3D IMRT	Rotational fields	Low energy MV photons	No	No	Partial breast	38.5	10	21	1.9	0.4-5.5	0.4	NS	10.4	NS
			Supine	3D IMRT	Rotational fields	Low energy MV photons	No	No	Partial breast	38.5	10	21	1.9	0.4-5.5	0.4	NS	10.4	NS
			Supine	3D IMRT	Rotational fields	Low energy MV photons	No	No	Partial breast	38.5	10	21	1.9	0.4-5.5	0.4	NS	10.4	NS
			Supine	3D IMRT	Rotational fields	Low energy MV photons	Yes	No	Partial breast	38.5	10	21	1.8	0.5-4.0	0.4	NS	10.6	NS
			Supine	3D IMRT	Rotational fields	Low energy MV photons	Yes	No	Partial breast	38.5	10	21	1.8	0.5-4.0	0.4	NS	10.6	NS
			Supine	3D IMRT	Rotational fields	Low energy MV photons	Yes	No	Partial breast	38.5	10	21	1.8	0.5-4.0	0.4	NS	10.6	NS
Fan	2014	China	Supine	3D IMRT	Oblique fields	Low energy MV photons	No	NS	Breast	50	25	10	14.1	NS	2.6	NS	85.7	20.9
			Prone	3D IMRT	Oblique fields	Low energy MV photons	No	NS	Breast	50	25	10	8.6	NS	2.2	NS	57.8	10.2
			Prone	3D IMRT	Oblique fields	Low energy MV photons	No	NS	Breast	50	25	10	8.6	NS	2.2	NS	57.8	10.2
			Prone	3D IMRT	Oblique fields	Low energy MV photons	No	NS	Breast	50	25	10	8.6	NS	2.2	NS	57.8	10.2
Farace	2012	Italy	Supine	3D IMRT	Static tangents	Low energy MV photons	No	Yes	Breast	50	25	34	NS	NS	NS	12.5	NS	NS
			Supine	3D IMRT	Static tangents	Low energy MV photons	No	Yes	Breast	50	25	8	NS	NS	NS	10.6	NS	NS
			Supine	3D IMRT	Static tangents	Low energy MV photons	No	Yes	Breast	50	25	8	NS	NS	NS	10.6	NS	NS
			Supine	3D IMRT	Static tangents	Low energy MV photons	No	Yes	Breast	50	25	8	NS	NS	NS	10.6	NS	NS
			Supine	3D IMRT	Static tangents	Low energy MV photons	No	Yes	Breast	50	25	27	NS	NS	NS	7.1	NS	NS
			Supine	3D IMRT	Static tangents	Low energy MV photons	No	Yes	Breast	50	25	27	NS	NS	NS	7.1	NS	NS
			Supine	3D IMRT	Static tangents	Low energy MV photons	No	Yes	Breast	50	25	27	NS	NS	NS	7.1	NS	NS
			Supine	3D IMRT	Static tangents	Low energy MV photons	No	Yes	Breast	50	25	9	NS	NS	NS	6.2	NS	NS
			Supine	3D IMRT	Static tangents	Low energy MV photons	No	Yes	Breast	50	25	9	NS	NS	NS	6.2	NS	NS
			Supine	3D IMRT	Static tangents	Low energy MV photons	No	Yes	Breast	50	25	9	NS	NS	NS	6.2	NS	NS
Fekete	2015	Hungary	Supine	3D Conformal	Oblique fields	Mixed energy MV photons	No	NS	Partial breast	39-50	13-25	72	NS	NS	NS	NS	NS	3.3
			Supine	3D Conformal	Oblique fields	Electrons	No	NS	Partial breast	39-50	13-25	72	NS	NS	NS	NS	NS	4.3

Table E3 continued on the next page.

Table E3 continued from the previous page.

Author	Year	Country	Description of regimens* Patient position	Treatment planning	Field type	Beam energy & modality	Breath. adapt.	RT plan delivered	Target(s)	Prescr. dose	No. of fraction	No. of CT plans	Lung dose measures MLD$_{ipsi}$ (Gy) Avg	Range	MLD$_{cont}$ (Gy)	MLD$_{whole}$ (Gy)	V5$_{ipsi}$ (%)	V20$_{ipsi}$ (%)
			Supine	3D Conformal	Oblique fields	Electrons	No	NS	Partial breast	39-50	13-25	72	NS	NS	NS	NS	NS	4.3
			Supine	3D Conformal	Oblique fields	Electrons	No	NS	Partial breast	39-50	13-25	72	NS	NS	NS	NS	NS	4.3
Fernandez-Lizarbe	2013	Spain	Supine	3D Conformal	Static tangents	Mixed energy MV photons	No	NS	Breast	50	25	6	13.8	7.5-24	NS	NS	NS	NS
			Prone	3D Conformal	Static tangents	Mixed energy MV photons	No	NS	Breast	50	25	6	3.0	1-9.7	NS	NS	NS	NS
			Prone	3D Conformal	Static tangents	Mixed energy MV photons	No	NS	Breast	50	25	6	3.0	1-9.7	NS	NS	NS	NS
			Prone	3D Conformal	Static tangents	Mixed energy MV photons	No	NS	Breast	50	25	6	3.0	1-9.7	NS	NS	NS	NS
			Supine	3D Conformal	Static tangents	Mixed energy MV photons	No	NS	Breast	50	25	4	13.3	11-17.3	NS	NS	NS	NS
			Supine	3D Conformal	Static tangents	Mixed energy MV photons	No	NS	Breast	50	25	4	13.3	11-17.3	NS	NS	NS	NS
			Supine	3D Conformal	Static tangents	Mixed energy MV photons	No	NS	Breast	50	25	4	13.3	11-17.3	NS	NS	NS	NS
			Prone	3D Conformal	Static tangents	Mixed energy MV photons	No	NS	Breast	50	25	4	3.2	2.4-4	NS	NS	NS	NS
			Prone	3D Conformal	Static tangents	Mixed energy MV photons	No	NS	Breast	50	25	4	3.2	2.4-4	NS	NS	NS	NS
			Prone	3D Conformal	Static tangents	Mixed energy MV photons	No	NS	Breast	50	25	4	3.2	2.4-4	NS	NS	NS	NS
Fjelmer D	2015	Sweden	Supine	3D Conformal	Static tangents	MV photons	No	Yes	Breast; SCF	50	25	20	19.7	NS	NS	NS	NS	17.9
			Supine	3D Conformal	Static tangents	MV photons	No	No	Breast; SCF	50	25	20	20.5	NS	NS	NS	NS	18.6
			Supine	3D Conformal	Static tangents	MV photons	No	No	Breast; SCF	50	25	20	20.5	NS	NS	NS	NS	18.6
			Supine	3D Conformal	Static tangents	MV photons	No	No	Breast; SCF	50	25	20	20.5	NS	NS	NS	NS	18.6
			Supine	3D Conformal	Static tangents	MV photons	No	Yes	Breast	50	25	20	8.3	NS	NS	NS	NS	6.3
			Supine	3D Conformal	Static tangents	MV photons	No	Yes	Breast	50	25	20	8.3	NS	NS	NS	NS	6.3
			Supine	3D Conformal	Static tangents	MV photons	No	Yes	Breast	50	25	20	8.3	NS	NS	NS	NS	6.3
			Supine	3D Conformal	Static tangents	MV photons	No	No	Breast	50	25	20	8.9	NS	NS	NS	NS	6.6
			Supine	3D Conformal	Static tangents	MV photons	No	No	Breast	50	25	20	8.9	NS	NS	NS	NS	6.6
			Supine	3D Conformal	Static tangents	MV photons	No	No	Breast	50	25	20	8.9	NS	NS	NS	NS	6.6
Fjelmer J	2014	Sweden	Supine	3D Conformal	Static tangents	MV photons	No	No	Breast; +/- SCF	50	25	10	16.1	NS	0.3	NS	NS	13.8
			Supine	3D IMRT	Static tangents	MV photons	No	No	Breast; +/- SCF	50	25	10	15.3	NS	0.3	NS	NS	12.8
			Supine	3D IMRT	Static tangents	MV photons	No	No	Breast; +/- SCF	50	25	10	15.3	NS	0.3	NS	NS	12.8
			Supine	3D IMRT	Static tangents	MV photons	No	No	Breast; +/- SCF	50	25	10	15.3	NS	0.3	NS	NS	12.8
Franco	2013	Italy	Supine	3D IMRT	Rotational fields	MV photons	No	Yes	Breast	50	25	120	6.0	NS	NS	NS	20.2	9.7
			Supine	3D IMRT	Rotational fields	MV photons	No	Yes	Breast	50	25	120	6.5	NS	NS	NS	22.4	10.8
			Supine	3D IMRT	Rotational fields	MV photons	No	Yes	Breast	50	25	120	6.5	NS	NS	NS	22.4	10.8
			Supine	3D IMRT	Rotational fields	MV photons	No	Yes	Breast	50	25	120	6.5	NS	NS	NS	22.4	10.8
Gauer	2010	Germany	Supine	3D Conformal	Static tangents	Low energy MV photons	No	NS	Breast	50.4	28	1	9.8	NS	0.2	NS	25.3	18.7
			Supine	3D Conformal	Static tangents plus electron field	Mixed photons & electrons	No	NS	Chest wall; IMC	50.4	28	1	9.0	NS	1.0	NS	27.7	14.3
			Supine	3D Conformal	Static tangents plus electron field	Mixed photons & electrons	No	NS	Chest wall; IMC	50.4	28	1	9.0	NS	1.0	NS	27.7	14.3
			Supine	3D Conformal	Static tangents plus electron field	Mixed photons & electrons	No	NS	Chest wall; IMC	50.4	28	1	9.0	NS	1.0	NS	27.7	14.3
			Supine	3D IMRT	Rotational fields	Low energy MV photons	No	NS	Breast	50.4	28	1	7.5	NS	4.8	NS	38.2	10.1
			Supine	3D IMRT	Rotational fields	Low energy MV photons	No	NS	Breast	50.4	28	1	7.5	NS	4.8	NS	38.2	10.1
			Supine	3D IMRT	Rotational fields	Low energy MV photons	No	NS	Breast	50.4	28	1	7.5	NS	4.8	NS	38.2	10.1
			Supine	3D IMRT	Rotational fields	Low energy MV photons	No	NS	Chest wall; IMC	50.4	28	1	8.2	NS	6.5	NS	54.4	10.1
			Supine	3D IMRT	Rotational fields	Low energy MV photons	No	NS	Chest wall; IMC	50.4	28	1	8.2	NS	6.5	NS	54.4	10.1
			Supine	3D IMRT	Rotational fields	Low energy MV photons	No	NS	Chest wall; IMC	50.4	28	1	8.2	NS	6.5	NS	54.4	10.1
			Supine	3D IMRT	Direct fields	Electrons	No	NS	Breast	50.4	28	1	9.1	NS	0.7	NS	41.8	18.5

Table E3 continued on the next page.

Table E3 continued from the previous page.

Author	Year	Country	Description of regimens*									No. of CT plans	Lung dose measures					
			Patient position	Treatment planning	Field type	Beam energy & modality	Breath. adapt.	RT plan delivered	Target(s)	Prescr. dose	No. of fraction		MLD_{ipsi} (Gy)		MLD_{cont} (Gy)	MLD_{whole} (Gy)	$V5_{ipsi}$ (%)	$V20_{ipsi}$ (%)
													Avg	Range				
			Supine	3D IMRT	Direct fields	Electrons	No	NS	Breast	50.4	28	1	9.1	NS	0.7	NS	41.8	18.5
			Supine	3D IMRT	Direct fields	Electrons	No	NS	Breast	50.4	28	1	9.1	NS	0.7	NS	41.8	18.5
			Supine	3D IMRT	Direct fields	Electrons	No	NS	Chest wall; IMC	50.4	28	1	10.9	NS	15.0	NS	51.6	19.9
			Supine	3D IMRT	Direct fields	Electrons	No	NS	Chest wall; IMC	50.4	28	1	10.9	NS	15.0	NS	51.6	19.9
			Supine	3D IMRT	Direct fields	Electrons	No	NS	Chest wall; IMC	50.4	28	1	10.9	NS	15.0	NS	51.6	19.9
Guenzi	2015	Italy	Supine	3D Conformal with FIF	Static tangents	Mixed energy MV photons	No	Yes	Breast	35	10	12	6.6	1.9-13.3	NS	NS	NS	NS
			Prone	3D Conformal with FIF	Static tangents	Mixed energy MV photons	No	Yes	Breast	35	10	12	0.8	0.4-1.8	NS	NS	NS	NS
			Prone	3D Conformal with FIF	Static tangents	Mixed energy MV photons	No	Yes	Breast	35	10	12	0.8	0.4-1.8	NS	NS	NS	NS
			Prone	3D Conformal with FIF	Static tangents	Mixed energy MV photons	No	Yes	Breast	35	10	12	0.8	0.4-1.8	NS	NS	NS	NS
Giraud	2012	France	Supine	NS	NS	Low energy MV photons	No	Yes	NS	46	21-26	154	NS	NS	NS	4.4	NS	8.5
			Supine	NS	NS	Low energy MV photons	Yes	Yes	NS	46	21-26	79	NS	NS	NS	3.9	NS	6.8
			Supine	NS	NS	Low energy MV photons	Yes	Yes	NS	46	21-26	79	NS	NS	NS	3.9	NS	6.8
			Supine	NS	NS	Low energy MV photons	Yes	Yes	NS	46	21-26	79	NS	NS	NS	3.9	NS	6.8
Guilbert	2012	France	Supine	3D Conformal plan	Tangents plus boost/direct fields	Mixed photons electrons	No	No	Breast; SCF; infra-clav; IMC	50	25	20	NS	NS	NS	NS	NS	18.9
			Supine	3D Conformal plan	Tangents plus boost/direct fields	Mixed photons electrons	No	Yes	Breast; SCF; infra-clav; IMC	50	25	20	NS	NS	NS	NS	NS	20.0
			Supine	3D Conformal plan	Tangents plus boost/direct fields	Mixed photons electrons	No	Yes	Breast; SCF; infra-clav; IMC	50	25	20	NS	NS	NS	NS	NS	20.0
			Supine	3D Conformal plan	Tangents plus boost/direct fields	Mixed photons electrons	No	Yes	Breast; SCF; infra-clav; IMC	50	25	20	NS	NS	NS	NS	NS	20.0
Gultekin	2014	Turkey	Supine	3D Conformal	Wide static tangents	MV photons	No	No	Chest wall; SCF; axilla; IMC	50	25	10	18.3	NS	1.0	9.2	NS	NS
			Supine	3D Conformal	Static tangents plus electron field	Mixed photons & electrons	No	No	Chest wall; SCF; axilla; IMC	50	25	10	22.0	NS	0.9	11.1	NS	NS
			Supine	3D Conformal	Static tangents plus electron field	Mixed photons & electrons	No	No	Chest wall; SCF; axilla; IMC	50	25	10	22.0	NS	0.9	11.1	NS	NS
			Supine	3D Conformal	Static tangents plus electron field	Mixed photons & electrons	No	No	Chest wall; SCF; axilla; IMC	50	25	10	22.0	NS	0.9	11.1	NS	NS
			Supine	3D Conformal	Static tangents plus electron field	Mixed photons & electrons	No	No	Chest wall; SCF; axilla; IMC	50	25	10	19.9	NS	0.9	10.2	NS	NS
			Supine	3D Conformal	Static tangents plus electron field	Mixed photons & electrons	No	No	Chest wall; SCF; axilla; IMC	50	25	10	19.9	NS	0.9	10.2	NS	NS
			Supine	3D Conformal	Static tangents plus electron field	Mixed photons & electrons	No	No	Chest wall; SCF; axilla; IMC	50	25	10	19.9	NS	0.9	10.2	NS	NS
			Supine	3D Conformal	Direct fields	Electrons	No	No	Chest wall; SCF; axilla; IMC	50	25	10	23.7	NS	1.3	12.1	NS	NS
			Supine	3D Conformal	Direct fields	Electrons	No	No	Chest wall; SCF; axilla; IMC	50	25	10	23.7	NS	1.3	12.1	NS	NS
			Supine	3D Conformal	Direct fields	Electrons	No	No	Chest wall; SCF; axilla; IMC	50	25	10	23.7	NS	1.3	12.1	NS	NS
Gursel	2011	Turkey	Supine	2D Conformal	Static tangents	MV photons	No	No	Breast	50	25	30	7.8	NS	NS	NS	19.2	14.1
			Supine	3D Conformal	Static tangents	MV photons	No	Yes	Breast	50	25	30	8.3	NS	NS	NS	19.9	14.8
			Supine	3D Conformal	Static tangents	MV photons	No	Yes	Breast	50	25	30	8.3	NS	NS	NS	19.9	14.8
			Supine	3D Conformal	Static tangents	MV photons	No	Yes	Breast	50	25	30	8.3	NS	NS	NS	19.9	14.8

Table E3 continued on the next page.

Table E3 continued from the previous page.

Author	Year	Country	Description of regimens*									No. of CT plans	Lung dose measures					
			Patient position	Treatment planning	Field type	Beam energy & modality	Breath. adapt.	RT plan delivered	Target(s)	Prescr. dose	No. of fraction		MLD_{ipsi} (Gy)		MLD_{cont} (Gy)	MLD_{whole} (Gy)	$V5_{ipsi}$ (%)	$V20_{ipsi}$ (%)
													Avg	Range				
			Supine	3D Conformal with FIF	Static tangents	MV photons	No	No	Breast	50	25	30	7.8	NS	NS	NS	19.5	13.9
			Supine	3D Conformal with FIF	Static tangents	MV photons	No	No	Breast	50	25	30	7.8	NS	NS	NS	19.5	13.9
			Supine	3D Conformal with FIF	Static tangents	MV photons	No	No	Breast	50	25	30	7.8	NS	NS	NS	19.5	13.9
Haciislamoglu	2015	Turkey	Supine	3D Conformal	Static tangents	Low energy MV photons	No	No	Breast	50	25	15	7.2	NS	0.4	NS	18.2	12.5
			Supine	3D Conformal with FIF	Static tangents	Low energy MV photons	No	No	Breast	50	25	15	7.1	NS	0.4	NS	12.5	12.5
			Supine	3D Conformal with FIF	Static tangents	Low energy MV photons	No	No	Breast	50	25	15	7.1	NS	0.4	NS	12.5	12.5
			Supine	3D Conformal with FIF	Static tangents	Low energy MV photons	No	No	Breast	50	25	15	7.1	NS	0.4	NS	12.5	12.5
			Supine	3D IMRT	Oblique fields	Low energy MV photons	No	No	Breast	50	25	15	12.7	NS	4.3	NS	85.8	14.9
			Supine	3D IMRT	Oblique fields	Low energy MV photons	No	No	Breast	50	25	15	12.7	NS	4.3	NS	85.8	14.9
			Supine	3D IMRT	Oblique fields	Low energy MV photons	No	No	Breast	50	25	15	12.7	NS	4.3	NS	85.8	14.9
			Supine	3D IMRT	Rotational fields	Low energy MV photons	No	No	Breast	50	25	15	6.2	NS	3.5	NS	57.0	7.6
			Supine	3D IMRT	Rotational fields	Low energy MV photons	No	No	Breast	50	25	15	6.2	NS	3.5	NS	57.0	7.6
			Supine	3D IMRT	Rotational fields	Low energy MV photons	No	No	Breast	50	25	15	6.2	NS	3.5	NS	57.0	7.6
			Supine	3D IMRT	Rotational fields	Low energy MV photons	No	No	Breast	50	25	15	11.1	NS	3.0	NS	73.7	13.7
			Supine	3D IMRT	Rotational fields	Low energy MV photons	No	No	Breast	50	25	15	11.1	NS	3.0	NS	73.7	13.7
			Supine	3D IMRT	Rotational fields	Low energy MV photons	No	No	Breast	50	25	15	11.1	NS	3.0	NS	73.7	13.7
Hashimoto	2015	Japan	Supine	3D IMRT	Static tangents	Low energy MV photons	No	Yes	Breast	50	25	45	8.2	NS	NS	NS	NS	16.1
			Supine	3D IMRT	Oblique fields	Low energy MV photons	No	Yes	Breast	50	25	45	7.7	NS	NS	NS	NS	13.4
			Supine	3D IMRT	Oblique fields	Low energy MV photons	No	Yes	Breast	50	25	45	7.7	NS	NS	NS	NS	13.4
			Supine	3D IMRT	Oblique fields	Low energy MV photons	No	Yes	Breast	50	25	45	7.7	NS	NS	NS	NS	13.4
Hayden	2012	Australia	Supine	3D IMRT	Static tangents	MV photons	Yes	No	Breast	50	25	30	12.8	NS	NS	NS	NS	22.1
			Supine	3D IMRT	Static tangents	MV photons	No	No	Breast	50	25	30	13.4	NS	NS	NS	NS	23.2
			Supine	3D IMRT	Static tangents	MV photons	No	No	Breast	50	25	30	13.4	NS	NS	NS	NS	23.2
			Supine	3D IMRT	Static tangents	MV photons	No	No	Breast	50	25	30	13.4	NS	NS	NS	NS	23.2
He Wu	2014	China	Supine	3D IMRT	Oblique fields	Low energy MV photons	No	Yes	Partial breast	34	10	32	3.2	NS	0.2	NS	46.6	11.8
He Chi	2011	China	Supine	3D Conformal plan with FIF	Static tangents	MV photons	No	NS	Breast	50	25	18	8.0	NS	NS	3.6	NS	13.1
			Supine	3D Conformal plan with FIF	Static tangents	MV photons	Yes	NS	Breast	50	25	18	6.5	NS	NS	2.9	23.6	9.8
			Supine	3D Conformal plan with FIF	Static tangents	MV photons	Yes	NS	Breast	50	25	18	6.5	NS	NS	2.9	23.6	9.8
			Supine	3D Conformal plan with FIF	Static tangents	MV photons	Yes	NS	Breast	50	25	18	6.5	NS	NS	2.9	23.6	9.8
			Supine	3D Conformal plan with FIF	Static tangents	MV photons	No	NS	Breast	50	25	18	8.0	NS	NS	3.6	26.0	13.1
			Supine	3D Conformal plan with FIF	Static tangents	MV photons	No	NS	Breast	50	25	18	8.0	NS	NS	3.6	26.0	13.1
			Supine	3D Conformal plan with FIF	Static tangents	MV photons	No	NS	Breast	50	25	18	8.0	NS	NS	3.6	26.0	13.1
Henzen	2014	Switzerland	Supine	3D Conformal	Static tangents	Low energy MV photons	No	No	Breast	50	25	1	6.8	NS	NS	NS	NS	12.0
			Supine	3D IMRT	Oblique fields	Electrons	No	No	Breast	50	25	1	6.4	NS	NS	NS	NS	5.0
			Supine	3D IMRT	Oblique fields	Electrons	No	No	Breast	50	25	1	6.4	NS	NS	NS	NS	5.0
			Supine	3D IMRT	Oblique fields	Electrons	No	No	Breast	50	25	1	6.4	NS	NS	NS	NS	5.0
			Supine	3D Conformal	Static tangents	Low energy MV photons	No	No	Chest wall	50	25	1	13.1	NS	NS	NS	NS	12.0
			Supine	3D Conformal	Static tangents	Low energy MV photons	No	No	Chest wall	50	25	1	13.1	NS	NS	NS	NS	12.0
			Supine	3D Conformal	Static tangents	Low energy MV photons	No	No	Chest wall	50	25	1	13.1	NS	NS	NS	NS	12.0
			Supine	3D IMRT	Oblique fields	Electrons	No	No	Chest wall	50	25	1	11.2	NS	NS	NS	NS	8.0
			Supine	3D IMRT	Oblique fields	Electrons	No	No	Chest wall	50	25	1	11.2	NS	NS	NS	NS	8.0
			Supine	3D IMRT	Oblique fields	Electrons	No	No	Chest wall	50	25	1	11.2	NS	NS	NS	NS	8.0

Table E3 continued on the next page.

Table E3 continued from the previous page.

Author	Year	Country	Description of regimens*									No. of CT plans	Lung dose measures					
			Patient position	Treatment planning	Field type	Beam energy & modality	Breath. adapt.	RT plan delivered	Target(s)	Prescr. dose	No. of fraction		MLD_{ipsi} (Gy)		MLD_{cont} (Gy)	MLD_{whole} (Gy)	$V5_{ipsi}$ (%)	$V20_{ipsi}$ (%)
													Avg	Range				
Hepp	2015	Germany	Supine	3D Conformal with FIF	Static tangents	Low energy MV photons	Yes	Yes	Breast	50	25	20	6.3	NS	NS	NS	23.0	12.0
			Supine	3D Conformal with FIF	Static tangents	Low energy MV photons	No	No	Breast	50	25	20	8.7	NS	NS	NS	27.0	17.0
			Supine	3D Conformal with FIF	Static tangents	Low energy MV photons	No	No	Breast	50	25	20	8.7	NS	NS	NS	27.0	17.0
			Supine	3D Conformal with FIF	Static tangents	Low energy MV photons	No	No	Breast	50	25	20	8.7	NS	NS	NS	27.0	17.0
Heymann	2011	France	Supine	3D Conformal plan	Static tangents	MV photons	No	NS	Breast	50	25	20	4.7	NS	NS	NS	NS	7.1
			Supine	3D IMRT	Static tangents	MV photons	No	NS	Breast	50	25	20	4.0	NS	NS	NS	NS	6.6
			Supine	3D IMRT	Static tangents	MV photons	No	NS	Breast	50	25	20	4.0	NS	NS	NS	NS	6.6
			Supine	3D IMRT	Static tangents	MV photons	No	NS	Breast	50	25	20	4.0	NS	NS	NS	NS	6.6
Hijal	2010	France	Supine	3D Conformal	Static tangents plus electron field	Mixed photons & electrons	No	Yes	Breast	50.68	28	13	6.4	NS	0.4	NS	NS	10.2
			Supine	3D IMRT	Rotational fields	MV photons	No	No	Breast	50.68	28	13	4.1	NS	0.3	NS	NS	6.3
			Supine	3D IMRT	Rotational fields	MV photons	No	No	Breast	50.68	28	13	4.1	NS	0.3	NS	NS	6.3
			Supine	3D IMRT	Rotational fields	MV photons	No	No	Breast	50.68	28	13	4.1	NS	0.3	NS	NS	6.3
Hjelstuen	2011	Norway	Supine	3D Conformal	Wide static tangents	Low energy MV photons	No	NS	Breast; SCF; axilla; IMC	50	25	17	21.7	17.2-24.9	NS	10.3	NS	44.5
			Supine	3D Conformal	Wide static tangents	Low energy MV photons	Yes	NS	Breast; SCF; axilla; IMC	50	25	17	16.4	12.6-21.0	NS	8.1	NS	32.7
			Supine	3D Conformal	Wide static tangents	Low energy MV photons	Yes	NS	Breast; SCF; axilla; IMC	50	25	17	16.4	12.6-21.0	NS	8.1	NS	32.7
			Supine	3D Conformal	Wide static tangents	Low energy MV photons	Yes	NS	Breast; SCF; axilla; IMC	50	25	17	16.4	12.6-21.0	NS	8.1	NS	32.7
Jagsi	2010	USA	Supine	3D IMRT	Oblique fields	Low energy MV photons	No	No	Breast; SCF; infraclav; IMC	52.2	30	10	18.0	NS	3.2	NS	93.5	34.5
			Supine	3D IMRT	Static tangents plus electron field	Mixed photons & electrons	No	No	Breast; SCF; infraclav; IMC	52.2	30	10	15.7	NS	0.5	NS	49.2	30.8
			Supine	3D IMRT	Static tangents plus electron field	Mixed photons & electrons	No	No	Breast; SCF; infraclav; IMC	52.2	30	10	15.7	NS	0.5	NS	49.2	30.8
			Supine	3D IMRT	Static tangents plus electron field	Mixed photons & electrons	No	No	Breast; SCF; infraclav; IMC	52.2	30	10	15.7	NS	0.5	NS	49.2	30.8
			Supine	3D Conformal with FIF	Static tangents plus electron field	Low energy MV photons	No	No	Breast; SCF; infraclav; IMC	52.2	30	10	13.8	NS	0.4	NS	46.2	27.6
			Supine	3D Conformal with FIF	Static tangents plus electron field	Low energy MV photons	No	No	Breast; SCF; infraclav; IMC	52.2	30	10	13.8	NS	0.4	NS	46.2	27.6
			Supine	3D Conformal with FIF	Static tangents plus electron field	Low energy MV photons	No	No	Breast; SCF; infraclav; IMC	52.2	30	10	13.8	NS	0.4	NS	46.2	27.6
			Supine	3D Conformal with FIF	Static tangents plus electron field	Low energy MV photons	No	No	Breast; SCF; infraclav; IMC	52.2	30	10	14.0	NS	0.7	NS	54.0	29.5
			Supine	3D Conformal with FIF	Static tangents plus electron field	Low energy MV photons	No	No	Breast; SCF; infraclav; IMC	52.2	30	10	14.0	NS	0.7	NS	54.0	29.5
			Supine	3D Conformal with FIF	Static tangents plus electron field	Low energy MV photons	No	No	Breast; SCF; infraclav; IMC	52.2	30	10	14.0	NS	0.7	NS	54.0	29.5
Jimenez	2013	USA	Supine	3D Conformal with FIF	Wide static tangents	MV photons	No	No	Chest wall; SCF; IMC +/- axilla	50	25	5	NS	NS	NS	NS	49.2	36.7
			Supine	3D Conformal with FIF	Static tangents plus electron field	Mixed photons & electrons	No	No	Chest wall; SCF; IMC +/- axilla	50	25	5	NS	NS	NS	NS	53.6	31.9

Table E3 continued on the next page.

Table E3 continued from the previous page.

Author	Year	Country	Description of regimens* Patient position	Treatment planning	Field type	Beam energy & modality	Breath. adapt.	RT plan delivered	Target(s)	Prescr. dose	No. of fraction	No. of CT plans	Lung dose measures MLD$_{ipsi}$ (Gy) Avg	MLD$_{ipsi}$ (Gy) Range	MLD$_{cont}$ (Gy)	MLD$_{whole}$ (Gy)	V5$_{ipsi}$ (%)	V20$_{ipsi}$ (%)
			Supine	3D Conformal with FIF	Static tangents plus electron field	Mixed photons & electrons	No	No	Chest wall; SCF; IMC +/- axilla	50	25	5	NS	NS	NS	NS	53.6	31.9
			Supine	3D Conformal with FIF	Static tangents plus electron field	Mixed photons & electrons	No	No	Chest wall; SCF; IMC +/- axilla	50	25	5	NS	NS	NS	NS	53.6	31.9
			Supine	3D IMRT	Tangents plus boost/direct fields	Protons	No	No	Chest wall; SCF; IMC +/- axilla	50	25	5	NS	NS	NS	NS	14.9	4.3
			Supine	3D IMRT	Tangents plus boost/direct fields	Protons	No	No	Chest wall; SCF; IMC +/- axilla	50	25	5	NS	NS	NS	NS	14.9	4.3
			Supine	3D IMRT	Tangents plus boost/direct fields	Protons	No	No	Chest wall; SCF; IMC +/- axilla	50	25	5	NS	NS	NS	NS	14.9	4.3
			Supine	3D Conformal with FIF	Wide static tangents	MV photons	No	No	Chest wall; SCF; IMC +/- axilla	50	25	5	NS	NS	NS	NS	53.2	38.3
			Supine	3D Conformal with FIF	Wide static tangents	MV photons	No	No	Chest wall; SCF; IMC +/- axilla	50	25	5	NS	NS	NS	NS	53.2	38.3
			Supine	3D Conformal with FIF	Wide static tangents	MV photons	No	No	Chest wall; SCF; IMC +/- axilla	50	25	5	NS	NS	NS	NS	53.2	38.3
			Supine	3D Conformal with FIF	Static tangents plus electron field	Mixed photons & electrons	No	No	Chest wall; SCF; IMC +/- axilla	50	25	5	NS	NS	NS	NS	65.1	36.4
			Supine	3D Conformal with FIF	Static tangents plus electron field	Mixed photons & electrons	No	No	Chest wall; SCF; IMC +/- axilla	50	25	5	NS	NS	NS	NS	65.1	36.4
			Supine	3D Conformal with FIF	Static tangents plus electron field	Mixed photons & electrons	No	No	Chest wall; SCF; IMC +/- axilla	50	25	5	NS	NS	NS	NS	65.1	36.4
			Supine	3D IMRT	Tangents plus boost/direct fields	Protons	No	No	Chest wall; SCF; IMC +/- axilla	50	25	5	NS	NS	NS	NS	13.5	4.1
			Supine	3D IMRT	Tangents plus boost/direct fields	Protons	No	No	Chest wall; SCF; IMC +/- axilla	50	25	5	NS	NS	NS	NS	13.5	4.1
			Supine	3D IMRT	Tangents plus boost/direct fields	Protons	No	No	Chest wall; SCF; IMC +/- axilla	50	25	5	NS	NS	NS	NS	13.5	4.1
Jin	2013	China	Supine	3D Conformal	Static tangents	Low energy MV photons	No	No	Breast	50	NS	20	8.6	NS	NS	NS	25.9	16.9
			Supine	3D Conformal with FIF	Static tangents	Low energy MV photons	No	No	Breast	50	NS	20	8.2	NS	NS	NS	24.6	15.0
			Supine	3D Conformal with FIF	Static tangents	Low energy MV photons	No	No	Breast	50	NS	20	8.2	NS	NS	NS	24.6	15.0
			Supine	3D Conformal with FIF	Static tangents	Low energy MV photons	No	No	Breast	50	NS	20	8.2	NS	NS	NS	24.6	15.0
			Supine	3D IMRT	Static tangents	Low energy MV photons	No	No	Breast	50	NS	20	6.8	NS	NS	NS	23.4	12.9
			Supine	3D IMRT	Static tangents	Low energy MV photons	No	No	Breast	50	NS	20	6.8	NS	NS	NS	23.4	12.9
			Supine	3D IMRT	Static tangents	Low energy MV photons	No	No	Breast	50	NS	20	6.8	NS	NS	NS	23.4	12.9
			Supine	3D IMRT	Oblique fields	Low energy MV photons	No	No	Breast	50	NS	20	9.3	NS	NS	NS	49.4	14.6
			Supine	3D IMRT	Oblique fields	Low energy MV photons	No	No	Breast	50	NS	20	9.3	NS	NS	NS	49.4	14.6
			Supine	3D IMRT	Oblique fields	Low energy MV photons	No	No	Breast	50	NS	20	9.3	NS	NS	NS	49.4	14.6
			Supine	3D IMRT	Rotational fields	Low energy MV photons	No	No	Breast	50	NS	20	10.1	NS	NS	NS	50.3	16.4
			Supine	3D IMRT	Rotational fields	Low energy MV photons	No	No	Breast	50	NS	20	10.1	NS	NS	NS	50.3	16.4
			Supine	3D IMRT	Rotational fields	Low energy MV photons	No	No	Breast	50	NS	20	10.1	NS	NS	NS	50.3	16.4
Johansen	2011	Norway	Supine	3D Conformal plan	Static tangents	MV photons	No	NS	Breast	50	25	16	13.6	9.8-17.0	NS	NS	NS	NS
			Supine	3D Conformal plan	Static tangents	MV photons	Yes	NS	Breast	50	25	16	12.4	8.1-15.8	NS	NS	NS	NS
			Supine	3D Conformal plan	Static tangents	MV photons	Yes	NS	Breast	50	25	16	12.4	8.1-15.8	NS	NS	NS	NS
			Supine	3D Conformal plan	Static tangents	MV photons	Yes	NS	Breast	50	25	16	12.4	8.1-15.8	NS	NS	NS	NS
Joosten	2013	Switzerland	Supine	2D Conformal	Static tangents	Low energy MV photons	No	No	Breast	50	25	1	5.7	NS	NS	NS	18.1	7.9
			Supine	3D Conformal	Static tangents	Low energy MV photons	No	No	Breast	50	25	1	5.0	NS	NS	NS	15.3	7.0
			Supine	3D Conformal	Static tangents	Low energy MV photons	No	No	Breast	50	25	1	5.0	NS	NS	NS	15.3	7.0
			Supine	3D Conformal	Static tangents	Low energy MV photons	No	No	Breast	50	25	1	5.0	NS	NS	NS	15.3	7.0

Table E3 continued on the next page.

Table E3 continued from the previous page.

Author	Year	Country	Description of regimens*									No. of CT plans	Lung dose measures					
			Patient position	Treatment planning	Field type	Beam energy & modality	Breath. adapt.	RT plan delivered	Target(s)	Prescr. dose	No. of fraction		MLD_{ipsi} (Gy)		MLD_{cont} (Gy)	MLD_{whole} (Gy)	$V5_{ipsi}$ (%)	$V20_{ipsi}$ (%)
													Avg	Range				
			Supine	3D IMRT	Static tangents	Low energy MV photons	No	No	Breast	50	25	1	4.5	NS	NS	NS	14.6	6.7
			Supine	3D IMRT	Static tangents	Low energy MV photons	No	No	Breast	50	25	1	4.5	NS	NS	NS	14.6	6.7
			Supine	3D IMRT	Static tangents	Low energy MV photons	No	No	Breast	50	25	1	4.5	NS	NS	NS	14.6	6.7
			Supine	2D Conformal	Static tangents	Low energy MV photons	No	No	Chest wall	50	25	1	3.9	NS	0.3	NS	11.8	4.7
			Supine	2D Conformal	Static tangents	Low energy MV photons	No	No	Chest wall	50	25	1	3.9	NS	0.3	NS	11.8	4.7
			Supine	2D Conformal	Static tangents	Low energy MV photons	No	No	Chest wall	50	25	1	3.9	NS	0.3	NS	11.8	4.7
			Supine	3D Conformal	Static tangents	Low energy MV photons	No	No	Chest wall	50	25	1	4.4	NS	0.4	NS	14.0	5.9
			Supine	3D Conformal	Static tangents	Low energy MV photons	No	No	Chest wall	50	25	1	4.4	NS	0.4	NS	14.0	5.9
			Supine	3D Conformal	Static tangents	Low energy MV photons	No	No	Chest wall	50	25	1	4.4	NS	0.4	NS	14.0	5.9
			Supine	3D IMRT	Static tangents	Low energy MV photons	No	No	Chest wall	50	25	1	3.9	NS	0.2	NS	13.5	5.9
			Supine	3D IMRT	Static tangents	Low energy MV photons	No	No	Chest wall	50	25	1	3.9	NS	0.2	NS	13.5	5.9
			Supine	3D IMRT	Static tangents	Low energy MV photons	No	No	Chest wall	50	25	1	3.9	NS	0.2	NS	13.5	5.9
			Supine	2D Conformal	Static tangents	Low energy MV photons	No	No	Chest wall	50	25	1	3.8	NS	NS	NS	NS	NS
			Supine	2D Conformal	Static tangents	Low energy MV photons	No	No	Chest wall	50	25	1	3.8	NS	NS	NS	NS	NS
			Supine	2D Conformal	Static tangents	Low energy MV photons	No	No	Chest wall	50	25	1	3.8	NS	NS	NS	NS	NS
			Supine	3D Conformal	Static tangents	Low energy MV photons	No	No	Chest wall	50	25	1	5.3	NS	NS	NS	NS	NS
			Supine	3D Conformal	Static tangents	Low energy MV photons	No	No	Chest wall	50	25	1	5.3	NS	NS	NS	NS	NS
			Supine	3D Conformal	Static tangents	Low energy MV photons	No	No	Chest wall	50	25	1	5.3	NS	NS	NS	NS	NS
			Supine	3D IMRT	Static tangents	Low energy MV photons	No	No	Chest wall	50	25	1	4.5	NS	NS	NS	NS	NS
			Supine	3D IMRT	Static tangents	Low energy MV photons	No	No	Chest wall	50	25	1	4.5	NS	NS	NS	NS	NS
			Supine	3D IMRT	Static tangents	Low energy MV photons	No	No	Chest wall	50	25	1	4.5	NS	NS	NS	NS	NS
Jost	2015	Germany	Supine	3D Conformal	Tangents plus boost/direct fields	Low energy MV photons	No	No	Breast	51	30	20	11.9	NS	0.6	NS	42.8	20.2
			Supine	3D IMRT	Rotational fields	Low energy MV photons	No	No	Breast	51	30	20	10.5	NS	0.6	NS	37.3	17.8
			Supine	3D IMRT	Rotational fields	Low energy MV photons	No	No	Breast	51	30	20	10.5	NS	0.6	NS	37.3	17.8
			Supine	3D IMRT	Rotational fields	Low energy MV photons	No	No	Breast	51	30	20	10.5	NS	0.6	NS	37.3	17.8
Kainz	2012	USA	Prone	3D IMRT	Rotational fields	MV photons	No	No	Breast or chest wall; SCF; axilla; IMC	45	25	5	10.0	6.6-14.4	3.8	NS	54.5	15.1
			Prone	3D IMRT	Rotational fields	MV photons	No	No	Breast or chest wall; SCF; axilla; IMC	45	25	6	8.8	5.5-11.7	2.8	NS	51.7	9.7
			Prone	3D IMRT	Rotational fields	MV photons	No	No	Breast or chest wall; SCF; axilla; IMC	45	25	6	8.8	5.5-11.7	2.8	NS	51.7	9.7
			Prone	3D IMRT	Rotational fields	MV photons	No	No	Breast or chest wall; SCF; axilla; IMC	45	25	6	8.8	5.5-11.7	2.8	NS	51.7	9.7
Khan	2015	USA	Supine	3D Conformal	Static tangents	Mixed energy MV photons	No	No	Breast	50	25	1	5.9	NS	NS	NS	NS	10.6
			Supine	3D IMRT	Static tangents	Low energy MV photons	No	No	Breast	50	25	1	5.0	NS	NS	NS	NS	8.4
			Supine	3D IMRT	Static tangents	Low energy MV photons	No	No	Breast	50	25	1	5.0	NS	NS	NS	NS	8.4
			Supine	3D IMRT	Static tangents	Low energy MV photons	No	No	Breast	50	25	1	5.0	NS	NS	NS	NS	8.4
			Supine	3D Conformal with FIF	Static tangents	Mixed energy MV photons	No	No	Breast	50	25	1	5.6	NS	NS	NS	NS	10.2
			Supine	3D Conformal with FIF	Static tangents	Mixed energy MV photons	No	No	Breast	50	25	1	5.6	NS	NS	NS	NS	10.2
			Supine	3D Conformal with FIF	Static tangents	Mixed energy MV photons	No	No	Breast	50	25	1	5.6	NS	NS	NS	NS	10.2
			Supine	3D IMRT	Static tangents	Low energy MV photons	No	No	Breast	50	25	1	4.3	NS	NS	NS	NS	7.8
			Supine	3D IMRT	Static tangents	Low energy MV photons	No	No	Breast	50	25	1	4.3	NS	NS	NS	NS	7.8
			Supine	3D IMRT	Static tangents	Low energy MV photons	No	No	Breast	50	25	1	4.3	NS	NS	NS	NS	7.8
Kim	2014	Korea	Supine	3D Conformal with FIF	Tangents plus boost/direct fields	MV photons	No	Yes	Breast; +/- SCF	50.4	28	157	6.5	2.8-18.7	NS	NS	NS	11.8

Table E3 continued on the next page.

Table E3 continued from the previous page.

Author	Year	Country	Description of regimens*									No. of CT plans	Lung dose measures					
			Patient position	Treatment planning	Field type	Beam energy & modality	Breath. adapt.	RT plan delivered	Target(s)	Prescr. dose	No. of fraction		MLD_{ipsi} (Gy)		MLD_{cont} (Gy)	MLD_{whole} (Gy)	$V5_{ipsi}$ (%)	$V20_{ipsi}$ (%)
													Avg	Range				
Kirby	2010	UK	Supine	3D Conformal	Static tangents	MV photons	No	NS	Breast	50	25	65	4.4	3.5-5.2	NS	NS	NS	NS
			Supine	3D Conformal	Static tangents	MV photons	No	NS	Partial breast	50	25	65	1.2	0.8-2.1	NS	NS	NS	NS
			Supine	3D Conformal	Static tangents	MV photons	No	NS	Partial breast	50	25	65	1.2	0.8-2.1	NS	NS	NS	NS
			Supine	3D Conformal	Static tangents	MV photons	No	NS	Partial breast	50	25	65	1.2	0.8-2.1	NS	NS	NS	NS
			Prone	3D Conformal	Static tangents	MV photons	No	NS	Breast	50	25	65	0.8	0.3-1.4	NS	NS	NS	NS
			Prone	3D Conformal	Static tangents	MV photons	No	NS	Breast	50	25	65	0.8	0.3-1.4	NS	NS	NS	NS
			Prone	3D Conformal	Static tangents	MV photons	No	NS	Breast	50	25	65	0.8	0.3-1.4	NS	NS	NS	NS
			Prone	3D Conformal	Static tangents	MV photons	No	NS	Partial breast	50	25	65	0.4	0.2-0.8	NS	NS	NS	NS
			Prone	3D Conformal	Static tangents	MV photons	No	NS	Partial breast	50	25	65	0.4	0.2-0.8	NS	NS	NS	NS
			Prone	3D Conformal	Static tangents	MV photons	No	NS	Partial breast	50	25	65	0.4	0.2-0.8	NS	NS	NS	NS
Kirova	2014	France	Lateral Decubitus	3D Conformal	Tangents plus boost/direct fields	Low energy MV photons	No	Yes	Breast	50	25	56	1.0	NS	NS	NS	0.7	NS
Koh	2013	Singapore	Supine	3D Conformal	Tangents plus boost/direct fields	MV photons	No	NS	Breast; SCF	50	25	15	4.4	NS	NS	NS	NS	NS
			Supine	3D Conformal	Tangents plus boost/direct fields	MV photons	No	NS	Breast; SCF	50	25	15	4.7	NS	NS	NS	NS	NS
			Supine	3D Conformal	Tangents plus boost/direct fields	MV photons	No	NS	Breast; SCF	50	25	15	4.7	NS	NS	NS	NS	NS
			Supine	3D Conformal	Tangents plus boost/direct fields	MV photons	No	NS	Breast; SCF	50	25	15	4.7	NS	NS	NS	NS	NS
			Supine	3D IMRT	Oblique fields	MV photons	No	No	Breast; SCF	50	25	15	4.0	NS	NS	NS	NS	NS
			Supine	3D IMRT	Oblique fields	MV photons	No	No	Breast; SCF	50	25	15	4.0	NS	NS	NS	NS	NS
			Supine	3D IMRT	Oblique fields	MV photons	No	No	Breast; SCF	50	25	15	4.0	NS	NS	NS	NS	NS
Krengli	2013	Italy	Supine	3D Conformal	Static tangents plus electron field	MV photons	No	NS	Breast	50	25	41	5.2	NS	NS	NS	18.4	9.0
			Prone	3D Conformal	Static tangents plus electron field	Mixed energy MV photons	No	NS	Breast	50	25	41	1.4	NS	NS	NS	4.0	1.5
			Prone	3D Conformal	Static tangents plus electron field	Mixed energy MV photons	No	NS	Breast	50	25	41	1.4	NS	NS	NS	4.0	1.5
			Prone	3D Conformal	Static tangents plus electron field	Mixed energy MV photons	No	NS	Breast	50	25	41	1.4	NS	NS	NS	4.0	1.5
Lakosi	2015	Belgium	Supine	3D Conformal with FIF	Static tangents	Low energy MV photons	No	Yes	Breast	40.5	15	9	3.5	NS	NS	NS	NS	NS
			Supine	3D Conformal with FIF	Static tangents	Low energy MV photons	No	No	Breast	40.5	15	9	4.3	NS	NS	NS	NS	NS
			Supine	3D Conformal with FIF	Static tangents	Low energy MV photons	No	No	Breast	40.5	15	9	4.3	NS	NS	NS	NS	NS
			Supine	3D Conformal with FIF	Static tangents	Low energy MV photons	No	No	Breast	40.5	15	9	4.3	NS	NS	NS	NS	NS
			Prone	3D Conformal with FIF	Static tangents	Low energy MV photons	No	No	Breast	40.5	15	9	1.0	NS	NS	NS	NS	NS
			Prone	3D Conformal with FIF	Static tangents	Low energy MV photons	No	No	Breast	40.5	15	9	1.0	NS	NS	NS	NS	NS
			Prone	3D Conformal with FIF	Static tangents	Low energy MV photons	No	No	Breast	40.5	15	9	1.0	NS	NS	NS	NS	NS
			Prone	3D Conformal with FIF	Static tangents	Low energy MV photons	No	No	Breast	40.5	15	9	1.3	NS	NS	NS	NS	NS
			Prone	3D Conformal with FIF	Static tangents	Low energy MV photons	No	No	Breast	40.5	15	9	1.3	NS	NS	NS	NS	NS
			Prone	3D Conformal with FIF	Static tangents	Low energy MV photons	No	No	Breast	40.5	15	9	1.3	NS	NS	NS	NS	NS
Lamberth	2014	France	Supine	3D Conformal	Static tangents	MV photons	No	No	Breast or chest wall; SCF; axilla; +/- IMC	50	25	9	NS	NS	NS	NS	7.8	2.8
			Supine	3D IMRT	Rotational fields	Low energy MV photons	No	Yes	Breast or chest wall; SCF; axilla; +/- IMC	50	25	9	NS	NS	NS	NS	66.3	2.2

Table E3 continued on the next page.

Table E3 continued from the previous page.

Author	Year	Country	Description of regimens*									No. of CT plans	Lung dose measures					
			Patient position	Treatment planning	Field type	Beam energy & modality	Breath. adapt.	RT plan delivered	Target(s)	Prescr. dose	No. of fraction		MLD$_{ipsi}$ (Gy)		MLD$_{cont}$ (Gy)	MLD$_{whole}$ (Gy)	V5$_{ipsi}$ (%)	V20$_{ipsi}$ (%)
													Avg	Range				
			Supine	3D IMRT	Rotational fields	Low energy MV photons	No	Yes	Breast or chest wall; SCF; axilla; +/- IMC	50	25	9	NS	NS	NS	NS	66.3	2.2
			Supine	3D IMRT	Rotational fields	Low energy MV photons	No	Yes	Breast or chest wall; SCF; axilla; +/- IMC	50	25	9	NS	NS	NS	NS	66.3	2.2
Lee H	2013	Korea	Supine	3D Conformal	Static tangents	MV photons	No	No	Breast	50.4	1.8	25	8.0	5.4-12.2	NS	NS	NS	15.7
			Supine	3D Conformal	Static tangents	MV photons	Yes	Yes	Breast	50.4	1.8	25	7.5	5.1-10.4	NS	NS	NS	14.6
			Supine	3D Conformal	Static tangents	MV photons	Yes	Yes	Breast	50.4	1.8	25	7.5	5.1-10.4	NS	NS	NS	14.6
			Supine	3D Conformal	Static tangents	MV photons	Yes	Yes	Breast	50.4	1.8	25	7.5	5.1-10.4	NS	NS	NS	14.6
Lee J	2013	Korea	Supine	3D Conformal	Static tangents	Low energy MV photons	No	Yes	Chest wall	50	25	12	9.1	NS	0.5	NS	NS	15.0
			Supine	3D Conformal with FIF	Static tangents	Low energy MV photons	No	No	Chest wall	50	25	12	9.0	NS	0.2	NS	NS	15.1
			Supine	3D Conformal with FIF	Static tangents	Low energy MV photons	No	No	Chest wall	50	25	12	9.0	NS	0.2	NS	NS	15.1
			Supine	3D Conformal with FIF	Static tangents	Low energy MV photons	No	No	Chest wall	50	25	12	9.0	NS	0.2	NS	NS	15.1
			Supine	3D IMRT	Direct fields	Low energy MV photons	No	No	Chest wall	50	25	12	11.7	NS	3.1	NS	NS	11.7
			Supine	3D IMRT	Direct fields	Low energy MV photons	No	No	Chest wall	50	25	12	11.7	NS	3.1	NS	NS	11.7
			Supine	3D IMRT	Direct fields	Low energy MV photons	No	No	Chest wall	50	25	12	11.7	NS	3.1	NS	NS	11.7
			Supine	3D IMRT	Rotational fields	Low energy MV photons	No	No	Chest wall	50	25	12	13.4	NS	5.4	NS	NS	15.4
			Supine	3D IMRT	Rotational fields	Low energy MV photons	No	No	Chest wall	50	25	12	13.4	NS	5.4	NS	NS	15.4
			Supine	3D IMRT	Rotational fields	Low energy MV photons	No	No	Chest wall	50	25	12	13.4	NS	5.4	NS	NS	15.4
			Supine	3D IMRT	Other	Low energy MV photons	No	No	Chest wall	50	25	12	10.4	NS	1.7	NS	NS	13.9
			Supine	3D IMRT	Other	Low energy MV photons	No	No	Chest wall	50	25	12	10.4	NS	1.7	NS	NS	13.9
			Supine	3D IMRT	Other	Low energy MV photons	No	No	Chest wall	50	25	12	10.4	NS	1.7	NS	NS	13.9
Li	2014	USA	Supine	3D Conformal	Static tangents	MV photons	No	No	Breast	50	25	1	6.4	NS	NS	NS	20.8	11.8
			Supine	3D IMRT	Other	MV photons	No	No	Breast	50	25	1	4.0	NS	NS	NS	16.6	5.6
			Supine	3D IMRT	Other	MV photons	No	No	Breast	50	25	1	4.0	NS	NS	NS	16.6	5.6
			Supine	3D IMRT	Other	MV photons	No	No	Breast	50	25	1	4.0	NS	NS	NS	16.6	5.6
			Supine	3D IMRT	Rotational fields	MV photons	No	No	Breast	50	25	1	18.9	NS	NS	NS	89.5	37.2
			Supine	3D IMRT	Rotational fields	MV photons	No	No	Breast	50	25	1	18.9	NS	NS	NS	89.5	37.2
			Supine	3D IMRT	Rotational fields	MV photons	No	No	Breast	50	25	1	18.9	NS	NS	NS	89.5	37.2
			Supine	3D Conformal	Static tangents	MV photons	No	No	Chest wall	50	25	1	8.2	NS	NS	NS	25.5	15.1
			Supine	3D Conformal	Static tangents	MV photons	No	No	Chest wall	50	25	1	8.2	NS	NS	NS	25.5	15.1
			Supine	3D Conformal	Static tangents	MV photons	No	No	Chest wall	50	25	1	8.2	NS	NS	NS	25.5	15.1
			Supine	3D IMRT	Other	MV photons	No	No	Chest wall	50	25	1	4.1	NS	NS	NS	18.1	4.9
			Supine	3D IMRT	Other	MV photons	No	No	Chest wall	50	25	1	4.1	NS	NS	NS	18.1	4.9
			Supine	3D IMRT	Other	MV photons	No	No	Chest wall	50	25	1	4.1	NS	NS	NS	18.1	4.9
			Supine	3D IMRT	Rotational fields	MV photons	No	No	Chest wall	50	25	1	10.4	NS	NS	NS	74.1	13.6
			Supine	3D IMRT	Rotational fields	MV photons	No	No	Chest wall	50	25	1	10.4	NS	NS	NS	74.1	13.6
			Supine	3D IMRT	Rotational fields	MV photons	No	No	Chest wall	50	25	1	10.4	NS	NS	NS	74.1	13.6
Liljegren	2014	Sweden	Supine	3D Conformal	Static tangents	Low energy MV photons	No	Yes	Chest wall	50	25	80	8.9	NS	NS	NS	NS	15.8
			Supine	3D Conformal	Static tangents	Low energy MV photons	No	Yes	Chest wall	50	NS	162	8.9	NS	NS	NS	NS	16.7
			Supine	3D Conformal	Static tangents	Low energy MV photons	No	Yes	Chest wall	50	NS	162	8.9	NS	NS	NS	NS	16.7
			Supine	3D Conformal	Static tangents	Low energy MV photons	No	Yes	Chest wall	50	NS	162	8.9	NS	NS	NS	NS	16.7
			Supine	3D Conformal	Static tangents	Low energy MV photons	No	Yes	Chest wall; SCF; infraclav	50	25	82	13.8	NS	NS	NS	NS	28.1
			Supine	3D Conformal	Static tangents	Low energy MV photons	No	Yes	Chest wall; SCF; infraclav	50	25	82	13.8	NS	NS	NS	NS	28.1
			Supine	3D Conformal	Static tangents	Low energy MV photons	No	Yes	Chest wall; SCF; infraclav	50	25	82	13.8	NS	NS	NS	NS	28.1

Table E3 continued on the next page.

Table E3 continued from the previous page.

Author	Year	Country	Description of regimens*									No. of CT plans	Lung dose measures					
			Patient position	Treatment planning	Field type	Beam energy & modality	Breath. adapt.	RT plan delivered	Target(s)	Prescr. dose	No. of fraction		MLD_{ipsi} (Gy) Avg	MLD_{ipsi} (Gy) Range	MLD_{cont} (Gy)	MLD_{whole} (Gy)	$V5_{ipsi}$ (%)	$V20_{ipsi}$ (%)
			Supine	3D Conformal	Static tangents	Low energy MV photons	No	Yes	Chest wall; SCF; infraclav	50	NS	494	14.3	NS	NS	NS	NS	28.8
			Supine	3D Conformal	Static tangents	Low energy MV photons	No	Yes	Chest wall; SCF; infraclav	50	NS	494	14.3	NS	NS	NS	NS	28.8
			Supine	3D Conformal	Static tangents	Low energy MV photons	No	Yes	Chest wall; SCF; infraclav	50	NS	494	14.3	NS	NS	NS	NS	28.8
Lin J	2015	Taiwan	Supine	3D IMRT	Oblique fields	Low energy MV photons	No	Yes	Breast	50.4	28	10	11.6	NS	5.2	NS	72.4	15.2
			Supine	3D IMRT	Rotational fields	Low energy MV photons	No	No	Breast	50.4	28	10	11.5	NS	5.9	NS	74.2	15.9
			Supine	3D IMRT	Rotational fields	Low energy MV photons	No	No	Breast	50.4	28	10	11.5	NS	5.9	NS	74.2	15.9
			Supine	3D IMRT	Rotational fields	Low energy MV photons	No	No	Breast	50.4	28	10	11.5	NS	5.9	NS	74.2	15.9
			Supine	3D IMRT	Rotational fields	Low energy MV photons	No	No	Breast	50.4	28	10	10.2	NS	2.5	NS	48.6	14.5
			Supine	3D IMRT	Rotational fields	Low energy MV photons	No	No	Breast	50.4	28	10	10.2	NS	2.5	NS	48.6	14.5
			Supine	3D IMRT	Rotational fields	Low energy MV photons	No	No	Breast	50.4	28	10	10.2	NS	2.5	NS	48.6	14.5
Lin L	2015	USA	Supine	3D IMRT	Static tangents	Low energy MV photons	Yes	Yes	Breast	50	25	10	7.3	NS	NS	NS	25.2	12.5
			Supine	3D IMRT	Direct fields	Protons	Yes	No	Breast	50	25	10	1.5	NS	NS	NS	7.5	2.5
			Supine	3D IMRT	Direct fields	Protons	Yes	No	Breast	50	25	10	1.5	NS	NS	NS	7.5	2.5
			Supine	3D IMRT	Direct fields	Protons	Yes	No	Breast	50	25	10	1.5	NS	NS	NS	7.5	2.5
			Supine	3D IMRT	Direct fields	Protons	Yes	No	Breast	50	25	10	0.9	NS	NS	NS	4.7	0.0
			Supine	3D IMRT	Direct fields	Protons	Yes	No	Breast	50	25	10	0.9	NS	NS	NS	4.7	0.0
			Supine	3D IMRT	Direct fields	Protons	Yes	No	Breast	50	25	10	0.9	NS	NS	NS	4.7	0.0
Lin Y	2015	China	Supine	3D Conformal	Static tangents	Low energy MV photons	No	No	Breast	50	25	20	NS	NS	NS	NS	38.3	27.7
			Supine	3D IMRT	Oblique fields	Low energy MV photons	No	No	Breast	50	25	20	NS	NS	NS	NS	27.5	20.8
			Supine	3D IMRT	Oblique fields	Low energy MV photons	No	No	Breast	50	25	20	NS	NS	NS	NS	27.5	20.8
			Supine	3D IMRT	Oblique fields	Low energy MV photons	No	No	Breast	50	25	20	NS	NS	NS	NS	27.5	20.8
Liu	2015	China	Supine	3D IMRT	Static tangents	Low energy MV phtons	No	NS	Breast	50	25	15	12.6	NS	1.7	NS	NS	NS
			Supine	3D IMRT	Rotational fields	Low energy MV phtons	No	NS	Breast	50	25	15	12.6	NS	3.3	NS	NS	NS
			Supine	3D IMRT	Rotational fields	Low energy MV phtons	No	NS	Breast	50	25	15	12.6	NS	3.3	NS	NS	NS
			Supine	3D IMRT	Rotational fields	Low energy MV phtons	No	NS	Breast	50	25	15	12.6	NS	3.3	NS	NS	NS
			Supine	3D IMRT	Rotational fields	Low energy MV phtons	No	NS	Breast	50	25	15	12.7	NS	1.0	NS	NS	NS
			Supine	3D IMRT	Rotational fields	Low energy MV phtons	No	NS	Breast	50	25	15	12.7	NS	1.0	NS	NS	NS
			Supine	3D IMRT	Rotational fields	Low energy MV phtons	No	NS	Breast	50	25	15	12.7	NS	1.0	NS	NS	NS
			Supine	3D IMRT	Static tangents	Low energy MV phtons	No	NS	Breast	50	25	15	13.3	NS	NS	NS	NS	NS
			Supine	3D IMRT	Static tangents	Low energy MV phtons	No	NS	Breast	50	25	15	13.3	NS	NS	NS	NS	NS
			Supine	3D IMRT	Static tangents	Low energy MV phtons	No	NS	Breast	50	25	15	13.3	NS	NS	NS	NS	NS
			Supine	3D IMRT	Rotational fields	Low energy MV phtons	No	NS	Breast	50	25	15	13.3	NS	NS	NS	NS	NS
			Supine	3D IMRT	Rotational fields	Low energy MV phtons	No	NS	Breast	50	25	15	13.3	NS	NS	NS	NS	NS
			Supine	3D IMRT	Rotational fields	Low energy MV phtons	No	NS	Breast	50	25	15	13.3	NS	NS	NS	NS	NS
			Supine	3D IMRT	Rotational fields	Low energy MV phtons	No	NS	Breast	50	25	15	12.9	NS	NS	NS	NS	NS
			Supine	3D IMRT	Rotational fields	Low energy MV phtons	No	NS	Breast	50	25	15	12.9	NS	NS	NS	NS	NS
			Supine	3D IMRT	Rotational fields	Low energy MV phtons	No	NS	Breast	50	25	15	12.9	NS	NS	NS	NS	NS
Lymberis	2012	USA	Supine	3D IMRT	Static tangents	MV photons	No	Yes	Breast	40.5	15	7	NS	NS	NS	3.1	8.1	NS
			Prone	3D IMRT	Static tangents	MV photons	No	Yes	Breast	40.5	15	46	NS	NS	NS	0.8	0.8	NS
			Prone	3D IMRT	Static tangents	MV photons	No	Yes	Breast	40.5	15	46	NS	NS	NS	0.8	0.8	NS
			Prone	3D IMRT	Static tangents	MV photons	No	Yes	Breast	40.5	15	46	NS	NS	NS	0.8	0.8	NS
			Prone	3D IMRT	Static tangents	MV photons	No	Yes	Breast	40.5	15	47	NS	NS	NS	0.9	1.4	NS

Table E3 continued on the next page.

Table E3 continued from the previous page.

Author	Year	Country	Description of regimens* Patient position	Treatment planning	Field type	Beam energy & modality	Breath. adapt.	RT plan delivered	Target(s)	Prescr. dose	No. of fraction	No. of CT plans	Lung dose measures MLD$_{ipsi}$ (Gy) Avg	MLD$_{ipsi}$ (Gy) Range	MLD$_{cont}$ (Gy)	MLD$_{whole}$ (Gy)	V5$_{ipsi}$ (%)	V20$_{ipsi}$ (%)
			Prone	3D IMRT	Static tangents	MV photons	No	Yes	Breast	40.5	15	47	NS	NS	NS	0.9	1.4	NS
			Prone	3D IMRT	Static tangents	MV photons	No	Yes	Breast	40.5	15	47	NS	NS	NS	0.9	1.4	NS
Ma C	2015	China	Supine	3D Conformal with FIF	Static tangents	MV photons	No	No	Chest wall; SCF	50	25	10	15.3	NS	NS	NS	49.6	31.4
			Supine	3D IMRT	Oblique fields	MV photons	No	No	Chest wall; SCF	50	25	10	15.0	NS	NS	NS	52.5	27.8
			Supine	3D IMRT	Oblique fields	MV photons	No	No	Chest wall; SCF	50	25	10	15.0	NS	NS	NS	52.5	27.8
			Supine	3D IMRT	Oblique fields	MV photons	No	No	Chest wall; SCF	50	25	10	15.0	NS	NS	NS	52.5	27.8
			Supine	3D IMRT	Rotational fields	MV photons	No	No	Chest wall; SCF	50	25	10	18.6	NS	NS	NS	70.4	34.1
			Supine	3D IMRT	Rotational fields	MV photons	No	No	Chest wall; SCF	50	25	10	18.6	NS	NS	NS	70.4	34.1
			Supine	3D IMRT	Rotational fields	MV photons	No	No	Chest wall; SCF	50	25	10	18.6	NS	NS	NS	70.4	34.1
Ma J	2013	China	Supine	3D IMRT	Static tangents	Low energy MV photons	No	Yes	Chest wall; SCF; infraclav; +/- IMC	50	25	85	15.1	NS	2.3	NS	65.0	28.0
MacDonald	2013	USA	Supine	3D IMRT	Direct fields	Protons	No	Yes	Chest wall; SCF; IMC +/- axilla	50.4	28	12	NS	NS	NS	6.0	NS	12.7
Majumdar	2011	Saudi Arabia	Supine	3D IMRT	Oblique fields	MV photons	Yes	Yes	Breast	50.4	28	45	21.7	NS	NS	NS	NS	37.4
Mancosu	2014	Italy	Supine	3D IMRT	Rotational fields	Low energy MV photons	No	No	Breast	40.5	15	10	7.5	NS	NS	NS	NS	7.4
Mansouri	2014	Spain	Supine	3D Conformal	Static tangents	MV photons	No	No	Breast	50	25	20	NS	NS	NS	NS	25.0	19.0
			Supine	3D IMRT	Oblique fields	MV photons	No	No	Breast	50	25	20	NS	NS	NS	NS	90.0	13.0
			Supine	3D IMRT	Oblique fields	MV photons	No	No	Breast	50	25	20	NS	NS	NS	NS	90.0	13.0
			Supine	3D IMRT	Oblique fields	MV photons	No	No	Breast	50	25	20	NS	NS	NS	NS	90.0	13.0
Mast Kempen	2013	Netherlands	Supine	3D Conformal	Static tangents	MV photons	No	No	Breast	42.56	16	20	NS	NS	NS	3.3	11.3	6.8
			Supine	3D Conformal	Static tangents	MV photons	Yes	Yes	Breast	42.56	16	20	NS	NS	NS	3.0	11.4	6.2
			Supine	3D Conformal	Static tangents	MV photons	Yes	Yes	Breast	42.56	16	20	NS	NS	NS	3.0	11.4	6.2
			Supine	3D Conformal	Static tangents	MV photons	Yes	Yes	Breast	42.56	16	20	NS	NS	NS	3.0	11.4	6.2
			Supine	3D IMRT	Static tangents	MV photons	No	No	Breast	42.56	16	20	NS	NS	NS	2.9	10.1	5.7
			Supine	3D IMRT	Static tangents	MV photons	No	No	Breast	42.56	16	20	NS	NS	NS	2.9	10.1	5.7
			Supine	3D IMRT	Static tangents	MV photons	No	No	Breast	42.56	16	20	NS	NS	NS	2.9	10.1	5.7
			Supine	3D IMRT	Static tangents	MV photons	Yes	No	Breast	42.56	16	20	NS	NS	NS	2.6	10.1	5.1
			Supine	3D IMRT	Static tangents	MV photons	Yes	No	Breast	42.56	16	20	NS	NS	NS	2.6	10.1	5.1
			Supine	3D IMRT	Static tangents	MV photons	Yes	No	Breast	42.56	16	20	NS	NS	NS	2.6	10.1	5.1
Mast Vredeveld	2014	Netherlands	Supine	3D IMRT	Oblique fields	Protons	No	No	Breast	42.6	16	20	1.6	NS	NS	0.9	NS	2.8
			Supine	3D IMRT	Oblique fields	Protons	Yes	No	Breast	42.6	16	20	1.5	NS	NS	0.8	NS	2.5
			Supine	3D IMRT	Oblique fields	Protons	Yes	No	Breast	42.6	16	20	1.5	NS	NS	0.8	NS	2.5
			Supine	3D IMRT	Oblique fields	Protons	Yes	No	Breast	42.6	16	20	1.5	NS	NS	0.8	NS	2.5
			Supine	3D IMRT	Static tangents	Low energy MV phtons	No	No	Breast	42.6	16	20	6.1	NS	NS	2.9	NS	12.4
			Supine	3D IMRT	Static tangents	Low energy MV phtons	No	No	Breast	42.6	16	20	6.1	NS	NS	2.9	NS	12.4
			Supine	3D IMRT	Static tangents	Low energy MV phtons	No	No	Breast	42.6	16	20	6.1	NS	NS	2.9	NS	12.4
			Supine	3D IMRT	Static tangents	Low energy MV phtons	Yes	No	Breast	42.6	16	20	5.4	NS	NS	2.6	NS	10.9

Table E3 continued on the next page.

Table E3 continued from the previous page.

Author	Year	Country	Description of regimens*										Lung dose measures					
			Patient position	Treatment planning	Field type	Beam energy & modality	Breath. adapt.	RT plan delivered	Target(s)	Prescr. dose	No. of fraction	No. of CT plans	MLD_{ipsi} (Gy)		MLD_{cont} (Gy)	MLD_{whole} (Gy)	$V5_{ipsi}$ (%)	$V20_{ipsi}$ (%)
													Avg	Range				
			Supine	3D IMRT	Static tangents	Low energy MV phtons	Yes	No	Breast	42.6	16	20	5.4	NS	NS	2.6	NS	10.9
			Supine	3D IMRT	Static tangents	Low energy MV phtons	Yes	No	Breast	42.6	16	20	5.4	NS	NS	2.6	NS	10.9
Mavroidis	2011	Sweden	Supine	3D IMRT	Tangents plus boost/direct fields	Low energy MV photons	No	Yes	Breast	35	25	1	8.2	NS	0.7	NS	NS	NS
Mayadagli	2011	Turkey	Supine	2D Conformal plan	Static tangents	NS	No	No	Breast	50	25	21	NS	NS	NS	3.6	NS	NS
			Supine	3D Conformal plan	Static tangents	NS	No	Yes	Breast	50	25	21	NS	NS	NS	7.1	NS	NS
			Supine	3D Conformal plan	Static tangents	NS	No	Yes	Breast	50	25	21	NS	NS	NS	7.1	NS	NS
			Supine	3D Conformal plan	Static tangents	NS	No	Yes	Breast	50	25	21	NS	NS	NS	7.1	NS	NS
Mezenski	2012	Poland	Supine	3D Conformal	Static tangents	Low energy MV photons	No	NS	Breast	50	25	5	4.8	NS	0.4	NS	NS	7.4
			Supine	3D IMRT	Oblique fields	Low energy MV photons	No	NS	Breast	50	25	5	14.7	NS	3.4	NS	NS	20.0
			Supine	3D IMRT	Oblique fields	Low energy MV photons	No	NS	Breast	50	25	5	14.7	NS	3.4	NS	NS	20.0
			Supine	3D IMRT	Oblique fields	Low energy MV photons	No	NS	Breast	50	25	5	14.7	NS	3.4	NS	NS	20.0
			Supine	3D Conformal	Static tangents	Low energy MV photons	No	NS	Breast; SCF; axilla	50	25	NS	13.1	NS	0.8	NS	NS	26.3
			Supine	3D Conformal	Static tangents	Low energy MV photons	No	NS	Breast; SCF; axilla	50	25	NS	13.1	NS	0.8	NS	NS	26.3
			Supine	3D Conformal	Static tangents	Low energy MV photons	No	NS	Breast; SCF; axilla	50	25	NS	13.1	NS	0.8	NS	NS	26.3
			Supine	3D IMRT	Oblique fields	Low energy MV photons	No	NS	Breast; SCF; axilla	50	25	NS	18.3	NS	5.9	NS	NS	27.3
			Supine	3D IMRT	Oblique fields	Low energy MV photons	No	NS	Breast; SCF; axilla	50	25	NS	18.3	NS	5.9	NS	NS	27.3
			Supine	3D IMRT	Oblique fields	Low energy MV photons	No	NS	Breast; SCF; axilla	50	25	NS	18.3	NS	5.9	NS	NS	27.3
			Supine	3D Conformal	Static tangents	Low energy MV photons	No	NS	Breast; SCF; axilla	50	25	NS	9.3	NS	0.6	NS	NS	16.3
			Supine	3D Conformal	Static tangents	Low energy MV photons	No	NS	Breast; SCF; axilla	50	25	NS	9.3	NS	0.6	NS	NS	16.3
			Supine	3D Conformal	Static tangents	Low energy MV photons	No	NS	Breast; SCF; axilla	50	25	NS	9.3	NS	0.6	NS	NS	16.3
			Supine	3D IMRT	Oblique fields	Low energy MV photons	No	NS	Breast; SCF; axilla	50	25	NS	16.1	NS	5.4	NS	NS	27.5
			Supine	3D IMRT	Oblique fields	Low energy MV photons	No	NS	Breast; SCF; axilla	50	25	NS	16.1	NS	5.4	NS	NS	27.5
			Supine	3D IMRT	Oblique fields	Low energy MV photons	No	NS	Breast; SCF; axilla	50	25	NS	16.1	NS	5.4	NS	NS	27.5
Michalski	2014	Australia	Supine	3D Conformal	Static tangents	Mixed energy MV photons	No	No	Breast	45	20	20	8.3	NS	0.2	3.9	27.5	14.6
			Supine	3D IMRT	Static tangents	Low energy MV photons	No	No	Breast	45	20	20	7.8	NS	0.2	3.6	26.8	14.8
			Supine	3D IMRT	Static tangents	Low energy MV photons	No	No	Breast	45	20	20	7.8	NS	0.2	3.6	26.8	14.8
			Supine	3D IMRT	Static tangents	Low energy MV photons	No	No	Breast	45	20	20	7.8	NS	0.2	3.6	26.8	14.8
			Supine	3D IMRT	Static tangents	Low energy MV photons	No	No	Breast	45	20	20	6.8	NS	0.5	3.4	28.6	11.9
			Supine	3D IMRT	Static tangents	Low energy MV photons	No	No	Breast	45	20	20	6.8	NS	0.5	3.4	28.6	11.9
			Supine	3D IMRT	Static tangents	Low energy MV photons	No	No	Breast	45	20	20	6.8	NS	0.5	3.4	28.6	11.9
Mulliez Cancer Radiot	2014	Belgium	Prone	3D IMRT	Static tangents	Low energy MV photons	No	No	Breast	40.05	15	20	1.0	NS	NS	NS	2.7	NS
			Prone	3D IMRT	Static tangents	Low energy MV photons	Yes	No	Breast	40.05	15	20	0.9	NS	NS	NS	2.7	NS
			Prone	3D IMRT	Static tangents	Low energy MV photons	Yes	No	Breast	40.05	15	20	0.9	NS	NS	NS	2.7	NS
			Prone	3D IMRT	Static tangents	Low energy MV photons	Yes	No	Breast	40.05	15	20	0.9	NS	NS	NS	2.7	NS
Mulliez hypo	2013	Belgium	Supine	3D IMRT	Static tangents	Low energy MV photons	No	Yes	Breast	40.05	15	50	3.8	NS	NS	NS	16.9	5.5
			Prone	3D IMRT	Static tangents	Low energy MV photons	No	Yes	Breast	40.05	15	50	1.1	NS	NS	NS	2.9	0.9
			Prone	3D IMRT	Static tangents	Low energy MV photons	No	Yes	Breast	40.05	15	50	1.1	NS	NS	NS	2.9	0.9
			Prone	3D IMRT	Static tangents	Low energy MV photons	No	Yes	Breast	40.05	15	50	1.1	NS	NS	NS	2.9	0.9
Mulliez IMRT	2013	Belgium	Supine	3D Conformal	Static tangents	MV photons	No	No	Breast	50	25	18	7.7	NS	NS	NS	NS	13.5

Table E3 continued on the next page.

Table E3 continued from the previous page.

Author	Year	Country	Description of regimens*									No. of CT plans	Lung dose measures					
			Patient position	Treatment planning	Field type	Beam energy & modality	Breath. adapt.	RT plan delivered	Target(s)	Prescr. dose	No. of fraction		MLD_{ipsi} (Gy)		MLD_{cont} (Gy)	MLD_{whole} (Gy)	$V5_{ipsi}$ (%)	$V20_{ipsi}$ (%)
													Avg	Range				
			Prone	3D Conformal	Static tangents	MV photons	No	No	Breast	50	25	18	1.2	NS	NS	NS	NS	0.9
			Prone	3D Conformal	Static tangents	MV photons	No	No	Breast	50	25	18	1.2	NS	NS	NS	NS	0.9
			Prone	3D Conformal	Static tangents	MV photons	No	No	Breast	50	25	18	1.2	NS	NS	NS	NS	0.9
			Supine	3D Conformal with FIF	Static tangents	MV photons	No	No	Breast	50	25	18	5.7	NS	NS	NS	NS	9.8
			Supine	3D Conformal with FIF	Static tangents	MV photons	No	No	Breast	50	25	18	5.7	NS	NS	NS	NS	9.8
			Supine	3D Conformal with FIF	Static tangents	MV photons	No	No	Breast	50	25	18	5.7	NS	NS	NS	NS	9.8
			Prone	3D Conformal with FIF	Static tangents	MV photons	No	No	Breast	50	25	18	1.1	NS	NS	NS	NS	0.5
			Prone	3D Conformal with FIF	Static tangents	MV photons	No	No	Breast	50	25	18	1.1	NS	NS	NS	NS	0.5
			Prone	3D Conformal with FIF	Static tangents	MV photons	No	No	Breast	50	25	18	1.1	NS	NS	NS	NS	0.5
			Supine	3D IMRT	Oblique fields	MV photons	No	No	Breast	50	25	18	5.1	NS	NS	NS	NS	7.6
			Supine	3D IMRT	Oblique fields	MV photons	No	No	Breast	50	25	18	5.1	NS	NS	NS	NS	7.6
			Supine	3D IMRT	Oblique fields	MV photons	No	No	Breast	50	25	18	5.1	NS	NS	NS	NS	7.6
			Prone	3D IMRT	Oblique fields	MV photons	No	No	Breast	50	25	18	0.9	NS	NS	NS	NS	0.2
			Prone	3D IMRT	Oblique fields	MV photons	No	No	Breast	50	25	18	0.9	NS	NS	NS	NS	0.2
			Prone	3D IMRT	Oblique fields	MV photons	No	No	Breast	50	25	18	0.9	NS	NS	NS	NS	0.2
Mulliez RO	2015	Belgium	Supine	3D IMRT	Static tangents	Low energy MV photons	No	No	Breast	40.05	15	50	5.5	NS	NS	NS	NS	NS
			Supine	3D IMRT	Static tangents	Low energy MV photons	Yes	No	Breast	40.05	15	50	5.0	NS	NS	NS	NS	NS
			Supine	3D IMRT	Static tangents	Low energy MV photons	Yes	No	Breast	40.05	15	50	5.0	NS	NS	NS	NS	NS
			Supine	3D IMRT	Static tangents	Low energy MV photons	Yes	No	Breast	40.05	15	50	5.0	NS	NS	NS	NS	NS
			Prone	3D IMRT	Static tangents	Low energy MV photons	No	No	Breast	40.05	15	50	0.9	NS	NS	NS	NS	NS
			Prone	3D IMRT	Static tangents	Low energy MV photons	No	No	Breast	40.05	15	50	0.9	NS	NS	NS	NS	NS
			Prone	3D IMRT	Static tangents	Low energy MV photons	No	No	Breast	40.05	15	50	0.9	NS	NS	NS	NS	NS
			Prone	3D IMRT	Static tangents	Low energy MV photons	Yes	Yes	Breast	40.05	15	50	0.9	NS	NS	NS	NS	NS
			Prone	3D IMRT	Static tangents	Low energy MV photons	Yes	Yes	Breast	40.05	15	50	0.9	NS	NS	NS	NS	NS
			Prone	3D IMRT	Static tangents	Low energy MV photons	Yes	Yes	Breast	40.05	15	50	0.9	NS	NS	NS	NS	NS
Murofushi	2015	Japan	Supine	3D Conformal	Tangents plus boost/direct fields	Low energy MV photons	No	Yes	Breast; +/- SCF; +/- axilla; +/- IMC	50	25	948	NS	NS	NS	NS	NS	NS
			Supine	3D Conformal	Tangents plus boost/direct fields	Low energy MV photons	No	Yes	Chest wall; +/- SCF; +/- axilla; +/- IMC	50	25	228	NS	NS	NS	NS	NS	NS
			Supine	3D Conformal	Tangents plus boost/direct fields	Low energy MV photons	No	Yes	Chest wall; +/- SCF; +/- axilla; +/- IMC	50	25	228	NS	NS	NS	NS	NS	NS
			Supine	3D Conformal	Tangents plus boost/direct fields	Low energy MV photons	No	Yes	Chest wall; +/- SCF; +/- axilla; +/- IMC	50	25	228	NS	NS	NS	NS	NS	NS
Mydin	2010	Canada	Supine	3D Conformal plan	Oblique fields	MV photons	No	Yes	Partial breast	38.5	10	20	2.3	0.4-4.6	NS	NS	NS	NS
			Supine	3D Conformal plan	Static tangents plus electron fields	Mixed photons electrons	No	No	Partial breast	38.5	10	20	5.0	1.5-9.6	NS	NS	NS	NS
			Supine	3D Conformal plan	Static tangents plus electron fields	Mixed photons electrons	No	No	Partial breast	38.5	10	20	5.0	1.5-9.6	NS	NS	NS	NS
			Supine	3D Conformal plan	Static tangents plus electron fields	Mixed photons electrons	No	No	Partial breast	38.5	10	20	5.0	1.5-9.6	NS	NS	NS	NS
Nagar	2014	USA	Supine	3D Conformal	Static tangents	MV photons	No	NS	Breast	50.4	28	30	5.4	0.2-12.7	NS	NS	19.0	10.0
			Supine	3D Conformal	Static tangents	MV photons	No	No	Breast; axilla	50.4	28	30	9.5	4.8-18.4	NS	NS	32.0	18.0
			Supine	3D Conformal	Static tangents	MV photons	No	No	Breast; axilla	50.4	28	30	9.5	4.8-18.4	NS	NS	32.0	18.0
			Supine	3D Conformal	Static tangents	MV photons	No	No	Breast; axilla	50.4	28	30	9.5	4.8-18.4	NS	NS	32.0	18.0
Nakamura	2014	Japan	Supine	3D Conformal plan	Static tangents	Low energy MV phtons	No	NS	Breast	50	25	20	NS	NS	NS	5.2	NS	NS

Table E3 continued on the next page.

Table E3 continued from the previous page.

Author	Year	Country	Description of regimens*									No. of CT plans	Lung dose measures					
			Patient position	Treatment planning	Field type	Beam energy & modality	Breath. adapt.	RT plan delivered	Target(s)	Prescr. dose	No. of fraction		MLD_{ipsi} (Gy)		MLD_{cont} (Gy)	MLD_{whole} (Gy)	$V5_{ipsi}$ (%)	$V20_{ipsi}$ (%)
													Avg	Range				
			Supine	3D Conformal plan with FIF	Static tangents	Low energy MV phtons	No	NS	Breast	50	25	20	NS	NS	NS	4.8	NS	NS
			Supine	3D Conformal plan with FIF	Static tangents	Low energy MV phtons	No	NS	Breast	50	25	20	NS	NS	NS	4.8	NS	NS
			Supine	3D Conformal plan with FIF	Static tangents	Low energy MV phtons	No	NS	Breast	50	25	20	NS	NS	NS	4.8	NS	NS
			Supine	3D IMRT	Static tangents	Low energy MV phtons	No	NS	Breast	50	25	20	NS	NS	NS	4.7	NS	NS
			Supine	3D IMRT	Static tangents	Low energy MV phtons	No	NS	Breast	50	25	20	NS	NS	NS	4.7	NS	NS
			Supine	3D IMRT	Static tangents	Low energy MV phtons	No	NS	Breast	50	25	20	NS	NS	NS	4.7	NS	NS
			Supine	3D IMRT	Static tangents	Low energy MV phtons	No	NS	Breast	50	25	20	NS	NS	NS	3.6	NS	NS
			Supine	3D IMRT	Static tangents	Low energy MV phtons	No	NS	Breast	50	25	20	NS	NS	NS	3.6	NS	NS
			Supine	3D IMRT	Static tangents	Low energy MV phtons	No	NS	Breast	50	25	20	NS	NS	NS	3.6	NS	NS
Ng	2011	USA	Supine	3D Conformal plan	Static tangents	Low energy MV photons	No	NS	Breast	50	25	15	6.5	NS	NS	NS	NS	NS
			Prone	3D Conformal plan	Static tangents	Low energy MV photons	No	NS	Breast	50	25	15	0.5	NS	NS	NS	NS	NS
			Prone	3D Conformal plan	Static tangents	Low energy MV photons	No	NS	Breast	50	25	15	0.5	NS	NS	NS	NS	NS
			Prone	3D Conformal plan	Static tangents	Low energy MV photons	No	NS	Breast	50	25	15	0.5	NS	NS	NS	NS	NS
			Supine	3D Conformal plan	Static tangents	Low energy MV photons	No	NS	Breast	42.56	16	15	5.5	NS	NS	NS	NS	NS
			Supine	3D Conformal plan	Static tangents	Low energy MV photons	No	NS	Breast	42.56	16	15	5.5	NS	NS	NS	NS	NS
			Supine	3D Conformal plan	Static tangents	Low energy MV photons	No	NS	Breast	42.56	16	15	5.5	NS	NS	NS	NS	NS
			Prone	3D Conformal plan	Static tangents	Low energy MV photons	No	NS	Breast	42.56	16	15	0.5	NS	NS	NS	NS	NS
			Prone	3D Conformal plan	Static tangents	Low energy MV photons	No	NS	Breast	42.56	16	15	0.5	NS	NS	NS	NS	NS
			Prone	3D Conformal plan	Static tangents	Low energy MV photons	No	NS	Breast	42.56	16	15	0.5	NS	NS	NS	NS	NS
Nichols	2014	USA	Supine	3D IMRT	Rotational fields	Low energy MV phtons	No	NS	Chest wall; IMC; axilla; SCF	50.4	28	15	NS	NS	NS	NS	96.9	32.3
			Supine	3D IMRT	Rotational fields	Low energy MV phtons	No	NS	Chest wall; IMC; axilla; SCF	50.4	28	15	NS	NS	NS	NS	99.3	29.9
			Supine	3D IMRT	Rotational fields	Low energy MV phtons	No	NS	Chest wall; IMC; axilla; SCF	50.4	28	15	NS	NS	NS	NS	99.3	29.9
			Supine	3D IMRT	Rotational fields	Low energy MV phtons	No	NS	Chest wall; IMC; axilla; SCF	50.4	28	15	NS	NS	NS	NS	99.3	29.9
Nicolini	2011	Switzerland	Supine	3D IMRT	Rotational fields	Low energy MV photons	No	Yes	Breast	50	25	6	11.0	10.5-11.6	3.3	NS	NS	14.1
			Supine	3D IMRT	Rotational fields	Low energy MV photons	No	No	Breast	50	25	6	11.4	10.5-12.6	3.5	NS	NS	15.4
			Supine	3D IMRT	Rotational fields	Low energy MV photons	No	No	Breast	50	25	6	11.4	10.5-12.6	3.5	NS	NS	15.4
			Supine	3D IMRT	Rotational fields	Low energy MV photons	No	No	Breast	50	25	6	11.4	10.5-12.6	3.5	NS	NS	15.4
			Supine	3D IMRT	Rotational fields	Low energy MV photons	No	No	Breast	50	25	6	11.0	10.5-11.6	3.3	NS	NS	14.1
			Supine	3D IMRT	Rotational fields	Low energy MV photons	No	No	Breast	50	25	6	11.0	10.5-11.6	3.3	NS	NS	14.1
			Supine	3D IMRT	Rotational fields	Low energy MV photons	No	No	Breast	50	25	6	11.0	10.5-11.6	3.3	NS	NS	14.1
			Supine	3D IMRT	Rotational fields	Low energy MV photons	No	No	Breast	50	25	6	11.4	10.5-12.6	3.4	NS	NS	15.4
			Supine	3D IMRT	Rotational fields	Low energy MV photons	No	No	Breast	50	25	6	11.4	10.5-12.6	3.4	NS	NS	15.4
			Supine	3D IMRT	Rotational fields	Low energy MV photons	No	No	Breast	50	25	6	11.4	10.5-12.6	3.4	NS	NS	15.4
			Supine	3D IMRT	Rotational fields	Low energy MV photons	No	No	Breast	50	25	6	11.0	10.4-11.6	3.2	NS	NS	14.0
			Supine	3D IMRT	Rotational fields	Low energy MV photons	No	No	Breast	50	25	6	11.0	10.4-11.6	3.2	NS	NS	14.0
			Supine	3D IMRT	Rotational fields	Low energy MV photons	No	No	Breast	50	25	6	11.0	10.4-11.6	3.2	NS	NS	14.0
			Supine	3D IMRT	Rotational fields	Low energy MV photons	No	No	Breast	50	25	6	11.4	10.4-12.6	3.7	NS	NS	15.4
			Supine	3D IMRT	Rotational fields	Low energy MV photons	No	No	Breast	50	25	6	11.4	10.4-12.6	3.7	NS	NS	15.4
			Supine	3D IMRT	Rotational fields	Low energy MV photons	No	No	Breast	50	25	6	11.4	10.4-12.6	3.7	NS	NS	15.4
Nitsche	2014	Germany	Supine	3D Conformal plan	Static tangents	Mixed energy MV photons	No	NS	Breast	50.4	28	51	7.9	NS	NS	4.2	NS	NS
			Supine	3D Conformal plan	Static tangents	Mixed energy MV photons	No	NS	Breast; axilla	50.4	28	51	11.2	NS	NS	5.9	NS	NS
			Supine	3D Conformal plan	Static tangents	Mixed energy MV photons	No	NS	Breast; axilla	50.4	28	51	11.2	NS	NS	5.9	NS	NS
			Supine	3D Conformal plan	Static tangents	Mixed energy MV photons	No	NS	Breast; axilla	50.4	28	51	11.2	NS	NS	5.9	NS	NS

Table E3 continued on the next page.

Table E3 continued from the previous page.

Author	Year	Country	Patient position	Treatment planning	Field type	Beam energy & modality	Breath. adapt.	RT plan delivered	Target(s)	Prescr. dose	No. of fraction	No. of CT plans	MLD_{ipsi} (Gy) Avg	MLD_{ipsi} (Gy) Range	MLD_{cont} (Gy)	MLD_{whole} (Gy)	$V5_{ipsi}$ (%)	$V20_{ipsi}$ (%)
						Description of regimens*								Lung dose measures				
Onal	2012	Turkey	Supine	3D Conformal	Static tangents	Low energy MV photons	No	NS	Breast	50	25	30	8.5	NS	NS	NS	NS	NS
			Supine	3D Conformal	Static tangents	Low energy MV photons	No	NS	Breast	50	25	30	8.8	NS	NS	NS	NS	NS
			Supine	3D Conformal	Static tangents	Low energy MV photons	No	NS	Breast	50	25	30	8.8	NS	NS	NS	NS	NS
			Supine	3D Conformal	Static tangents	Low energy MV photons	No	NS	Breast	50	25	30	8.8	NS	NS	NS	NS	NS
			Supine	3D Conformal with FIF	Static tangents	Low energy MV photons	No	NS	Breast	50	25	30	8.4	NS	NS	NS	NS	NS
			Supine	3D Conformal with FIF	Static tangents	Low energy MV photons	No	NS	Breast	50	25	30	8.4	NS	NS	NS	NS	NS
			Supine	3D Conformal with FIF	Static tangents	Low energy MV photons	No	NS	Breast	50	25	30	8.4	NS	NS	NS	NS	NS
Opp	2013	USA	Supine	3D Conformal	Direct fields	Electrons	No	No	Chest wall; axilla	50	25	21	NS	NS	1.0	8.8	NS	NS
			Supine	3D IMRT	Oblique fields	MV photons	No	No	Chest wall; axilla	50	25	21	NS	NS	3.6	8.6	NS	NS
			Supine	3D IMRT	Oblique fields	MV photons	No	No	Chest wall; axilla	50	25	21	NS	NS	3.6	8.6	NS	NS
			Supine	3D IMRT	Oblique fields	MV photons	No	No	Chest wall; axilla	50	25	21	NS	NS	3.6	8.6	NS	NS
			Supine	3D Conformal	Static tangents	MV photons	No	No	Chest wall; axilla	50	25	21	NS	NS	0.5	8.3	NS	NS
			Supine	3D Conformal	Static tangents	MV photons	No	No	Chest wall; axilla	50	25	21	NS	NS	0.5	8.3	NS	NS
			Supine	3D Conformal	Static tangents	MV photons	No	No	Chest wall; axilla	50	25	21	NS	NS	0.5	8.3	NS	NS
			Supine	3D Conformal with FIF	Static tangents	MV photons	No	No	Chest wall; axilla	50	25	21	NS	NS	0.3	7.3	NS	NS
			Supine	3D Conformal with FIF	Static tangents	MV photons	No	No	Chest wall; axilla	50	25	21	NS	NS	0.3	7.3	NS	NS
			Supine	3D Conformal with FIF	Static tangents	MV photons	No	No	Chest wall; axilla	50	25	21	NS	NS	0.3	7.3	NS	NS
Osei	2015	Canada	Supine	3D Conformal with FIF	Static tangents	MV photons	No	NS	Breast	42.5	16	431	NS	NS	NS	NS	18.0	8.4
			Supine	3D Conformal with FIF	Static tangents	MV photons	No	NS	Breast	50	25	45	NS	NS	NS	NS	21.6	8.9
			Supine	3D Conformal with FIF	Static tangents	MV photons	No	NS	Breast	50	25	45	NS	NS	NS	NS	21.6	8.9
			Supine	3D Conformal with FIF	Static tangents	MV photons	No	NS	Breast	50	25	45	NS	NS	NS	NS	21.6	8.9
			Supine	3D Conformal with FIF	Static tangents	MV photons	Yes	NS	Breast	42.5	16	139	NS	NS	NS	NS	18.6	8.1
			Supine	3D Conformal with FIF	Static tangents	MV photons	Yes	NS	Breast	42.5	16	139	NS	NS	NS	NS	18.6	8.1
			Supine	3D Conformal with FIF	Static tangents	MV photons	Yes	NS	Breast	42.5	16	139	NS	NS	NS	NS	18.6	8.1
			Supine	3D Conformal with FIF	Static tangents	MV photons	Yes	NS	Breast	50	25	8	NS	NS	NS	NS	20.7	8.4
			Supine	3D Conformal with FIF	Static tangents	MV photons	Yes	NS	Breast	50	25	8	NS	NS	NS	NS	20.7	8.4
			Supine	3D Conformal with FIF	Static tangents	MV photons	Yes	NS	Breast	50	25	8	NS	NS	NS	NS	20.7	8.4
Osman	2014	Netherlands	Supine	3D Conformal plan with FIF	Static tangents	Mixed energy MV photons	No	NS	Breast; IMC; SCF	42.6	16	13	19.0	13.3-23.8	0.4	8.7	69.4	44.2
			Supine	3D Conformal plan with FIF	Static tangents	Mixed energy MV photons	Yes	NS	Breast; IMC; SCF	42.6	16	13	17.1	13-21.9	0.4	7.8	65.5	38.7
			Supine	3D Conformal plan with FIF	Static tangents	Mixed energy MV photons	Yes	NS	Breast; IMC; SCF	42.6	16	13	17.1	13-21.9	0.4	7.8	65.5	38.7
			Supine	3D Conformal plan with FIF	Static tangents	Mixed energy MV photons	Yes	NS	Breast; IMC; SCF	42.6	16	13	17.1	13-21.9	0.4	7.8	65.5	38.7
			Supine	3D IMRT	Rotational fields	Low energy MV phtons	No	No	Breast; IMC; SCF	42.6	16	13	14.0	8.4-21.3	3.4	8.3	67.5	27.9
			Supine	3D IMRT	Rotational fields	Low energy MV phtons	No	No	Breast; IMC; SCF	42.6	16	13	14.0	8.4-21.3	3.4	8.3	67.5	27.9
			Supine	3D IMRT	Rotational fields	Low energy MV phtons	No	No	Breast; IMC; SCF	42.6	16	13	14.0	8.4-21.3	3.4	8.3	67.5	27.9
			Supine	3D IMRT	Rotational fields	Low energy MV phtons	Yes	No	Breast; IMC; SCF	42.6	16	13	13.3	9.6-18.8	2.6	7.5	66.2	26.5
			Supine	3D IMRT	Rotational fields	Low energy MV phtons	Yes	No	Breast; IMC; SCF	42.6	16	13	13.3	9.6-18.8	2.6	7.5	66.2	26.5
			Supine	3D IMRT	Rotational fields	Low energy MV phtons	Yes	No	Breast; IMC; SCF	42.6	16	13	13.3	9.6-18.8	2.6	7.5	66.2	26.5
Palta	2012	USA	Prone	3D IMRT	Oblique fields	Mixed energy MV photons	No	No	Partial breast	15	1	17	0.3	0.1-0.9	NS	NS	NS	NS
Pasler	2015	Austria	Supine	3D IMRT	Rotational fields	Low energy MV phtons	No	NS	Breast; SCF	50.4	28	10	13.5	NS	1.9	NS	NS	26.5
			Supine	3D IMRT	Rotational fields	Low energy MV phtons	No	NS	Breast; SCF	50.4	28	10	13.6	NS	0.7	NS	NS	26.6
			Supine	3D IMRT	Rotational fields	Low energy MV phtons	No	NS	Breast; SCF	50.4	28	10	13.6	NS	0.7	NS	NS	26.6
			Supine	3D IMRT	Rotational fields	Low energy MV phtons	No	NS	Breast; SCF	50.4	28	10	13.6	NS	0.7	NS	NS	26.6

Table E3 continued on the next page.

Table E3 continued from the previous page.

Author	Year	Country	Patient position	Treatment planning	Field type	Beam energy & modality	Breath. adapt.	RT plan delivered	Target(s)	Prescr. dose	No. of fraction	No. of CT plans	MLD$_{ipsi}$ (Gy) Avg	MLD$_{ipsi}$ (Gy) Range	MLD$_{cont}$ (Gy)	MLD$_{whole}$ (Gy)	V5$_{ipsi}$ (%)	V20$_{ipsi}$ (%)
			Description of regimens*										Lung dose measures					
Pasler Georg	2013	Austria	Supine	3D IMRT	Oblique fields	Low energy MV photons	No	NS	Breast; SCF; infraclav	50.4	28	10	14.6	NS	4.2	NS	NS	23.9
			Supine	3D IMRT	Oblique fields	High energy MV photons	No	NS	Breast; SCF; infraclav	50.4	28	10	14.7	NS	4.4	NS	NS	25.6
			Supine	3D IMRT	Oblique fields	High energy MV photons	No	NS	Breast; SCF; infraclav	50.4	28	10	14.7	NS	4.4	NS	NS	25.6
			Supine	3D IMRT	Oblique fields	High energy MV photons	No	NS	Breast; SCF; infraclav	50.4	28	10	14.7	NS	4.4	NS	NS	25.6
			Supine	3D IMRT	Oblique fields	High energy MV photons	No	NS	Breast; SCF; infraclav	50.4	28	10	15.0	NS	4.5	NS	NS	26.6
			Supine	3D IMRT	Oblique fields	High energy MV photons	No	NS	Breast; SCF; infraclav	50.4	28	10	15.0	NS	4.5	NS	NS	26.6
			Supine	3D IMRT	Oblique fields	High energy MV photons	No	NS	Breast; SCF; infraclav	50.4	28	10	15.0	NS	4.5	NS	NS	26.6
			Supine	3D IMRT	Rotational fields	Low energy MV photons	No	NS	Breast; SCF; infraclav	50.4	28	10	14.6	NS	3.9	NS	NS	26.8
			Supine	3D IMRT	Rotational fields	Low energy MV photons	No	NS	Breast; SCF; infraclav	50.4	28	10	14.6	NS	3.9	NS	NS	26.8
			Supine	3D IMRT	Rotational fields	Low energy MV photons	No	NS	Breast; SCF; infraclav	50.4	28	10	14.6	NS	3.9	NS	NS	26.8
			Supine	3D IMRT	Rotational fields	High energy MV photons	No	NS	Breast; SCF; infraclav	50.4	28	10	14.8	NS	3.4	NS	NS	27.7
			Supine	3D IMRT	Rotational fields	High energy MV photons	No	NS	Breast; SCF; infraclav	50.4	28	10	14.8	NS	3.4	NS	NS	27.7
			Supine	3D IMRT	Rotational fields	High energy MV photons	No	NS	Breast; SCF; infraclav	50.4	28	10	14.8	NS	3.4	NS	NS	27.7
			Supine	3D IMRT	Rotational fields	High energy MV photons	No	NS	Breast; SCF; infraclav	50.4	28	10	14.8	NS	4.5	NS	NS	27.0
			Supine	3D IMRT	Rotational fields	High energy MV photons	No	NS	Breast; SCF; infraclav	50.4	28	10	14.8	NS	4.5	NS	NS	27.0
			Supine	3D IMRT	Rotational fields	High energy MV photons	No	NS	Breast; SCF; infraclav	50.4	28	10	14.8	NS	4.5	NS	NS	27.0
Peters	2014	Switzerland	Supine	3D IMRT	Oblique fields	Low energy MV photons	No	No	Breast	50	25	6	14.6	12.2-17.6	6.3	10.3	88.7	20.7
			Supine	3D IMRT	Rotational fields	Low energy MV photons	No	No	Breast	50	25	6	13.0	11-14.7	5.9	9.3	81.2	19.2
			Supine	3D IMRT	Rotational fields	Low energy MV photons	No	No	Breast	50	25	6	13.0	11-14.7	5.9	9.3	81.2	19.2
			Supine	3D IMRT	Rotational fields	Low energy MV photons	No	No	Breast	50	25	6	13.0	11-14.7	5.9	9.3	81.2	19.2
			Supine	3D IMRT	Rotational fields	Low energy MV photons	No	No	Breast	50	25	6	12.6	11.8-13.5	6.5	9.4	81.2	18.3
			Supine	3D IMRT	Rotational fields	Low energy MV photons	No	No	Breast	50	25	6	12.6	11.8-13.5	6.5	9.4	81.2	18.3
			Supine	3D IMRT	Rotational fields	Low energy MV photons	No	No	Breast	50	25	6	12.6	11.8-13.5	6.5	9.4	81.2	18.3
			Supine	3D IMRT	Oblique fields	Low energy MV photons	No	No	Breast	50	25	4	14.6	12.2-17.6	6.3	10.3	88.7	20.7
			Supine	3D IMRT	Oblique fields	Low energy MV photons	No	No	Breast	50	25	4	14.6	12.2-17.6	6.3	10.3	88.7	20.7
			Supine	3D IMRT	Oblique fields	Low energy MV photons	No	No	Breast	50	25	4	14.6	12.2-17.6	6.3	10.3	88.7	20.7
			Supine	3D IMRT	Rotational fields	Low energy MV photons	No	No	Breast	50	25	4	13.0	11-14.7	5.9	9.3	81.2	19.2
			Supine	3D IMRT	Rotational fields	Low energy MV photons	No	No	Breast	50	25	4	13.0	11-14.7	5.9	9.3	81.2	19.2
			Supine	3D IMRT	Rotational fields	Low energy MV photons	No	No	Breast	50	25	4	13.0	11-14.7	5.9	9.3	81.2	19.2
			Supine	3D IMRT	Rotational fields	Low energy MV photons	No	No	Breast	50	25	4	12.6	11.8-13.5	6.5	9.4	81.2	18.3
			Supine	3D IMRT	Rotational fields	Low energy MV photons	No	No	Breast	50	25	4	12.6	11.8-13.5	6.5	9.4	81.2	18.3
			Supine	3D IMRT	Rotational fields	Low energy MV photons	No	No	Breast	50	25	4	12.6	11.8-13.5	6.5	9.4	81.2	18.3
Peulen	2010	Netherlands	Supine	3D Conformal with FIF	Static tangents	Low energy MV photons	Yes	Yes	Breast	50	25	21	5.8	3.1-9.8	0.2	3.2	NS	NS
			Supine	3D Conformal with FIF	Tangents plus boost/direct fields	Low energy MV photons	Yes	Yes	Breast	50	25	39	7.7	2.9-10.7	0.3	3.8	NS	NS
			Supine	3D Conformal with FIF	Tangents plus boost/direct fields	Low energy MV photons	Yes	Yes	Breast	50	25	39	7.7	2.9-10.7	0.3	3.8	NS	NS
			Supine	3D Conformal with FIF	Tangents plus boost/direct fields	Low energy MV photons	Yes	Yes	Breast	50	25	39	7.7	2.9-10.7	0.3	3.8	NS	NS
			Supine	3D Conformal with FIF	Tangents plus boost/direct fields	Low energy MV photons	No	Yes	Breast	50	25	14	7.7	2.9-10.7	0.3	3.8	NS	NS
			Supine	3D Conformal with FIF	Tangents plus boost/direct fields	Low energy MV photons	No	Yes	Breast	50	25	14	7.7	2.9-10.7	0.3	3.8	NS	NS
			Supine	3D Conformal with FIF	Tangents plus boost/direct fields	Low energy MV photons	No	Yes	Breast	50	25	14	7.7	2.9-10.7	0.3	3.8	NS	NS
Pili	2011	Italy	Supine	3D Conformal	Static tangents	Low energy MV photons	No	NS	Breast	40-47.5	NS	30	8.0	NS	NS	NS	NS	NS
			Supine	3D Conformal with FIF	Static tangents	Low energy MV photons	No	NS	Breast	40-47.5	NS	30	4.6	NS	NS	NS	NS	NS

Table E3 continued on the next page.

Table E3 continued from the previous page.

Author	Year	Country	Description of regimens* Patient position	Treatment planning	Field type	Beam energy & modality	Breath. adapt.	RT plan delivered	Target(s)	Prescr. dose	No. of fraction	No. of CT plans	Lung dose measures MLD$_{ipsi}$ (Gy) Avg	MLD$_{ipsi}$ (Gy) Range	MLD$_{cont}$ (Gy)	MLD$_{whole}$ (Gy)	V5$_{ipsi}$ (%)	V20$_{ipsi}$ (%)
			Supine	3D Conformal with FIF	Static tangents	Low energy MV photons	No	NS	Breast	40-47.5	NS	30	4.6	NS	NS	NS	NS	NS
			Supine	3D Conformal with FIF	Static tangents	Low energy MV photons	No	NS	Breast	40-47.5	NS	30	4.6	NS	NS	NS	NS	NS
Popescu	2010	Canada	Supine	3D Conformal	Wide tangents plus boost/direct fields	Mixed energy MV photons	No	No	Breast; SCF; axilla; IMC	50	25	5	18.1	14.2-22.3	0.8	NS	46.8	37.3
			Supine	3D IMRT	Direct fields	Low energy MV photons	No	Yes	Breast; SCF; axilla; IMC	50	25	5	13.1	12.8-13.5	5.5	NS	91.9	17.3
			Supine	3D IMRT	Direct fields	Low energy MV photons	No	Yes	Breast; SCF; axilla; IMC	50	25	5	13.1	12.8-13.5	5.5	NS	91.9	17.3
			Supine	3D IMRT	Direct fields	Low energy MV photons	No	Yes	Breast; SCF; axilla; IMC	50	25	5	13.1	12.8-13.5	5.5	NS	91.9	17.3
			Supine	3D IMRT	Rotational fields	MV photons	No	No	Breast; SCF; axilla; IMC	50	25	5	11.6	11.2-12.3	2.9	NS	70.2	16.9
			Supine	3D IMRT	Rotational fields	MV photons	No	No	Breast; SCF; axilla; IMC	50	25	5	11.6	11.2-12.3	2.9	NS	70.2	16.9
			Supine	3D IMRT	Rotational fields	MV photons	No	No	Breast; SCF; axilla; IMC	50	25	5	11.6	11.2-12.3	2.9	NS	70.2	16.9
Qi	2014	USA	Supine	3D Conformal	Static tangents	MV photons	No	Yes	Breast	42.6	16	5	6.8	NS	0.2	NS	NS	14.2
			Supine	3D IMRT	Rotational fields	Low energy MV photons	No	No	Breast	42.6	16	5	5.9	NS	1.6	NS	NS	3.6
			Supine	3D IMRT	Rotational fields	Low energy MV photons	No	No	Breast	42.6	16	5	5.9	NS	1.6	NS	NS	3.6
			Supine	3D IMRT	Rotational fields	Low energy MV photons	No	No	Breast	42.6	16	5	5.9	NS	1.6	NS	NS	3.6
			Supine	3D IMRT	Rotational fields	Low energy MV photons	No	No	Breast	42.6	16	5	2.9	NS	0.2	NS	NS	4.8
			Supine	3D IMRT	Rotational fields	Low energy MV photons	No	No	Breast	42.6	16	5	2.9	NS	0.2	NS	NS	4.8
			Supine	3D IMRT	Rotational fields	Low energy MV photons	No	No	Breast	42.6	16	5	2.9	NS	0.2	NS	NS	4.8
			Supine	3D IMRT	Static tangents	Low energy MV photons	No	No	Breast	42.6	16	5	5.2	NS	2.9	NS	NS	3.2
			Supine	3D IMRT	Static tangents	Low energy MV photons	No	No	Breast	42.6	16	5	5.2	NS	2.9	NS	NS	3.2
			Supine	3D IMRT	Static tangents	Low energy MV photons	No	No	Breast	42.6	16	5	5.2	NS	2.9	NS	NS	3.2
			Supine	3D IMRT	Oblique fields	MV photons	No	Yes	Breast or chest wall; +/- IMC; +/- SCF; +/- axilla	50	25	5	14.4	NS	2.8	NS	NS	32.2
			Supine	3D IMRT	Oblique fields	MV photons	No	Yes	Breast or chest wall; +/- IMC; +/- SCF; +/- axilla	50	25	5	14.4	NS	2.8	NS	NS	32.2
			Supine	3D IMRT	Oblique fields	MV photons	No	Yes	Breast or chest wall; +/- IMC; +/- SCF; +/- axilla	50	25	5	14.4	NS	2.8	NS	NS	32.2
			Supine	3D IMRT	Rotational fields	Low energy MV photons	No	No	Breast or chest wall; +/- IMC; +/- SCF; +/- axilla	50	25	5	11.4	NS	3.5	NS	NS	16.0
			Supine	3D IMRT	Rotational fields	Low energy MV photons	No	No	Breast or chest wall; +/- IMC; +/- SCF; +/- axilla	50	25	5	11.4	NS	3.5	NS	NS	16.0
			Supine	3D IMRT	Rotational fields	Low energy MV photons	No	No	Breast or chest wall; +/- IMC; +/- SCF; +/- axilla	50	25	5	11.4	NS	3.5	NS	NS	16.0
			Supine	3D IMRT	Rotational fields	Low energy MV photons	No	No	Breast or chest wall; +/- IMC; +/- SCF; +/- axilla	50	25	5	11.4	NS	0.6	NS	NS	22.6
			Supine	3D IMRT	Rotational fields	Low energy MV photons	No	No	Breast or chest wall; +/- IMC; +/- SCF; +/- axilla	50	25	5	11.4	NS	0.6	NS	NS	22.6

Table E3 continued on the next page.

Table E3 continued from the previous page.

Author	Year	Country	Description of regimens*									No. of CT plans	Lung dose measures					
			Patient position	Treatment planning	Field type	Beam energy & modality	Breath. adapt.	RT plan delivered	Target(s)	Prescr. dose	No. of fraction		MLD_{ipsi} (Gy)		MLD_{cont} (Gy)	MLD_{whole} (Gy)	$V5_{ipsi}$ (%)	$V20_{ipsi}$ (%)
													Avg	Range				
			Supine	3D IMRT	Rotational fields	Low energy MV photons	No	No	Breast or chest wall; +/- IMC; +/- SCF; +/- axilla	50	25	5	11.4	NS	0.6	NS	NS	22.6
			Supine	3D IMRT	Static tangents	Low energy MV photons	No	No	Breast or chest wall; +/- IMC; +/- SCF; +/- axilla	50	25	5	10.3	NS	4.8	NS	NS	10.0
			Supine	3D IMRT	Static tangents	Low energy MV photons	No	No	Breast or chest wall; +/- IMC; +/- SCF; +/- axilla	50	25	5	10.3	NS	4.8	NS	NS	10.0
			Supine	3D IMRT	Static tangents	Low energy MV photons	No	No	Breast or chest wall; +/- IMC; +/- SCF; +/- axilla	50	25	5	10.3	NS	4.8	NS	NS	10.0
Qiu MedDos	2014	China	Supine	3D Conformal	Oblique fields	MV photons	No	Yes	Partial breast	38.5	10	16	1.9	NS	0.8	NS	11.1	1.5
			Supine	3D IMRT	Oblique fields	MV photons	No	No	Partial breast	38.5	10	16	1.2	NS	0.2	NS	4.5	0.3
			Supine	3D IMRT	Oblique fields	MV photons	No	No	Partial breast	38.5	10	16	1.2	NS	0.2	NS	4.5	0.3
			Supine	3D IMRT	Oblique fields	MV photons	No	No	Partial breast	38.5	10	16	1.2	NS	0.2	NS	4.5	0.3
			Supine	3D IMRT	Rotational fields	MV photons	No	No	Partial breast	38.5	10	16	1.5	NS	0.4	NS	7.4	0.2
			Supine	3D IMRT	Rotational fields	MV photons	No	No	Partial breast	38.5	10	16	1.5	NS	0.4	NS	7.4	0.2
			Supine	3D IMRT	Rotational fields	MV photons	No	No	Partial breast	38.5	10	16	1.5	NS	0.4	NS	7.4	0.2
Qiu	2010	China	Supine	3D Conformal	Static tangents	Low energy MV photons	No	Yes	Partial breast	38.5	10	8	1.6	2.1-3.2	NS	NS	6.3	1.6
			Supine	3D IMRT	Rotational fields	Low energy MV photons	No	No	Partial breast	38.5	10	8	1.3	1.4-2.0	NS	NS	5.9	0.5
			Supine	3D IMRT	Rotational fields	Low energy MV photons	No	No	Partial breast	38.5	10	8	1.3	1.4-2.0	NS	NS	5.9	0.5
			Supine	3D IMRT	Rotational fields	Low energy MV photons	No	No	Partial breast	38.5	10	8	1.3	1.4-2.0	NS	NS	5.9	0.5
Qiu Biomed	2013	China	Supine	3D IMRT	Rotational fields	MV photons	No	No	Breast	46	23	10	6.1	NS	0.5	3.2	21.0	11.6
			Supine	3D Conformal with FIF	Static tangents	MV photons	No	No	Breast	46	23	10	9.0	NS	0.1	4.3	29.2	17.7
			Supine	3D Conformal with FIF	Static tangents	MV photons	No	No	Breast	46	23	10	9.0	NS	0.1	4.3	29.2	17.7
			Supine	3D Conformal with FIF	Static tangents	MV photons	No	No	Breast	46	23	10	9.0	NS	0.1	4.3	29.2	17.7
Reardon	2013	USA	Supine	3D Conformal with FIF	Static tangents	MV photons	Yes	Yes	Breast	45	25	10	NS	NS	NS	2.4	NS	NS
			Supine	3D IMRT	Static tangents	MV photons	No	No	Breast	45	25	10	NS	NS	NS	2.1	NS	NS
			Supine	3D IMRT	Static tangents	MV photons	No	No	Breast	45	25	10	NS	NS	NS	2.1	NS	NS
			Supine	3D IMRT	Static tangents	MV photons	No	No	Breast	45	25	10	NS	NS	NS	2.1	NS	NS
Register	2015	USA	Supine	3D Conformal with FIF	Tangents plus boost/direct fields	MV photons	No	NS	Breast; +/- SCF	40-50	16-25	44	9.8	NS	NS	NS	39.0	15.8
			Supine	3D Conformal with FIF	Tangents plus boost/direct fields	MV photons	Yes	NS	Breast; +/- SCF	40-50	16-25	47	8.2	NS	NS	NS	34.3	12.7
			Supine	3D Conformal with FIF	Tangents plus boost/direct fields	MV photons	Yes	NS	Breast; +/- SCF	40-50	16-25	47	8.2	NS	NS	NS	34.3	12.7
			Supine	3D Conformal with FIF	Tangents plus boost/direct fields	MV photons	Yes	NS	Breast; +/- SCF	40-50	16-25	47	8.2	NS	NS	NS	34.3	12.7
			Supine	3D Conformal with FIF	Tangents plus boost/direct fields	MV photons	No	NS	Chest wall; +/- SCF	45-50	25	17	14.8	NS	NS	NS	53.9	28.0
			Supine	3D Conformal with FIF	Tangents plus boost/direct fields	MV photons	No	NS	Chest wall; +/- SCF	45-50	25	17	14.8	NS	NS	NS	53.9	28.0
			Supine	3D Conformal with FIF	Tangents plus boost/direct fields	MV photons	No	NS	Chest wall; +/- SCF	45-50	25	17	14.8	NS	NS	NS	53.9	28.0

Table E3 continued on the next page.

Table E3 continued from the previous page.

Author	Year	Country	Description of regimens* Patient position	Treatment planning	Field type	Beam energy & modality	Breath. adapt.	RT plan delivered	Target(s)	Prescr. dose	No. of fraction	No. of CT plans	Lung dose measures MLD_{ipsi} (Gy) Avg	Range	MLD_{cont} (Gy)	MLD_{whole} (Gy)	$V5_{ipsi}$ (%)	$V20_{ipsi}$ (%)
			Supine	3D Conformal with FIF	Tangents plus boost/direct fields	MV photons	Yes	NS	Chest wall; +/- SCF	45-50	25	17	11.9	NS	NS	NS	47.1	21.0
			Supine	3D Conformal with FIF	Tangents plus boost/direct fields	MV photons	Yes	NS	Chest wall; +/- SCF	45-50	25	17	11.9	NS	NS	NS	47.1	21.0
			Supine	3D Conformal with FIF	Tangents plus boost/direct fields	MV photons	Yes	NS	Chest wall; +/- SCF	45-50	25	17	11.9	NS	NS	NS	47.1	21.0
Rudat	2011	Saudi Arabia	Supine	3D Conformal	Static tangents	Low energy MV photons	No	NS	Chest wall	50	25	20	11.3	NS	NS	NS	NS	NS
			Supine	3D Conformal with FIF	Static tangents	Low energy MV photons	No	NS	Chest wall	50	25	20	14.4	NS	NS	NS	NS	NS
			Supine	3D Conformal with FIF	Static tangents	Low energy MV photons	No	NS	Chest wall	50	25	20	14.4	NS	NS	NS	NS	NS
			Supine	3D Conformal with FIF	Static tangents	Low energy MV photons	No	NS	Chest wall	50	25	20	14.4	NS	NS	NS	NS	NS
Rudra	2014	USA	Supine	3D Conformal	Static tangents plus electron field	Mixed photons & electrons	No	Yes	Breast or chest wall; axilla; SCF; IMC	46.8-50.4	28	14	22.9	NS	NS	11.7	69.3	44.1
			Supine	3D Conformal	Static tangents plus electron field	Mixed photons & electrons	No	Yes	Breast or chest wall; axilla; SCF; IMC	46.8-50.4	28	14	22.2	NS	NS	11.9	84.5	44.5
			Supine	3D Conformal	Static tangents plus electron field	Mixed photons & electrons	No	Yes	Breast or chest wall; axilla; SCF; IMC	46.8-50.4	28	14	22.2	NS	NS	11.9	84.5	44.5
			Supine	3D Conformal	Static tangents plus electron field	Mixed photons & electrons	No	Yes	Breast or chest wall; axilla; SCF; IMC	46.8-50.4	28	14	22.2	NS	NS	11.9	84.5	44.5
Sager	2012	Turkey	Supine	3D Conformal	NS	NS	No	No	Breast	50	25	25	11.6	NS	NS	5.4	28.4	21.1
			Supine	3D Conformal	NS	NS	Yes	Yes	Breast	50	25	25	8.9	NS	NS	4.3	21.9	15.5
			Supine	3D Conformal	NS	NS	Yes	Yes	Breast	50	25	25	8.9	NS	NS	4.3	21.9	15.5
			Supine	3D Conformal	NS	NS	Yes	Yes	Breast	50	25	25	8.9	NS	NS	4.3	21.9	15.5
Saha	2015	India	Supine	3D IMRT	Rotational fields	Low energy MV photons	No	Yes	Chest wall; SCF	40	15	1	11.4	NS	NS	NS	NS	NS
			Supine	3D IMRT	Rotational fields	Low energy MV photons	No	Yes	axilla	60	30	1	1.2	NS	NS	NS	NS	NS
			Supine	3D IMRT	Rotational fields	Low energy MV photons	No	Yes	axilla	60	30	1	1.2	NS	NS	NS	NS	NS
			Supine	3D IMRT	Rotational fields	Low energy MV photons	No	Yes	axilla	60	30	1	1.2	NS	NS	NS	NS	NS
			Supine	3D IMRT	Rotational fields	Low energy MV photons	No	Yes	Breast; SCF; IMC	40	15	1	14.7	NS	NS	NS	NS	NS
			Supine	3D IMRT	Rotational fields	Low energy MV photons	No	Yes	Breast; SCF; IMC	40	15	1	14.7	NS	NS	NS	NS	NS
			Supine	3D IMRT	Rotational fields	Low energy MV photons	No	Yes	Breast; SCF; IMC	40	15	1	14.7	NS	NS	NS	NS	NS
Sakumi	2012	Japan	Supine	3D IMRT	Rotational fields	MV photons	No	No	Breast or chest wall; SCF; IMC	50	25	5	12.7	11.2-13.8	4.0	NS	74.6	18.9
Salem	2015	Jordan	Supine	3D Conformal with FIF	Static tangents	Mixed photons & electrons	No	NS	Chest wall; SCF	50	25	13	NS	NS	NS	6.0	NS	NS
			Supine	3D Conformal with FIF	Static tangents	Mixed photons & electrons	No	NS	Chest wall; SCF	50	25	13	NS	NS	NS	6.8	NS	NS
			Supine	3D Conformal with FIF	Static tangents	Mixed photons & electrons	No	NS	Chest wall; SCF	50	25	13	NS	NS	NS	6.8	NS	NS
			Supine	3D Conformal with FIF	Static tangents	Mixed photons & electrons	No	NS	Chest wall; SCF	50	25	13	NS	NS	NS	6.8	NS	NS
			Supine	3D Conformal with FIF	Static tangents	Low energy MV photons	No	NS	Chest wall; SCF	50	25	13	NS	NS	NS	7.5	NS	NS
			Supine	3D Conformal with FIF	Static tangents	Low energy MV photons	No	NS	Chest wall; SCF	50	25	13	NS	NS	NS	7.5	NS	NS
			Supine	3D Conformal with FIF	Static tangents	Low energy MV photons	No	NS	Chest wall; SCF	50	25	13	NS	NS	NS	7.5	NS	NS
Sasaoka	2011	Japan	Supine	3D Conformal	Static tangents	Low energy MV photons	No	Yes	Breast	50	25	10	8.8	NS	NS	NS	NS	14.9
			Supine	3D Conformal with FIF	Static tangents	Low energy MV photons	No	No	Breast	50	25	10	8.1	NS	NS	NS	NS	14.7

Table E3 continued on the next page.

Table E3 continued from the previous page.

Author	Year	Country	Description of regimens*										Lung dose measures					
			Patient position	Treatment planning	Field type	Beam energy & modality	Breath. adapt.	RT plan delivered	Target(s)	Prescr. dose	No. of fraction	No. of CT plans	MLD_{ipsi} (Gy)		MLD_{cont} (Gy)	MLD_{whole} (Gy)	$V5_{ipsi}$ (%)	$V20_{ipsi}$ (%)
													Avg	Range				
			Supine	3D Conformal with FIF	Static tangents	Low energy MV photons	No	No	Breast	50	25	10	8.1	NS	NS	NS	NS	14.7
			Supine	3D Conformal with FIF	Static tangents	Low energy MV photons	No	No	Breast	50	25	10	8.1	NS	NS	NS	NS	14.7
			Supine	3D Conformal	Static tangents	Low energy MV photons	No	Yes	Breast	50	25	10	8.8	NS	NS	NS	NS	14.9
			Supine	3D Conformal	Static tangents	Low energy MV photons	No	Yes	Breast	50	25	10	8.8	NS	NS	NS	NS	14.9
			Supine	3D Conformal	Static tangents	Low energy MV photons	No	Yes	Breast	50	25	10	8.8	NS	NS	NS	NS	14.9
			Supine	3D Conformal with FIF	Static tangents	Low energy MV photons	No	No	Breast	50	25	10	8.1	NS	NS	NS	NS	14.7
			Supine	3D Conformal with FIF	Static tangents	Low energy MV photons	No	No	Breast	50	25	10	8.1	NS	NS	NS	NS	14.7
			Supine	3D Conformal with FIF	Static tangents	Low energy MV photons	No	No	Breast	50	25	10	8.1	NS	NS	NS	NS	14.7
Sas-Korczynska	2010	Poland	Supine	3D Conformal	Static tangents plus electron field	Mixed photons & electrons	No	NS	Breast	42.5	17	10	5.6	0.3-50.1	NS	NS	NS	10.7
			Supine	3D IMRT	Static tangents plus electron field	Mixed photons & electrons	No	NS	Breast	42.5	17	10	5.0	0.1-49.4	NS	NS	NS	6.0
			Supine	3D IMRT	Static tangents plus electron field	Mixed photons & electrons	No	NS	Breast	42.5	17	10	5.0	0.1-49.4	NS	NS	NS	6.0
			Supine	3D IMRT	Static tangents plus electron field	Mixed photons & electrons	No	NS	Breast	42.5	17	10	5.0	0.1-49.4	NS	NS	NS	6.0
			Supine	3D IMRT	Static tangents	Low energy MV photons	No	NS	Breast	42.5	17	10	4.9	0.1-44.5	NS	NS	NS	5.7
			Supine	3D IMRT	Static tangents	Low energy MV photons	No	NS	Breast	42.5	17	10	4.9	0.1-44.5	NS	NS	NS	5.7
			Supine	3D IMRT	Static tangents	Low energy MV photons	No	NS	Breast	42.5	17	10	4.9	0.1-44.5	NS	NS	NS	5.7
Schubert	2011	USA	Supine	3D Conformal	Static tangents	Low energy MV photons	No	Yes	Breast	50	25	10	8.1	NS	0.1	NS	28.1	14.8
			Supine	3D IMRT	Static tangents	Low energy MV photons	No	No	Breast	50	25	10	6.6	NS	0.2	NS	24.5	11.8
			Supine	3D IMRT	Static tangents	Low energy MV photons	No	No	Breast	50	25	10	6.6	NS	0.2	NS	24.5	11.8
			Supine	3D IMRT	Static tangents	Low energy MV photons	No	No	Breast	50	25	10	6.6	NS	0.2	NS	24.5	11.8
			Supine	3D Conformal with FIF	Static tangents	Low energy MV photons	No	No	Breast	50	25	10	7.8	NS	0.2	NS	27.1	14.5
			Supine	3D Conformal with FIF	Static tangents	Low energy MV photons	No	No	Breast	50	25	10	7.8	NS	0.2	NS	27.1	14.5
			Supine	3D Conformal with FIF	Static tangents	Low energy MV photons	No	No	Breast	50	25	10	7.8	NS	0.2	NS	27.1	14.5
			Supine	3D IMRT	Rotational fields	Low energy MV photons	No	No	Breast	50	25	10	11.7	NS	0.6	NS	54.8	19.6
			Supine	3D IMRT	Rotational fields	Low energy MV photons	No	No	Breast	50	25	10	11.7	NS	0.6	NS	54.8	19.6
			Supine	3D IMRT	Rotational fields	Low energy MV photons	No	No	Breast	50	25	10	11.7	NS	0.6	NS	54.8	19.6
			Supine	3D IMRT	Static tangents	Low energy MV photons	No	No	Breast	50	25	10	6.6	NS	0.3	NS	20.8	12.0
			Supine	3D IMRT	Static tangents	Low energy MV photons	No	No	Breast	50	25	10	6.6	NS	0.3	NS	20.8	12.0
			Supine	3D IMRT	Static tangents	Low energy MV photons	No	No	Breast	50	25	10	6.6	NS	0.3	NS	20.8	12.0
Scorsetti	2012	Italy	Supine	3D IMRT	Rotational fields	Low energy MV photons	No	Yes	Breast	40.5	15	50	8.7	NS	2.5	NS	61.9	8.6
Semaniak	2012	Poland	Supine	3D Conformal plan with FIF	Oblique fields	Mixed energy MV photons	No	Yes	Chest wall; SCF	45	20	35	15.6	NS	NS	NS	NS	35.4
			Supine	3D Conformal plan with FIF	Oblique fields	Mixed energy MV photons	No	Yes	Chest wall; SCF	45	20	35	15.5	NS	NS	NS	NS	35.6
			Supine	3D Conformal plan with FIF	Oblique fields	Mixed energy MV photons	No	Yes	Chest wall; SCF	45	20	35	15.5	NS	NS	NS	NS	35.6
			Supine	3D Conformal plan with FIF	Oblique fields	Mixed energy MV photons	No	Yes	Chest wall; SCF	45	20	35	15.5	NS	NS	NS	NS	35.6
Shiau	2014	Taiwan	Supine	3D IMRT	Static tangents	Low energy MV photons	No	NS	Breast	50	25	30	10.0	6.2-16.4	0.9	NS	33.1	18.6
			Supine	3D IMRT	Rotational fields	Low energy MV photons	No	NS	Breast	50	25	30	6.5	4.4-9.6	0.5	NS	24.7	10.7
			Supine	3D IMRT	Rotational fields	Low energy MV photons	No	NS	Breast	50	25	30	6.5	4.4-9.6	0.5	NS	24.7	10.7
			Supine	3D IMRT	Rotational fields	Low energy MV photons	No	NS	Breast	50	25	30	6.5	4.4-9.6	0.5	NS	24.7	10.7
Sonmez	2014	Turkey	Supine	3D Conformal	Static tangents	MV photons	No	NS	Breast	NS	NS	10	8.2	NS	NS	NS	NS	14.9
			Supine	3D Conformal with FIF	Static tangents	MV photons	No	NS	Breast	NS	NS	10	7.9	NS	NS	NS	NS	13.4

Table E3 continued on the next page.

Table E3 continued from the previous page.

Author	Year	Country	Description of regimens*										Lung dose measures					
			Patient position	Treatment planning	Field type	Beam energy & modality	Breath. adapt.	RT plan delivered	Target(s)	Prescr. dose	No. of fraction	No. of CT plans	MLD_{ipsi} (Gy)		MLD_{cont} (Gy)	MLD_{whole} (Gy)	$V5_{ipsi}$ (%)	$V20_{ipsi}$ (%)
													Avg	Range				
			Supine	3D Conformal with FIF	Static tangents	MV photons	No	NS	Breast	NS	NS	10	7.9	NS	NS	NS	NS	13.4
			Supine	3D Conformal with FIF	Static tangents	MV photons	No	NS	Breast	NS	NS	10	7.9	NS	NS	NS	NS	13.4
Stillie	2011	Australia	Supine	3D Conformal with FIF	Static tangents	Low energy MV photons	No	No	Breast	50	25	10	6.0	NS	NS	NS	NS	NS
			Supine	3D IMRT	Oblique fields	Low energy MV photons	No	No	Breast	50	25	10	6.4	NS	NS	NS	NS	NS
			Supine	3D IMRT	Oblique fields	Low energy MV photons	No	No	Breast	50	25	10	6.4	NS	NS	NS	NS	NS
			Supine	3D IMRT	Oblique fields	Low energy MV photons	No	No	Breast	50	25	10	6.4	NS	NS	NS	NS	NS
			Supine	3D IMRT	Oblique fields	Low energy MV photons	No	No	Breast	50	25	10	7.1	NS	NS	NS	NS	NS
			Supine	3D IMRT	Oblique fields	Low energy MV photons	No	No	Breast	50	25	10	7.1	NS	NS	NS	NS	NS
			Supine	3D IMRT	Oblique fields	Low energy MV photons	No	No	Breast	50	25	10	7.1	NS	NS	NS	NS	NS
Subramaniam	2012	India	Supine	3D Conformal with FIF	Static tangents	Low energy MV photons	No	Yes	Chest wall; SCF	50	25	10	13.4	9.9-16.1	0.3	NS	NS	26.7
			Supine	3D IMRT	Rotational fields	Low energy MV photons	No	No	Chest wall; SCF	50	25	10	10.9	8.9-12.3	4.2	NS	NS	17.3
			Supine	3D IMRT	Rotational fields	Low energy MV photons	No	No	Chest wall; SCF	50	25	10	10.9	8.9-12.3	4.2	NS	NS	17.3
			Supine	3D IMRT	Rotational fields	Low energy MV photons	No	No	Chest wall; SCF	50	25	10	10.9	8.9-12.3	4.2	NS	NS	17.3
			Supine	3D IMRT	Rotational fields	Low energy MV photons	No	No	Chest wall; SCF	50	25	10	10.5	8.9-12.1	3.2	NS	NS	16.5
			Supine	3D IMRT	Rotational fields	Low energy MV photons	No	No	Chest wall; SCF	50	25	10	10.5	8.9-12.1	3.2	NS	NS	16.5
			Supine	3D IMRT	Rotational fields	Low energy MV photons	No	No	Chest wall; SCF	50	25	10	10.5	8.9-12.1	3.2	NS	NS	16.5
Sun L	2014	Taiwan	Supine	3D Conformal	Static tangents	Low energy MV photons	No	NS	Breast; +/- SCF; +/- IMC	50.4	28	89	NS	NS	NS	NS	NS	15.3
			Supine	3D Conformal with FIF	Static tangents	Low energy MV photons	No	NS	Breast; +/- SCF; +/- IMC	50.4	28	89	NS	NS	NS	NS	NS	15.2
			Supine	3D Conformal with FIF	Static tangents	Low energy MV photons	No	NS	Breast; +/- SCF; +/- IMC	50.4	28	89	NS	NS	NS	NS	NS	15.2
			Supine	3D Conformal with FIF	Static tangents	Low energy MV photons	No	NS	Breast; +/- SCF; +/- IMC	50.4	28	89	NS	NS	NS	NS	NS	15.2
Sung	2014	Korea	Supine	3D Conformal	Static tangents	Low energy MV photons	Yes	NS	Breast	50.4	28	22	NS	NS	NS	NS	NS	NS
			Supine	3D Conformal	Static tangents	Low energy MV photons	No	Most	Breast	50.4	28	22	NS	NS	NS	NS	NS	NS
			Supine	3D Conformal	Static tangents	Low energy MV photons	No	Most	Breast	50.4	28	22	NS	NS	NS	NS	NS	NS
			Supine	3D Conformal	Static tangents	Low energy MV photons	No	Most	Breast	50.4	28	22	NS	NS	NS	NS	NS	NS
Swamy	2014	India	Supine	3D Conformal with FIF	Static tangents	Low energy MV photons	Yes	NS	Chest wall; SCF	50	25	10	13.1	NS	0.4	NS	NS	23.5
			Supine	3D IMRT	Rotational fields	Low energy MV photons	No	No	Chest wall; SCF	50	25	10	13.0	NS	5.3	NS	NS	21.7
			Supine	3D IMRT	Rotational fields	Low energy MV photons	No	No	Chest wall; SCF	50	25	10	13.0	NS	5.3	NS	NS	21.7
			Supine	3D IMRT	Rotational fields	Low energy MV photons	No	No	Chest wall; SCF	50	25	10	13.0	NS	5.3	NS	NS	21.7
			Supine	3D IMRT	Rotational fields	Low energy MV photons	Yes	No	Chest wall; SCF	50	25	10	11.7	NS	4.4	NS	NS	18.6
			Supine	3D IMRT	Rotational fields	Low energy MV photons	Yes	No	Chest wall; SCF	50	25	10	11.7	NS	4.4	NS	NS	18.6
			Supine	3D IMRT	Rotational fields	Low energy MV photons	Yes	No	Chest wall; SCF	50	25	10	11.7	NS	4.4	NS	NS	18.6
Swanson	2012	USA	Supine	3D Conformal	Tangents plus boost/direct fields	MV photons	Yes	Yes	Breast or chest wall +/- SCF	45	NS	87	7.9	NS	NS	NS	30.0	14.0
			Supine	3D Conformal	Tangents plus boost/direct fields	MV photons	No	No	Breast or chest wall +/- SCF	45	NS	87	9.1	NS	NS	NS	32.0	17.0
			Supine	3D Conformal	Tangents plus boost/direct fields	MV photons	No	No	Breast or chest wall +/- SCF	45	NS	87	9.1	NS	NS	NS	32.0	17.0
			Supine	3D Conformal	Tangents plus boost/direct fields	MV photons	No	No	Breast or chest wall +/- SCF	45	NS	87	9.1	NS	NS	NS	32.0	17.0

Table E3 continued on the next page.

Table E3 continued from the previous page.

Author	Year	Country	Patient position	Treatment planning	Field type	Beam energy & modality	Breath. adapt.	RT plan delivered	Target(s)	Prescr. dose	No. of fraction	No. of CT plans	MLD_{ipsi} (Gy) Avg	MLD_{ipsi} (Gy) Range	MLD_{cont} (Gy)	MLD_{whole} (Gy)	$V5_{ipsi}$ (%)	$V20_{ipsi}$ (%)
			Description of regimens*										Lung dose measures					
Tan Wang	2011	China	Supine	3D Conformal	Static tangents	Low energy MV photons	No	No	Breast	50	25	10	15.4	NS	0.9	NS	38.7	25.8
			Supine	3D IMRT	Oblique fields	Low energy MV photons	No	No	Breast	50	25	10	12.1	NS	4.9	NS	82.1	23.4
			Supine	3D IMRT	Oblique fields	Low energy MV photons	No	No	Breast	50	25	10	12.1	NS	4.9	NS	82.1	23.4
			Supine	3D IMRT	Oblique fields	Low energy MV photons	No	No	Breast	50	25	10	12.1	NS	4.9	NS	82.1	23.4
			Supine	3D IMRT	Oblique fields	Low energy MV photons	No	No	Breast	50	25	10	13.4	NS	5.1	NS	83.0	17.5
			Supine	3D IMRT	Oblique fields	Low energy MV photons	No	No	Breast	50	25	10	13.4	NS	5.1	NS	83.0	17.5
			Supine	3D IMRT	Oblique fields	Low energy MV photons	No	No	Breast	50	25	10	13.4	NS	5.1	NS	83.0	17.5
			Supine	3D IMRT	Oblique fields	Low energy MV photons	No	No	Breast	50	25	10	13.4	NS	4.5	NS	80.9	18.1
			Supine	3D IMRT	Oblique fields	Low energy MV photons	No	No	Breast	50	25	10	13.4	NS	4.5	NS	80.9	18.1
			Supine	3D IMRT	Oblique fields	Low energy MV photons	No	No	Breast	50	25	10	13.4	NS	4.5	NS	80.9	18.1
			Supine	3D IMRT	Oblique fields	Low energy MV photons	No	No	Breast	50	25	10	13.3	NS	4.6	NS	81.8	17.6
			Supine	3D IMRT	Oblique fields	Low energy MV photons	No	No	Breast	50	25	10	13.3	NS	4.6	NS	81.8	17.6
			Supine	3D IMRT	Oblique fields	Low energy MV photons	No	No	Breast	50	25	10	13.3	NS	4.6	NS	81.8	17.6
Tan Liu	2012	China	Supine	3D IMRT	Oblique fields	Low energy MV photons	No	No	Breast	50	25	23	13.7	NS	5.0	NS	84.0	18.2
			Supine	3D IMRT	Oblique fields	Low energy MV photons	No	No	Breast	50	25	23	13.6	NS	4.8	NS	82.7	18.2
			Supine	3D IMRT	Oblique fields	Low energy MV photons	No	No	Breast	50	25	23	13.6	NS	4.8	NS	82.7	18.2
			Supine	3D IMRT	Oblique fields	Low energy MV photons	No	No	Breast	50	25	23	13.6	NS	4.8	NS	82.7	18.2
			Supine	3D IMRT	Oblique fields	Low energy MV photons	No	No	Breast	50	25	23	13.7	NS	5.5	NS	82.7	18.0
			Supine	3D IMRT	Oblique fields	Low energy MV photons	No	No	Breast	50	25	23	13.7	NS	5.5	NS	82.7	18.0
			Supine	3D IMRT	Oblique fields	Low energy MV photons	No	No	Breast	50	25	23	13.7	NS	5.5	NS	82.7	18.0
			Supine	3D IMRT	Oblique fields	Low energy MV photons	No	No	Breast	50	25	23	13.9	NS	4.9	NS	79.5	18.9
			Supine	3D IMRT	Oblique fields	Low energy MV photons	No	No	Breast	50	25	23	13.9	NS	4.9	NS	79.5	18.9
			Supine	3D IMRT	Oblique fields	Low energy MV photons	No	No	Breast	50	25	23	13.9	NS	4.9	NS	79.5	18.9
			Supine	3D IMRT	Oblique fields	Low energy MV photons	No	No	Breast	50	25	23	13.7	NS	5.1	NS	84.0	18.7
			Supine	3D IMRT	Oblique fields	Low energy MV photons	No	No	Breast	50	25	23	13.7	NS	5.1	NS	84.0	18.7
			Supine	3D IMRT	Oblique fields	Low energy MV photons	No	No	Breast	50	25	23	13.7	NS	5.1	NS	84.0	18.7
Tanaka	2015	Japan	Supine	3D Conformal with FIF	Static tangents	Low energy MV photons	No	NS	Breast	50	25	16	NS	NS	NS	NS	NS	15.0
Tang	2015	USA	Supine	3D Conformal	Static tangents	MV photons	Yes	Yes	Breast; +/- IMC	43-50	16-28	30	NS	NS	NS	NS	NS	NS
Tanguturi	2015	USA	Supine	3D Conformal	Static tangents	Mixed energy MV photons	No	NS	Breast or chest wall; +/- SCF; +/- IMC	50	25	148	9.9	3.6-19.9	NS	NS	32.5	18.7
			Supine	3D Conformal	Static tangents	Mixed energy MV photons	Yes	Most	Breast or chest wall; +/- SCF; +/- IMC	50	25	148	9.2	2.8-20.3	NS	NS	32.5	17.1
			Supine	3D Conformal	Static tangents	Mixed energy MV photons	Yes	Most	Breast or chest wall; +/- SCF; +/- IMC	50	25	148	9.2	2.8-20.3	NS	NS	32.5	17.1
			Supine	3D Conformal	Static tangents	Mixed energy MV photons	Yes	Most	Breast or chest wall; +/- SCF; +/- IMC	50	25	148	9.2	2.8-20.3	NS	NS	32.5	17.1
The	2012	Canada	Supine	3D IMRT	Static tangents plus electron field	Mixed photons & electrons	No	No	Breast	42.4	16	15	5.5	3.4-6.6	1.3	NS	NS	8.2
			Supine	3D IMRT	Wide static tangents plus electron field	Mixed energy MV photons	No	Yes	Breast	42.4	16	15	6.5	4.2-8.2	2.2	NS	NS	8.8
			Supine	3D IMRT	Wide static tangents plus electron field	Mixed energy MV photons	No	Yes	Breast	42.4	16	15	6.5	4.2-8.2	2.2	NS	NS	8.8

Table E3 continued on the next page.

Table E3 continued from the previous page.

Author	Year	Country	Description of regimens*										Lung dose measures					
			Patient position	Treatment planning	Field type	Beam energy & modality	Breath. adapt.	RT plan delivered	Target(s)	Prescr. dose	No. of fraction	No. of CT plans	MLD_{ipsi} (Gy)		MLD_{cont} (Gy)	MLD_{whole} (Gy)	$V5_{ipsi}$ (%)	$V20_{ipsi}$ (%)
													Avg	Range				
			Supine	3D IMRT	Wide static tangents plus electron field	Mixed energy MV photons	No	Yes	Breast	42.4	16	15	6.5	4.2-8.2	2.2	NS	NS	8.8
Thorsen	2014	Denmark	Supine	3D Conformal	Static tangents	Mixed energy MV photons	No	Yes	Breast or chest wall; SCF; IMC	48	24	38	NS	NS	NS	NS	NS	31.4
			Supine	3D Conformal	Static tangents	Mixed energy MV photons	No	Yes	Breast or chest wall; SCF;	48	24	30	NS	NS	NS	NS	NS	25.9
			Supine	3D Conformal	Static tangents	Mixed energy MV photons	No	Yes	Breast or chest wall; SCF;	48	24	30	NS	NS	NS	NS	NS	25.9
			Supine	3D Conformal	Static tangents	Mixed energy MV photons	No	Yes	Breast or chest wall; SCF;	48	24	30	NS	NS	NS	NS	NS	25.9
Trifiletti	2015	USA	Supine	3D Conformal	Static tangents	MV photons	Yes	NS	Breast	50	25	20	2.4	NS	NS	NS	NS	NS
			Supine	3D IMRT	Oblique fields	MV photons	Yes	NS	Breast	50	25	20	2.5	NS	NS	NS	NS	NS
			Supine	3D IMRT	Oblique fields	MV photons	Yes	NS	Breast	50	25	20	2.5	NS	NS	NS	NS	NS
			Supine	3D IMRT	Oblique fields	MV photons	Yes	NS	Breast	50	25	20	2.5	NS	NS	NS	NS	NS
Trofimova	2015	Russia	Supine	2D	Static tangents	MV photons	No	Yes	Breast;	50-60	NS	70	5.5	NS	NS	NS	NS	26.3
			Supine	3D Conformal	Static tangents	MV photons	No	Yes	Breast;	50-60	NS	70	5.5	NS	NS	NS	NS	26.3
			Supine	3D Conformal	Static tangents	MV photons	No	Yes	Breast;	50-60	NS	70	5.5	NS	NS	NS	NS	26.3
			Supine	3D Conformal	Static tangents	MV photons	No	Yes	Breast;	50-60	NS	70	5.5	NS	NS	NS	NS	26.3
Tsai	2012	Japan	Supine	3D IMRT	Rotational fields	Low energy MV photons	No	No	Breast	50.4	28	10	8.2	7.6-9.3	3.4	5.6	40.5	12.7
Tuschiya	2014	Japan	Supine	3D Conformal	Static tangents	MV photons	No	NS	Breast	50	25	25	8.4	NS	0.6	NS	NS	14.6
			Supine	3D IMRT	Static tangents	MV photons	No	No	Breast	50	25	25	7.7	NS	0.4	NS	NS	13.6
			Supine	3D IMRT	Static tangents	MV photons	No	No	Breast	50	25	25	7.7	NS	0.4	NS	NS	13.6
			Supine	3D IMRT	Static tangents	MV photons	No	No	Breast	50	25	25	7.7	NS	0.4	NS	NS	13.6
			Supine	3D IMRT	Oblique fields	MV photons	No	No	Breast	50	25	25	13.2	NS	2.5	NS	NS	14.8
			Supine	3D IMRT	Oblique fields	MV photons	No	No	Breast	50	25	25	13.2	NS	2.5	NS	NS	14.8
			Supine	3D IMRT	Oblique fields	MV photons	No	No	Breast	50	25	25	13.2	NS	2.5	NS	NS	14.8
			Supine	3D IMRT	Oblique fields	MV photons	No	No	Breast	50	25	25	13.0	NS	3.8	NS	NS	15.3
			Supine	3D IMRT	Oblique fields	MV photons	No	No	Breast	50	25	25	13.0	NS	3.8	NS	NS	15.3
			Supine	3D IMRT	Oblique fields	MV photons	No	No	Breast	50	25	25	13.0	NS	3.8	NS	NS	15.3
Uhl	2012	Germany	Supine	3D Conformal	Static tangents	MV photons	No	No	Breast or chest wall	50.4	28	10	16.1	NS	1.3	NS	42.0	34.5
			Supine	3D IMRT	Rotational fields	MV photons	No	Yes	Breast or chest wall	50.4	28	10	10.1	NS	5.4	NS	73.9	10.9
			Supine	3D IMRT	Rotational fields	MV photons	No	Yes	Breast or chest wall	50.4	28	10	10.1	NS	5.4	NS	73.9	10.9
			Supine	3D IMRT	Rotational fields	MV photons	No	Yes	Breast or chest wall	50.4	28	10	10.1	NS	5.4	NS	73.9	10.9
Vaegler	2014	Germany	Supine	3D Conformal	Static tangents	Low energy MV photons	No	Yes	Breast	50	25	1	9.9	NS	2.5	7.0	NS	NS
			Supine	3D IMRT	Oblique fields	Low energy MV photons	No	No	Breast	50	25	1	8.4	NS	1.9	5.8	NS	NS
			Supine	3D IMRT	Oblique fields	Low energy MV photons	No	No	Breast	50	25	1	8.4	NS	1.9	5.8	NS	NS
			Supine	3D IMRT	Oblique fields	Low energy MV photons	No	No	Breast	50	25	1	8.4	NS	1.9	5.8	NS	NS
			Supine	3D IMRT	Rotational fields	Low energy MV photons	No	No	Breast	50	25	1	7.0	NS	1.6	4.9	NS	NS
			Supine	3D IMRT	Rotational fields	Low energy MV photons	No	No	Breast	50	25	1	7.0	NS	1.6	4.9	NS	NS
			Supine	3D IMRT	Rotational fields	Low energy MV photons	No	No	Breast	50	25	1	7.0	NS	1.6	4.9	NS	NS

Table E3 continued on the next page.

Table E3 continued from the previous page.

Author	Year	Country	Patient position	Treatment planning	Field type	Beam energy & modality	Breath. adapt.	RT plan delivered	Target(s)	Prescr. dose	No. of fraction	No. of CT plans	Lung dose measures: MLD_{ipsi} (Gy) Avg	MLD_{ipsi} (Gy) Range	MLD_{cont} (Gy)	MLD_{whole} (Gy)	$V5_{ipsi}$ (%)	$V20_{ipsi}$ (%)
			Description of regimens*															
Van der Laan	2010	Netherlands	Supine	3D Conformal with FIF	Static tangents plus electron field	Mixed photons & electrons	No	NS	Chest wall; SCF; IMC	50	25	10	NS	NS	NS	10.3	NS	NS
			Supine	3D IMRT	Static tangents	MV photons	No	NS	Chest wall; SCF; IMC	50	25	10	NS	NS	NS	10.2	NS	NS
			Supine	3D IMRT	Static tangents	MV photons	No	NS	Chest wall; SCF; IMC	50	25	10	NS	NS	NS	10.2	NS	NS
			Supine	3D IMRT	Static tangents	MV photons	No	NS	Chest wall; SCF; IMC	50	25	10	NS	NS	NS	10.2	NS	NS
			Supine	3D IMRT	Static tangents plus electron field	Mixed photons & electrons	No	NS	Chest wall; SCF; IMC	50	25	10	NS	NS	NS	9.9	NS	NS
			Supine	3D IMRT	Static tangents plus electron field	Mixed photons & electrons	No	NS	Chest wall; SCF; IMC	50	25	10	NS	NS	NS	9.9	NS	NS
			Supine	3D IMRT	Static tangents plus electron field	Mixed photons & electrons	No	NS	Chest wall; SCF; IMC	50	25	10	NS	NS	NS	9.9	NS	NS
Van der Laan SIB	2010	Netherlands	Supine	3D Conformal with FIF	Static tangents	MV photons	No	NS	Breast	50.68	28	30	NS	NS	NS	3.7	NS	5.0
			Supine	3D IMRT	Static tangents	MV photons	No	NS	Breast	50.68	28	30	NS	NS	NS	3.4	NS	4.4
			Supine	3D IMRT	Static tangents	MV photons	No	NS	Breast	50.68	28	30	NS	NS	NS	3.4	NS	4.4
			Supine	3D IMRT	Static tangents	MV photons	No	NS	Breast	50.68	28	30	NS	NS	NS	3.4	NS	4.4
			Supine	3D IMRT	Static tangents	MV photons	No	NS	Breast	50.68	28	30	NS	NS	NS	3.3	NS	4.3
			Supine	3D IMRT	Static tangents	MV photons	No	NS	Breast	50.68	28	30	NS	NS	NS	3.3	NS	4.3
			Supine	3D IMRT	Static tangents	MV photons	No	NS	Breast	50.68	28	30	NS	NS	NS	3.3	NS	4.3
			Supine	3D IMRT	Static tangents	MV photons	No	NS	Breast	50.68	28	30	NS	NS	NS	3.2	NS	3.8
			Supine	3D IMRT	Static tangents	MV photons	No	NS	Breast	50.68	28	30	NS	NS	NS	3.2	NS	3.8
			Supine	3D IMRT	Static tangents	MV photons	No	NS	Breast	50.68	28	30	NS	NS	NS	3.2	NS	3.8
			Supine	3D IMRT	Static tangents	MV photons	No	NS	Breast	50.68	28	30	NS	NS	NS	3.0	NS	3.8
			Supine	3D IMRT	Static tangents	MV photons	No	NS	Breast	50.68	28	30	NS	NS	NS	3.0	NS	3.8
			Supine	3D IMRT	Static tangents	MV photons	No	NS	Breast	50.68	28	30	NS	NS	NS	3.0	NS	3.8
Van Parijs	2012	Belgium	Supine	3D Conformal with FIF	Static tangents	MV photons	No	Yes	Breast or chest wall; SCF; axilla	50	25	31	6.6	NS	NS	NS	NS	NS
			Supine	3D IMRT	Rotational fields	MV photons	No	Yes	Breast or chest wall; SCF; axilla	42	15	38	4.7	NS	NS	NS	NS	NS
			Supine	3D IMRT	Rotational fields	MV photons	No	Yes	Breast or chest wall; SCF; axilla	42	15	38	4.7	NS	NS	NS	NS	NS
			Supine	3D IMRT	Rotational fields	MV photons	No	Yes	Breast or chest wall; SCF; axilla	42	15	38	4.7	NS	NS	NS	NS	NS
Varga	2013	Hungary	Supine	3D Conformal with FIF	Static tangents	Mixed energy MV photons	No	NS	Breast	50	NS	83	6.3	NS	NS	NS	NS	11.9
			Prone	3D Conformal with FIF	Static tangents	Mixed energy MV photons	No	NS	Breast	50	NS	83	1.0	NS	NS	NS	NS	1.3
			Prone	3D Conformal with FIF	Static tangents	Mixed energy MV photons	No	NS	Breast	50	NS	83	1.0	NS	NS	NS	NS	1.3
			Prone	3D Conformal with FIF	Static tangents	Mixed energy MV photons	No	NS	Breast	50	NS	83	1.0	NS	NS	NS	NS	1.3
Vera	2014	USA	Supine	3D Conformal	Oblique fields	Mixed energy MV photons	No	Yes	Partial breast	38.5	10	59	NS	NS	NS	NS	NS	1.0
Verhoeven	2013	Belgium	Supine	3D Conformal with FIF	Static tangents	MV photons	No	NS	Breast	50	25	34	NS	NS	NS	4.0	NS	7.3
			Supine	3D Conformal	Static tangents	MV photons	Yes	NS	Breast	50	25	34	NS	NS	NS	3.4	NS	5.6
			Supine	3D Conformal	Static tangents	MV photons	Yes	NS	Breast	50	25	34	NS	NS	NS	3.4	NS	5.6
			Supine	3D Conformal	Static tangents	MV photons	Yes	NS	Breast	50	25	34	NS	NS	NS	3.4	NS	5.6
			Prone	3D Conformal with FIF	Static tangents	MV photons	No	NS	Breast	50	25	34	NS	NS	NS	1.7	NS	2.3

Table E3 continued on the next page.

Table E3 continued from the previous page.

Author	Year	Country	Description of regimens* Patient position	Treatment planning	Field type	Beam energy & modality	Breath. adapt.	RT plan delivered	Target(s)	Prescr. dose	No. of fraction	No. of CT plans	Lung dose measures MLD_{ipsi} (Gy) Avg	Range	MLD_{cont} (Gy)	MLD_{whole} (Gy)	$V5_{ipsi}$ (%)	$V20_{ipsi}$ (%)
			Prone	3D Conformal with FIF	Static tangents	MV photons	No	NS	Breast	50	25	34	NS	NS	NS	1.7	NS	2.3
			Prone	3D Conformal with FIF	Static tangents	MV photons	No	NS	Breast	50	25	34	NS	NS	NS	1.7	NS	2.3
Vikstrom	2011	Norway	Supine	3D Conformal with FIF	Static tangents	Mixed energy MV photons	No	NS	Breast	50	25	17	6.9	4.8-10.2	NS	NS	NS	12.2
			Supine	3D Conformal with FIF	Static tangents	Mixed energy MV photons	Yes	NS	Breast	50	25	17	5.9	4.0-7.9	NS	NS	NS	10.0
			Supine	3D Conformal with FIF	Static tangents	Mixed energy MV photons	Yes	NS	Breast	50	25	17	5.9	4.0-7.9	NS	NS	NS	10.0
			Supine	3D Conformal with FIF	Static tangents	Mixed energy MV photons	Yes	NS	Breast	50	25	17	5.9	4.0-7.9	NS	NS	NS	10.0
Viren	2015	Finland	Supine	3D Conformal with FIF	Static tangents	MV photons	No	NS	Breast	50	25	10	10.4	NS	0.7	NS	32.0	21.4
			Supine	3D IMRT	Static tangents	MV photons	No	No	Breast	50	25	10	10.9	NS	0.7	NS	33.3	21.6
			Supine	3D IMRT	Static tangents	MV photons	No	No	Breast	50	25	10	10.9	NS	0.7	NS	33.3	21.6
			Supine	3D IMRT	Static tangents	MV photons	No	No	Breast	50	25	10	10.9	NS	0.7	NS	33.3	21.6
			Supine	3D IMRT	Rotational fields	MV photons	No	No	Breast	50	25	10	8.7	NS	1.6	NS	35.5	15.3
			Supine	3D IMRT	Rotational fields	MV photons	No	No	Breast	50	25	10	8.7	NS	1.6	NS	35.5	15.3
			Supine	3D IMRT	Rotational fields	MV photons	No	No	Breast	50	25	10	8.7	NS	1.6	NS	35.5	15.3
			Supine	3D IMRT	Rotational fields	MV photons	No	No	Breast	50	25	10	9.6	NS	0.9	NS	36.9	18.1
			Supine	3D IMRT	Rotational fields	MV photons	No	No	Breast	50	25	10	9.6	NS	0.9	NS	36.9	18.1
			Supine	3D IMRT	Rotational fields	MV photons	No	No	Breast	50	25	10	9.6	NS	0.9	NS	36.9	18.1
Vrana	2013	Czech	Supine	3D Conformal	Static tangents	MV photons	No	No	NS	50	25	1	15.9	NS	NS	NS	NS	NS
			Supine	3D Conformal	Static tangents	MV photons	Yes	Yes	NS	50	25	1	13.6	NS	NS	NS	NS	NS
			Supine	3D Conformal	Static tangents	MV photons	Yes	Yes	NS	50	25	1	13.6	NS	NS	NS	NS	NS
			Supine	3D Conformal	Static tangents	MV photons	Yes	Yes	NS	50	25	1	13.6	NS	NS	NS	NS	NS
Wang W	2012	Canada	Supine	3D Conformal with FIF	Static tangents	MV photons	No	No	Breast	42.4/50	16/25	20	5.2	3.2-9.3	NS	NS	NS	NS
			Supine	3D Conformal with FIF	Static tangents	MV photons	Yes	Yes	Breast	42.4/50	16/25	20	5.0	2.9-9.2	NS	NS	NS	NS
			Supine	3D Conformal with FIF	Static tangents	MV photons	Yes	Yes	Breast	42.4/50	16/25	20	5.0	2.9-9.2	NS	NS	NS	NS
			Supine	3D Conformal with FIF	Static tangents	MV photons	Yes	Yes	Breast	42.4/50	16/25	20	5.0	2.9-9.2	NS	NS	NS	NS
Wang RadRes	2013	China	Supine	3D Conformal with FIF	Static tangents	MV photons	No	NS	Breast	50	25	17	10.1	NS	NS	NS	31.4	18.0
Wang J	2015	China	Supine	3D Conformal	Static tangents	Low energy MV photons	No	NS	Chest wall; SCF; IMC	50	25	30	11.7	NS	NS	NS	45.3	28.6
			Supine	3D IMRT	Static tangents	Low energy MV photons	No	No	Chest wall; SCF; IMC	50	25	30	9.1	NS	NS	NS	41.2	26.3
			Supine	3D IMRT	Static tangents	Low energy MV photons	No	No	Chest wall; SCF; IMC	50	25	30	9.1	NS	NS	NS	41.2	26.3
			Supine	3D IMRT	Static tangents	Low energy MV photons	No	No	Chest wall; SCF; IMC	50	25	30	9.1	NS	NS	NS	41.2	26.3
			Supine	3D IMRT	Oblique fields	Low energy MV photons	No	No	Chest wall; SCF; IMC	50	25	30	9.3	NS	NS	NS	44.3	24.1
			Supine	3D IMRT	Oblique fields	Low energy MV photons	No	No	Chest wall; SCF; IMC	50	25	30	9.3	NS	NS	NS	44.3	24.1
			Supine	3D IMRT	Oblique fields	Low energy MV photons	No	No	Chest wall; SCF; IMC	50	25	30	9.3	NS	NS	NS	44.3	24.1
			Supine	3D IMRT	Rotational fields	Low energy MV photons	No	No	Chest wall; SCF; IMC	50	25	30	10.8	NS	NS	NS	48.9	22.6
			Supine	3D IMRT	Rotational fields	Low energy MV photons	No	No	Chest wall; SCF; IMC	50	25	30	10.8	NS	NS	NS	48.9	22.6
			Supine	3D IMRT	Rotational fields	Low energy MV photons	No	No	Chest wall; SCF; IMC	50	25	30	10.8	NS	NS	NS	48.9	22.6
Wang X	2013	USA	Supine	3D Conformal	Static tangents	MV photons	No	Yes	Partial breast	38.5	10	11	3.1	0.4-6.1	0.1	NS	18.4	3.5
			Supine	3D IMRT	Static tangents plus electron field	Protons	No	No	Partial breast	38.5	10	11	0.8	0.2-1.44	0.0	NS	4.9	0.2

Table E3 continued on the next page.

Table E3 continued from the previous page.

Author	Year	Country	Description of regimens* Patient position	Treatment planning	Field type	Beam energy & modality	Breath. adapt.	RT plan delivered	Target(s)	Prescr. dose	No. of fraction	No. of CT plans	Lung dose measures MLD$_{ipsi}$ (Gy) Avg	MLD$_{ipsi}$ (Gy) Range	MLD$_{cont}$ (Gy)	MLD$_{whole}$ (Gy)	V5$_{ipsi}$ (%)	V20$_{ipsi}$ (%)
			Supine	3D IMRT	Static tangents plus electron field	Protons	No	No	Partial breast	38.5	10	11	0.8	0.2-1.44	0.0	NS	4.9	0.2
			Supine	3D IMRT	Static tangents plus electron field	Protons	No	No	Partial breast	38.5	10	11	0.8	0.2-1.44	0.0	NS	4.9	0.2
			Supine	3D IMRT	Oblique fields	Protons	No	No	Partial breast	38.5	10	11	0.6	0.08-1.28	0.0	NS	3.5	0.1
			Supine	3D IMRT	Oblique fields	Protons	No	No	Partial breast	38.5	10	11	0.6	0.08-1.28	0.0	NS	3.5	0.1
			Supine	3D IMRT	Oblique fields	Protons	No	No	Partial breast	38.5	10	11	0.6	0.08-1.28	0.0	NS	3.5	0.1
			Supine	3D Conformal	Oblique fields	Protons	No	No	Partial breast	38.5	10	11	0.8	0-1.9	0.0	NS	5.3	1.4
			Supine	3D Conformal	Oblique fields	Protons	No	No	Partial breast	38.5	10	11	0.8	0-1.9	0.0	NS	5.3	1.4
			Supine	3D Conformal	Oblique fields	Protons	No	No	Partial breast	38.5	10	11	0.8	0-1.9	0.0	NS	5.3	1.4
Wright	2014	Finland	Supine	3D Conformal	Static tangents	MV photons	No	NS	Chest wall; SCF; IMC; axilla	50	25	10	16.4	15.3-17.6	0.4	NS	NS	32.2
			Supine	3D Conformal	Static tangents plus electron field	Mixed photons & electrons	No	NS	Chest wall; SCF; IMC; axilla	50	25	10	16.3	15-17.6	0.6	NS	NS	30.9
			Supine	3D Conformal	Static tangents plus electron field	Mixed photons & electrons	No	NS	Chest wall; SCF; IMC; axilla	50	25	10	16.3	15-17.6	0.6	NS	NS	30.9
			Supine	3D Conformal	Static tangents plus electron field	Mixed photons & electrons	No	NS	Chest wall; SCF; IMC; axilla	50	25	10	16.3	15-17.6	0.6	NS	NS	30.9
			Supine	3D IMRT	Static tangents plus electron field	Mixed photons & electrons	No	NS	Chest wall; SCF; IMC; axilla	50	25	10	15.4	12.4-16.9	0.4	NS	NS	30.6
			Supine	3D IMRT	Static tangents plus electron field	Mixed photons & electrons	No	NS	Chest wall; SCF; IMC; axilla	50	25	10	15.4	12.4-16.9	0.4	NS	NS	30.6
			Supine	3D IMRT	Static tangents plus electron field	Mixed photons & electrons	No	NS	Chest wall; SCF; IMC; axilla	50	25	10	15.4	12.4-16.9	0.4	NS	NS	30.6
Wu He	2014	China	Supine	3D Conformal plan with FIF	Static tangents	Low energy MV photons	No	No	Breast	50	25	29	7.4	1.4-10.2	NS	NS	25.1	13.2
			Supine	3D IMRT	Static tangents	Low energy MV photons	No	Yes	Partial breast	38.5	10	29	3.2	2.2-4.3	NS	NS	19.5	2.0
			Supine	3D IMRT	Static tangents	Low energy MV photons	No	Yes	Partial breast	38.5	10	29	3.2	2.2-4.3	NS	NS	19.5	2.0
			Supine	3D IMRT	Static tangents	Low energy MV photons	No	Yes	Partial breast	38.5	10	29	3.2	2.2-4.3	NS	NS	19.5	2.0
Wu Lai	2015	China	Supine	3D IMRT	Static tangents	MV photons	No	NS	Breast	50.4	28	10	5.0	NS	NS	NS	18.1	9.2
			Supine	3D IMRT	Tangents plus boost/direct fields	Mixed photons & electrons	No	NS	Breast	50.4	28	10	5.3	NS	NS	NS	19.3	9.3
			Supine	3D IMRT	Tangents plus boost/direct fields	Mixed photons & electrons	No	NS	Breast	50.4	28	10	5.3	NS	NS	NS	19.3	9.3
			Supine	3D IMRT	Tangents plus boost/direct fields	Mixed photons & electrons	No	NS	Breast	50.4	28	10	5.3	NS	NS	NS	19.3	9.3
			Supine	3D IMRT	Rotational fields	MV photons	No	NS	Breast	50.4	28	10	7.6	NS	NS	NS	39.8	12.1
			Supine	3D IMRT	Rotational fields	MV photons	No	NS	Breast	50.4	28	10	7.6	NS	NS	NS	39.8	12.1
			Supine	3D IMRT	Rotational fields	MV photons	No	NS	Breast	50.4	28	10	7.6	NS	NS	NS	39.8	12.1
Wurschmidt	2014	Germany	Supine	3D Conformal plan with FIF	Static tangents	Low energy MV phtons	No	No	Breast	50.4	28	46	NS	NS	NS	8.4	NS	NS
			Prone	3D Conformal plan with FIF	Static tangents	Low energy MV phtons	No	No	Breast	50.4	28	46	NS	NS	NS	2.3	NS	NS
			Prone	3D Conformal plan with FIF	Static tangents	Low energy MV phtons	No	No	Breast	50.4	28	46	NS	NS	NS	2.3	NS	NS
			Prone	3D Conformal plan with FIF	Static tangents	Low energy MV phtons	No	No	Breast	50.4	28	46	NS	NS	NS	2.3	NS	NS
Xie	2014	China	Supine	3D Conformal	Static tangents	Low energy MV photons	No	NS	Breast	50	25	8	10.0	NS	0.9	NS	28.8	17.3
			Supine	3D IMRT	Static tangents	Low energy MV photons	No	NS	Breast	50	25	8	11.3	NS	1.4	NS	44.9	17.8
			Supine	3D IMRT	Static tangents	Low energy MV photons	No	NS	Breast	50	25	8	11.3	NS	1.4	NS	44.9	17.8

Table E3 continued on the next page.

Table E3 continued from the previous page.

Author	Year	Country	Description of regimens* Patient position	Treatment planning	Field type	Beam energy & modality	Breath. adapt.	RT plan delivered	Target(s)	Prescr. dose	No. of fraction	No. of CT plans	Lung dose measures MLD_{ipsi} (Gy) Avg	MLD_{ipsi} (Gy) Range	MLD_{cont} (Gy)	MLD_{whole} (Gy)	$V5_{ipsi}$ (%)	$V20_{ipsi}$ (%)
			Supine	3D IMRT	Static tangents	Low energy MV photons	No	NS	Breast	50	25	8	11.3	NS	1.4	NS	44.9	17.8
			Supine	3D IMRT	Oblique fields	Low energy MV photons	No	NS	Breast	50	25	8	11.1	NS	1.7	NS	44.4	17.7
			Supine	3D IMRT	Oblique fields	Low energy MV photons	No	NS	Breast	50	25	8	11.1	NS	1.7	NS	44.4	17.7
			Supine	3D IMRT	Oblique fields	Low energy MV photons	No	NS	Breast	50	25	8	11.1	NS	1.7	NS	44.4	17.7
Xu	2013	USA	Supine	3D Conformal	Static tangents plus electron field	Mixed photons & electrons	No	No	Breast or chest wall; SCF; axilla; superior IMC	50.4	28	10	10.0	0.7-23	0.6	NS	70.0	36.0
			Supine	3D Conformal	Tangents plus boost/direct fields	Protons plus photons	No	No	Breast or chest wall; SCF; axilla; superior IMC	50.4	28	10	5.5	2.0-18	0.4	NS	50.0	31.0
			Supine	3D Conformal	Tangents plus boost/direct fields	Protons plus photons	No	No	Breast or chest wall; SCF; axilla; superior IMC	50.4	28	10	5.5	2.0-18	0.4	NS	50.0	31.0
			Supine	3D Conformal	Tangents plus boost/direct fields	Protons plus photons	No	No	Breast or chest wall; SCF; axilla; superior IMC	50.4	28	10	5.5	2.0-18	0.4	NS	50.0	31.0
			Supine	3D IMRT	Tangents plus boost/direct fields	MV photons	No	No	Breast or chest wall; SCF; axilla; superior IMC	50.4	28	10	11.0	10.0-15	2.0	NS	81.0	30.0
			Supine	3D IMRT	Tangents plus boost/direct fields	MV photons	No	No	Breast or chest wall; SCF; axilla; superior IMC	50.4	28	10	11.0	10.0-15	2.0	NS	81.0	30.0
			Supine	3D IMRT	Tangents plus boost/direct fields	MV photons	No	No	Breast or chest wall; SCF; axilla; superior IMC	50.4	28	10	11.0	10.0-15	2.0	NS	81.0	30.0
Yang B	2014	USA	Supine	3D Conformal	Static tangents plus electron field	Low energy MV photons	No	NS	Chest wall; SCF; +/- IMC	50	25	246	10.9	NS	5.3	NS	NS	19.3
			Supine	3D IMRT	Oblique fields	Low energy MV photons	No	NS	Chest wall; SCF; +/- IMC	50	25	246	10.9	NS	4.7	NS	NS	19.4
			Supine	3D IMRT	Oblique fields	Low energy MV photons	No	NS	Chest wall; SCF; +/- IMC	50	25	246	10.9	NS	4.7	NS	NS	19.4
			Supine	3D IMRT	Oblique fields	Low energy MV photons	No	NS	Chest wall; SCF; +/- IMC	50	25	246	10.9	NS	4.7	NS	NS	19.4
Yang D	2014	Korea	Supine	3D Conformal	Static tangents	Low energy MV photons	No	NS	Breast	50.4	28	16	NS	NS	NS	NS	19.6	14.4
			Supine	3D Conformal	Static tangents	Low energy MV photons	No	NS	Breast	50.4	28	16	NS	NS	NS	NS	20.2	14.5
			Supine	3D Conformal	Static tangents	Low energy MV photons	No	NS	Breast	50.4	28	16	NS	NS	NS	NS	20.2	14.5
			Supine	3D Conformal	Static tangents	Low energy MV photons	No	NS	Breast	50.4	28	16	NS	NS	NS	NS	20.2	14.5
			Supine	3D Conformal with FIF	Static tangents	Low energy MV photons	No	NS	Breast	50.4	28	16	NS	NS	NS	NS	19.7	14.4
			Supine	3D Conformal with FIF	Static tangents	Low energy MV photons	No	NS	Breast	50.4	28	16	NS	NS	NS	NS	19.7	14.4
			Supine	3D Conformal with FIF	Static tangents	Low energy MV photons	No	NS	Breast	50.4	28	16	NS	NS	NS	NS	19.7	14.4
			Supine	3D Conformal with FIF	Static tangents	Low energy MV photons	No	NS	Breast	50.4	28	16	NS	NS	NS	NS	19.6	14.3
			Supine	3D Conformal with FIF	Static tangents	Low energy MV photons	No	NS	Breast	50.4	28	16	NS	NS	NS	NS	19.6	14.3
			Supine	3D Conformal with FIF	Static tangents	Low energy MV photons	No	NS	Breast	50.4	28	16	NS	NS	NS	NS	19.6	14.3
Yang W	2015	USA	Supine	3D Conformal with FIF	Static tangents	Mixed energy MV photons	No	No	Breast or chest wall; +/- SCF	42.6-50.4	16-28	28	NS	NS	NS	NS	NS	9.5
			Supine	3D Conformal with FIF	Static tangents	Mixed energy MV photons	Yes	Yes	Breast or chest wall; +/- SCF	42.6-50.4	16-28	28	NS	NS	NS	NS	NS	8.0

Table E3 continued on the next page.

Table E3 continued from the previous page.

Author	Year	Country	Description of regimens* Patient position	Treatment planning	Field type	Beam energy & modality	Breath. adapt.	RT plan delivered	Target(s)	Prescr. dose	No. of fraction	No. of CT plans	Lung dose measures MLD$_{ipsi}$ (Gy) Avg	MLD$_{ipsi}$ (Gy) Range	MLD$_{cont}$ (Gy)	MLD$_{whole}$ (Gy)	V5$_{ipsi}$ (%)	V20$_{ipsi}$ (%)
			Supine	3D Conformal with FIF	Static tangents	Mixed energy MV photons	Yes	Yes	Breast or chest wall; +/- SCF	42.6-50.4	16-28	28	NS	NS	NS	NS	NS	8.0
			Supine	3D Conformal with FIF	Static tangents	Mixed energy MV photons	Yes	Yes	Breast or chest wall; +/- SCF	42.6-50.4	16-28	28	NS	NS	NS	NS	NS	8.0
Yavas	2012	Turkey	Supine	3D Conformal	Static tangents	MV photons	No	NS	Breast	50	25	20	9.8	NS	NS	NS	NS	NS
			Supine	3D Conformal with FIF	Static tangents	MV photons	No	NS	Breast	50	25	20	7.7	NS	NS	NS	NS	NS
			Supine	3D Conformal with FIF	Static tangents	MV photons	No	NS	Breast	50	25	20	7.7	NS	NS	NS	NS	NS
			Supine	3D Conformal with FIF	Static tangents	MV photons	No	NS	Breast	50	25	20	7.7	NS	NS	NS	NS	NS
Yim	2015	Australia	Supine	3D Conformal	Static tangents	Mixed energy MV photons	No	Yes	Breast	50	25	25	6.3	0.1-8.2	0.0	NS	NS	NS
			Supine	3D IMRT	Static tangents	Mixed energy MV photons	No	No	Breast	50	25	25	5.9	0.1-8.6	0.0	NS	NS	NS
			Supine	3D IMRT	Static tangents	Mixed energy MV photons	No	No	Breast	50	25	25	5.9	0.1-8.6	0.0	NS	NS	NS
			Supine	3D IMRT	Static tangents	Mixed energy MV photons	No	No	Breast	50	25	25	5.9	0.1-8.6	0.0	NS	NS	NS
Yin	2012	China	Supine	3D Conformal with FIF	Static tangents	Low energy MV photons	No	NS	Breast	50	25	10	12.1	NS	0.2	NS	36.6	22.5
			Supine	3D IMRT	Oblique fields	Low energy MV photons	No	NS	Breast	50	25	10	13.6	NS	5.5	NS	86.1	19.6
			Supine	3D IMRT	Oblique fields	Low energy MV photons	No	NS	Breast	50	25	10	13.6	NS	5.5	NS	86.1	19.6
			Supine	3D IMRT	Oblique fields	Low energy MV photons	No	NS	Breast	50	25	10	13.6	NS	5.5	NS	86.1	19.6
			Supine	3D IMRT	Rotational fields	Low energy MV photons	No	NS	Breast	50	25	10	1.2	NS	5.8	NS	61.8	18.9
			Supine	3D IMRT	Rotational fields	Low energy MV photons	No	NS	Breast	50	25	10	1.2	NS	5.8	NS	61.8	18.9
			Supine	3D IMRT	Rotational fields	Low energy MV photons	No	NS	Breast	50	25	10	1.2	NS	5.8	NS	61.8	18.9
Zhang	2010	China	Supine	3D Conformal	Static tangents plus electron field	Mixed photons & electrons	No	NS	Chest wall; IMC	50	25	30	13.2	NS	NS	NS	69.7	18.8
			Supine	3D Conformal	Wide static tangents	Low energy MV photons	No	NS	Chest wall; IMC	50	25	30	13.4	NS	NS	NS	36.2	26.5
			Supine	3D Conformal	Wide static tangents	Low energy MV photons	No	NS	Chest wall; IMC	50	25	30	13.4	NS	NS	NS	36.2	26.5
			Supine	3D Conformal	Wide static tangents	Low energy MV photons	No	NS	Chest wall; IMC	50	25	30	13.4	NS	NS	NS	36.2	26.5
			Supine	3D Conformal	Wide static tangents	Low energy MV photons	No	NS	Chest wall; IMC	50	25	30	12.2	NS	NS	NS	34.7	24.8
			Supine	3D Conformal	Wide static tangents	Low energy MV photons	No	NS	Chest wall; IMC	50	25	30	12.2	NS	NS	NS	34.7	24.8
			Supine	3D Conformal	Wide static tangents	Low energy MV photons	No	NS	Chest wall; IMC	50	25	30	12.2	NS	NS	NS	34.7	24.8
			Supine	3D IMRT	Wide static tangents	Low energy MV photons	No	NS	Chest wall; IMC	50	25	30	15.6	NS	NS	NS	81.4	23.1
			Supine	3D IMRT	Wide static tangents	Low energy MV photons	No	NS	Chest wall; IMC	50	25	30	15.6	NS	NS	NS	81.4	23.1
			Supine	3D IMRT	Wide static tangents	Low energy MV photons	No	NS	Chest wall; IMC	50	25	30	15.6	NS	NS	NS	81.4	23.1
Zhang L	2015	China	Supine	3D Conformal with FIF	Static tangents	MV photons	No	NS	Breast; axilla	50	25	61	8.9	8.5-9.2	NS	NS	NS	16.8
			Supine	3D IMRT	Oblique fields	MV photons	No	NS	Breast; axilla	50	25	61	8.5	8.2-8.8	NS	NS	NS	14.7
			Supine	3D IMRT	Oblique fields	MV photons	No	NS	Breast; axilla	50	25	61	8.5	8.2-8.8	NS	NS	NS	14.7
			Supine	3D IMRT	Oblique fields	MV photons	No	NS	Breast; axilla	50	25	61	8.5	8.2-8.8	NS	NS	NS	14.7

Table E3 continued on the next page.

Table E3 continued from the previous page.

Author	Year	Country	Description of regimens* Patient position	Treatment planning	Field type	Beam energy & modality	Breath. adapt.	RT plan delivered	Target(s)	Prescr. dose	No. of fraction	No. of CT plans	Lung dose measures MLD$_{ipsi}$ (Gy) Avg	Range	MLD$_{cont}$ (Gy)	MLD$_{whole}$ (Gy)	V5$_{ipsi}$ (%)	V20$_{ipsi}$ (%)
Zhang Q	2015	China	Supine	3D IMRT	Oblique fields	MV photons	No	NS	Chest wall; IMC	50	25	15	14.1	NS	4.7	NS	66.0	24.2
			Supine	3D IMRT	Rotational fields	MV photons	No	NS	Chest wall; IMC	50	25	15	12.8	NS	4.5	NS	61.1	21.0
			Supine	3D IMRT	Rotational fields	MV photons	No	NS	Chest wall; IMC	50	25	15	12.8	NS	4.5	NS	61.1	21.0
			Supine	3D IMRT	Rotational fields	MV photons	No	NS	Chest wall; IMC	50	25	15	12.8	NS	4.5	NS	61.1	21.0
Zhou	2011	China	Supine	3D Conformal	Static tangents	Low energy MV photons	No	Yes	Breast or chest wall	50	25	10	15.1	NS	6.2	NS	85.0	20.0
			Supine	3D IMRT	Static tangents	Low energy MV photons	No	No	Breast or chest wall	50	25	10	13.7	NS	6.4	NS	84.0	20.0
			Supine	3D IMRT	Static tangents	Low energy MV photons	No	No	Breast or chest wall	50	25	10	13.7	NS	6.4	NS	84.0	20.0
			Supine	3D IMRT	Static tangents	Low energy MV photons	No	No	Breast or chest wall	50	25	10	13.7	NS	6.4	NS	84.0	20.0
			Supine	3D IMRT	Rotational fields	Low energy MV photons	No	No	Breast or chest wall	50	25	10	17.6	NS	0.9	NS	45.0	34.0
			Supine	3D IMRT	Rotational fields	Low energy MV photons	No	No	Breast or chest wall	50	25	10	17.6	NS	0.9	NS	45.0	34.0
			Supine	3D IMRT	Rotational fields	Low energy MV photons	No	No	Breast or chest wall	50	25	10	17.6	NS	0.9	NS	45.0	34.0

* Definitions of radiotherapy categories are in Tables E1 and E2. Tumor bed boost descriptions are omitted.

† Whole heart dose averaged over the CT plans contributing to each regimen.

‡ Standard deviation and range of whole heart doses contributing to each regimen.

V30$_{ipsi}$ and V30$_{ipsi}$ are omitted as the were only reported in 43 and 13 studies respectively.

Abbreviations

2D = 2-dimensional
FIF = field-in-field
IMRT = intensity modulated radiation therapy
NA = not appliable
RT = radiotherapy
SD = standard deviation
3D = 3-dimensional
IMC = internal mammary chain
MV = megavoltage
NS = not specified
SCF = supraclavicular fossa
MLD$_{ipsi}$ = Mean ipsilateral lung doses
MLD$_{cont}$ = Mean contralateral lung doses
MLD$_{whole}$ = Mean whole lung doses
V5$_{ipsi}$ = Percent volume of ipsilateral lung receiving 5 Gy or more
V20$_{ipsi}$ = Percent volume of ipsilateral lung receiving 20 Gy or more

Table E4: 198 eligible publications reporting lung dose from breast cancer radiotherapy and included in this study.

Aaron J, Sasidharan B, Ebenezer S et al. Impact of breathing on post-mastectomy radiotherapy: A dosimetric comparison between intensity-modulated radiotherapy and 3D tangential radiotherapy. Journal of Radiotherapy in Practice. 2014;14:126-34.

Abo-Madyan Y, Aziz MH, Aly M, et al. Second cancer risk after 3D-CRT, IMRT and VMAT for breast cancer. Radiother Oncol. 2014;110:471-6.

Adam D, Suditu MD, Popa R, Ciocaltei V. Volumetric-modulated arc therapy vs. 3D-conformal radiotherapy for breast cancer. Romanian Reports in Physics. 2015;67:978-86.

Akamatsu H, Karasawa K, Omatsu T et al. First experience of carbon-ion radiotherapy for early breast cancer. Japanese Journal of Radiology. 2014;32:288-95.

Alco G, Igdem SI, Ercan T et al. Coverage of axillary lymph nodes with high tangential fields in breast radiotherapy. Brit J Radiol. 2010;83:1072-6.

Al-Rahbi ZS, Al Mandhari Z, Ravichandran R et al. Dosimetric comparison of intensity modulated radiotherapy isocentric field plans and field in field (FIF) forward plans in the treatment of breast cancer. Journal of medical physics / Association of Medical Physicists of India. 2013;38:22-9.

Aly M, Glatting G, Jahnke L, Wenz F, Abo-Madyan Y. Comparison of breast simultaneous integrated boost (SIB) radiotherapy techniques. Radiat Oncol. 2015;10:139.

Amoush A, Murray E, Yu JS, Xia P. Single-isocenter hybrid IMRT plans versus two-isocenter conventional plans and impact of intrafraction motion for the treatment of breast cancer with supraclavicular lymph nodes involvement. J Appl Clin Med Phys. 2015;16:31-9.

Anbumani S, Palled SR, Prabhakar GS, Nambiraj NA, Pichandi A. Accelerated partial breast irradiation using external beam radiotherapy-A feasibility study based on dosimetric analysis. Reports of Practical Oncology and Radiotherapy. 2012;17:200-6.

Arenas M, Hernandez V, Farrus B et al. Do breast cups improve breast cancer dosimetry? A comparative study for patients with large or pendulous breasts. Acta Oncologica. 2014;53:795-801.

Ares C, Khan S, Macartain AM et al. Postoperative Proton Radiotherapy for Localized and Locoregional Breast Cancer: Potential for Clinically Relevant Improvements? Int J Radiat Oncol Biol Phys. 2010;76:685-97.

Arora D, Frakes J, Scott J et al. Incidental radiation to uninvolved internal mammary lymph nodes in breast cancer. Breast Cancer Research and Treatment. 2015;151:365-72.

Badakhshi H, Kaul D, Nadobny J et al. Image-guided volumetric modulated arc therapy for breast cancer: a feasibility study and plan comparison with three-dimensional conformal and intensity-modulated radiotherapy. Br J Radiol. 2013;86:20130515.

Banaei A, Hashemi B, Bakhshandeh M. Comparing the monoisocentric and dual isocentric techniques in chest wall radiotherapy of mastectomy patients. J Appl Clin Med Phys. 2015;16:130-8.

Barsoum M, Mostafa M, El Hossieny H et al. Dosimetric prospective study comparing 2D and 3D planning for irradiation of supraclavicular and infraclavicular regions in breast cancer patients. Journal of the Egyptian National Cancer Institute. 2015;27:25-34.

Bartlett FR, Colgan RM, Donovan EM et al. The UK HeartSpare Study (Stage IB): Randomised comparison of a voluntary breath-hold technique and prone radiotherapy after breast conserving surgery. Radiother Oncol. 2014;114:66-72.

Belkacemi Y, Bigorie V, Pan Q, et al. Breast Radiotherapy (RT) Using Tangential Fields (TgF): A Prospective Evaluation of the Dose Distribution in the Sentinel Lymph Node (SLN) Area as Determined Intraoperatively by Clip Placement. Ann Surg Oncol. 2014;21:3758-65.

Blom Goldman U, Anderson M, Wennberg B, Lind P. Radiation pneumonitis and pulmonary function with lung dose-volume constraints in breast cancer irradiation. Journal of Radiotherapy in Practice. 2014;13:211-7.

Blom Goldman U, Svane G, Anderson M, Wennberg B, Lind P. Long-term functional and radiological pulmonary changes after radiation therapy for breast cancer. Acta Oncologica. 2014;53:1373-9.

Bodacs I, Polgar C, Major T. [Dosimetric comparison of external partial breast irradiation with whole breast irradiation and partial breast brachytherapy]. [Hungarian]. Magyar onkologia. 2014;58:108-15.

Bolukbasi Y, Saglam Y, Selek U, et al. Reproducible deep-inspiration breath-hold irradiation with forward intensity-modulated radiotherapy for left-sided breast cancer significantly reduces cardiac radiation exposure compared to inverse intensity-modulated radiotherapy. Tumori. 2014;100:169-78.

Borca VC, Franco P, Catuzzo P, et al. Does TomoDirect 3DCRT represent a suitable option for post-operative whole breast irradiation? A hypothesis-generating pilot study. Radiat Oncol. 2012;7:211.

Borges C, Cunha G, Monteiro-Grillo I, Vaz P, Teixeira N. Comparison of different breast planning techniques and algorithms for radiation therapy treatment. Physica Medica. 2014;30:160-70.

Bourgier C, Pichenot C, Verstraet R, et al. Early Side Effects of Three-Dimensional Conformal External Beam Accelerated Partial Breast Irradiation to a Total Dose of 40 Gy in One Week (a Phase II Trial). Int J Radiat Oncol Biol Phys. 2011;81:1228-35.

Bruzzaniti V, Abate A, Pinnaro P, et al. Dosimetric and clinical advantages of deep inspiration breath-hold (DIBH) during radiotherapy of breast cancer. Journal of experimental & clinical cancer research : CR. 2013;32:88.

Cammarota F, Giugliano FM, Iadanza L, et al. Hypofractionated breast cancer radiotherapy. Helical tomotherapy in supine position or classic 3DConformal radiotherapy in prone position: Which is better? Anticancer Research. 2014;34:1233-8.

Catli S, Demircioglu F, Kilic D, Erbas G. The effect of whole breast irradiation with coplanar three field technique in breast cancer patients onto hearth and LAD doses. [Turkish]. Turk Onkoloji Dergisi. 2014;29:148-56.

Caudrelier JM, Meng J, Esche B, Grimard L, Ruddy T, Amjadi K. IMRT sparing of normal tissues in locoregional treatment of breast cancer. Radiat Oncol. 2014;9:1-7.

Cendales R, Vasquez J, Arbelaez JC, et al. Intensity modulated radiotherapy (IMRT) with simultaneous integrated boost (SIB) in a patient with left breast cancer and pectus excavatum. Clinical and Translational Oncology. 2012;14:747-54.

Chamunyonga C. The impact of inter-fraction set-up errors on the probability of pulmonary and cardiac complication in left-sided breast cancer patients. Journal of Radiotherapy in Practice. 2014;13:393-402.

Chan TY, Tan PW, Tan CW, Tang JI. Assessing radiation exposure of the left anterior descending artery, heart and lung in patients with left breast cancer: A dosimetric comparison between multicatheter accelerated partial breast irradiation and whole breast external beam radiotherapy. Radiother Oncol. 2015;117:459-66.

Chen JL, Cheng JC, Kuo SH, Chan HM, Huang YS, Chen YH. Prone breast forward intensity-modulated radiotherapy for Asian women with early left breast cancer: factors for cardiac sparing and clinical outcomes. J Radiat Res. 2013;54:899-908.

Chi F, Wu S, Zhou J, et al. Dosimetric comparison of moderate deep inspiration breath-hold and free-breathing intensity-modulated radiotherapy for left-sided breast cancer. Cancer/Radiotherapie. 2015;19:180-6.

Chung MJ, Kim SH, Lee JH, Suh YJ. A dosimetric comparative analysis of TomoDirect and three-dimensional conformal radiotherapy in early breast cancer. Journal of Breast Cancer. 2015;18:57-62.

Chung Y, Kim JW, Shin KH, et al. Dummy run of quality assurance program in a phase 3 randomized trial investigating the role of internal mammary lymph node irradiation in breast cancer patients: Korean Radiation Oncology Group 08-06 study. Int J Radiat Oncol Biol Phys. 2015;91:419-26.

Cilla S, Digesu C, Macchia G, et al. Clinical implications of different calculation algorithms in breast radiotherapy: A comparison between pencil beam and collapsed cone convolution. Physica Medica. 2014;30:473-81.

Cilla S, Kigula-Mugambe J, Digesu C, et al. Forward-planned intensity modulated radiation therapy using a cobalt source: A dosimetric study in breast cancer. Journal of Medical Physics. 2013;38:125-31.

Comsa D, Barnett E, Le K, et al. Introduction of moderate deep inspiration breath hold for radiation therapy of left breast: Initial experience of a regional cancer center. Practical Radiation Oncology. 2014;4:298-305.

Cuaron JJ, Chon B, Tsai H, et al. Early toxicity in patients treated with postoperative proton therapy for locally advanced breast cancer. Int J Radiat Oncol Biol Phys. 2015;92:284-91.

Dinclogan F, Beyzadeoglu M, Sager O, et al. Dosimetric evaluation of critical organs at risk in mastectomized left-sided breast cancer radiotherapy using breath hold techniques. Tumori. 2013;99:76-82.

Dogan MH, Zincircioglu SB, Aydinol M. Research on different techniques in breast cancer radiotherapy. Współczesna Onkologia/Contemporary Oncology. 2013;17:291-7.

Donovan EM, Brooks C, Mitchell RA, et al. The effect of image guidance on dose distributions in breast boost radiotherapy. Clinical Oncology. 2014;26:671-6.

Ducournau A, Lagarde P, Henriques de Figueiredo B, et al. Post-surgery radiation therapy for a patient with osteogenesis imperfecta: About one case. Cancer/Radiotherapie. 2014;18:132-5.

Edvardsson A, Nilsson MP, Amptoulach S, Ceberg S. Comparison of doses and NTCP to risk organs with enhanced inspiration gating and free breathing for left-sided breast cancer radiotherapy using the AAA algorithm. Radiat Oncol. 2015;10:84.

Ekambaram V, Velayudham R, Swaminathan S, Loganathan P, Swaminathan V. Planning aspects of volumetric modulated arc therapy and intensity modulated radio therapy in carcinoma left breast - A comparative study. Asian Pacific Journal of Cancer Prevention. 2015;16:1633-6.

Eldredge-Hindy H, Lockamy V, Crawford A, et al. Active Breathing Coordinator reduces radiation dose to the heart and preserves local control in patients with left breast cancer: Report of a prospective trial. Practical Radiation Oncology. 2015;5:4-10.

Erven K, Petillion S, Weltens C, et al. Conformal locoregional breast irradiation with an oblique parasternal photon field technique. Med Dosim. 2011;36:28-34.

Essers M, Osman SOS, Hol S, Donkers T, Poortmans PM. Accelerated partial breast irradiation (APBI): Are breath-hold and volumetric radiation therapy techniques useful? Acta Oncol. 2014;53:788-94.

Fan L, Luo Y, Xu J, He L, Wang J, Du X. A dosimetry study precisely outlining the heart substructure of left breast cancer patients using intensity-modulated radiation therapy. J Appl Clin Med Phys. 2014;15:4624.

Farace P, Zucca S, Solla I, et al. Planning hybrid intensity modulated radiation therapy for whole-breast irradiation. Int J Radiat Oncol Biol Phys. 2012;84:e115-22.

Fekete G, Újhidy D, Együd Z, et al. Partial breast radiotherapy with simple teletherapy techniques. Med Dosim. 2015;40:290-5.

Fernandez-Lizarbe E, Montero A, Polo A, et al. Pilot study of feasibility and dosimetric comparison of prone versus supine breast radiotherapy. Clinical & translational oncology : official publication of the Federation of Spanish Oncology Societies and of the National Cancer Institute of Mexico. 2013;15:450-9.

Flejmer AM, Dohlmar F, Nilsson M, Stenmarker M, Dasu A. Analytical anisotropic algorithm versus pencil beam convolution for treatment planning of breast cancer: Implications for target coverage and radiation burden of normal tissue. Anticancer Research. 2015;35:2841-8.

Flejmer AM, Josefsson D, Nilsson M, Stenmarker M, Dasu A. Clinical implications of the ISC technique for breast cancer radiotherapy and comparison with clinical recommendations. Anticancer Research. 2014;34:3563-8.

Franco P, Zeverino M, Migliaccio F, et al. Intensity-modulated adjuvant whole breast radiation delivered with static angle tomotherapy (TomoDirect): A prospective case series. Journal of Cancer Research and Clinical Oncology. 2013;139:1927-36.

Gauer T, Engel K, Kiesel A, Albers D, Rades D. Comparison of electron IMRT to helical photon IMRT and conventional photon irradiation for treatment of breast and chest wall tumours. Radiother Oncol. 2010;94:313-8.

Capezzali G, Kirova YM, Costa E, et al. Left Breast Cancer Treated in Isocentric Lateral Decubitus (ILD) Position: An Alternative Technique Sparing Organs at Risk (OAR). Global Journal of Breast Cancer Research. 2013;1:53-7.

Giraud P, Djadi-Prat J, Morelle M, et al. Contribution of respiratory gating techniques for optimization of breast cancer radiotherapy. Cancer investigation. 2012;30:323-30.

Guenzi M, Bosetti D, Lamanna G, et al. Novel 10-fraction breast irradiation in prone and supine position: Technical, dosimetric and clinical evaluation. Tumori. 2015;101:154-60.

Guilbert P, Gaillot-Petit N, Vieren L, Nguyen TD. [Conventional 2D and monoisocentric 3D techniques in breast and lymphatic irradiation: a dosimetric comparison]. Cancer Radiother. 2012;16:473-8.

Gultekin M, Karabuga M, Yildiz F, et al. Comparison of chest wall and lymphatic radiotherapy techniques in patients with left breast carcinoma. Meme Sagligi Dergisi / Journal of Breast Health. 2014;10:106-10.

Gursel B, Meydan D, Ozbek N, Ofluoglu T. Dosimetric comparison of three different external beam whole breast irradiation techniques. Adv Ther. 2011;28:1114-25.

Haciislamoglu E, Colak F, Canyilmaz E, et al. Dosimetric comparison of left-sided whole-breast irradiation with 3DCRT, forward-planned IMRT, inverse-planned IMRT, helical tomotherapy, and volumetric arc therapy. Physica Medica. 2015;31:360-7.

Hashimoto H, Omura M, Matsui K, et al. Tangent field technique of TomoDirect improves dose distribution for whole-breast irradiation. J Appl Clin Med Phys. 2015;16:225-32.

Hayden AJ, Rains M, Tiver K. Deep inspiration breath hold technique reduces heart dose from radiotherapy for left-sided breast cancer. J Med Imaging Radiat Oncol. 2012;56:464-72.

He Z, Wu S, Zhou J, et al. Accelerated partial breast irradiation with intensity-modulated radiotherapy is feasible for Chinese breast cancer patients. Journal of Breast Cancer. 2014;17:256-64.

He ZY, Chi F, Li FY, Wang JJ, Wu SG, Guan XX. Dosimetry of moderate deep inspiration breath hold achieved using an active breathing control device in the target of forward whole-breast intensity-modulated radiotherapy after breast conserving surgery in breast cancer. Chinese Journal of Cancer Prevention and Treatment. 2011;18:1273-6.

Henzen D, Manser P, Frei D, et al. Beamlet based direct aperture optimization for MERT using a photon MLC. Med Phys. 2014;41:121711.

Hepp R, Ammerpohl M, Morgenstern C, et al. Deep inspiration breath-hold (DIBH) radiotherapy in left-sided breast cancer: Dosimetrical comparison and clinical feasibility in 20 patients. Strahlentherapie und Onkologie. 2015;191:710-6.

Heymann S, Verstraet R, Pichenot C, et al. [Intensity modulation in breast radiotherapy: development of an innovative field-in-field technique at Institut Gustave-Roussy]. Cancer Radiother. 2011;15:663-9.

Hijal T, Fournier-Bidoz N, Castro-Pena P, et al. Simultaneous integrated boost in breast conserving treatment of breast cancer: A dosimetric comparison of helical tomotherapy and three-dimensional conformal radiotherapy. Radiother Oncol. 2010;94:300-6.

Hjelstuen MH, Mjaaland I, Vikstrom J, Dybvik KI. Radiation during deep inspiration allows loco-regional treatment of left breast and axillary-, supraclavicular- and internal mammary lymph nodes without compromising target coverage or dose restrictions to organs at risk. Acta Oncol. 2011;51:333-44.

Jagsi R, Moran J, Marsh R, Masi K, Griffith KA, Pierce LJ. Evaluation of Four Techniques Using Intensity-Modulated Radiation Therapy for Comprehensive Locoregional Irradiation of Breast Cancer. Int J Radiat Oncol Biol Phys. 2010;78:1594-603.

Jin GH, Chen LX, Deng XW, Liu XW, Huang Y, Huang XB. A comparative dosimetric study for treating left-sided breast cancer for small breast size using five different radiotherapy techniques: conventional tangential field, filed-in-filed, Tangential-IMRT, Multi-beam IMRT and VMAT. Radiat Oncol. 2013;8:89.

Johansen S, Vikstrom J, Hjelstuen MH, Mjaaland I, Dybvik KI, Olsen DR. Dose evaluation and risk estimation for secondary cancer in contralateral breast and a study of correlation between thorax shape and dose to organs at risk following tangentially breast irradiation during deep inspiration breath-hold and free breathing. Acta Oncol. 2011;50:563-8.

Joosten A, Matzinger O, Jeanneret-Sozzi W, Bochud F, Moeckli R. Evaluation of organ-specific peripheral doses after 2-dimensional, 3-dimensional and hybrid intensity modulated radiation therapy for breast cancer based on Monte Carlo and convolution/superposition algorithms: implications for secondary cancer risk assessment. Radiother Oncol. 2013;106:33-41.

Jost V, Kretschmer M, Sabatino M, et al. Heart dose reduction in breast cancer treatment with simultaneous integrated boost: Comparison of treatment planning and dosimetry for a novel hybrid technique and 3D-CRT. Strahlentherapie und Onkologie. 2015;191:734-41.

Kainz K, White J, Chen GP, Hermand J, Li XA. Simultaneous irradiation of the breast and regional lymph nodes in prone position using helical tomotherapy. Br J Radiol. 2012;85:899-905.

Khan F, Craft D. Three-dimensional conformal planning with low-segment multicriteria intensity modulated radiation therapy optimization. Practical Radiation Oncology. 2015;5:e103-e11.

Kim H, Bae H, Lee MY, et al. Analysis of predictive factors for lung injury after forward-planned intensity-modulated radiotherapy in whole breast irradiation. Journal of Breast Cancer. 2014;17:69-75.

Kirby AM, Evans PM, Donovan EM, Convery HM, Haviland JS, Yarnold JR. Prone versus supine positioning for whole and partial-breast radiotherapy: A comparison of non-target tissue dosimetry. Radiother Oncol. 2010;96:178-84.

Kirova YM, Hijal T, Campana F, et al. Whole breast radiotherapy in the lateral decubitus position: A dosimetric and clinical solution to decrease the doses to the organs at risk (OAR). Radiother Oncol. 2014;110:477-81.

Koh V, Tang JI, Choo BA, et al. Body mass index and patient CT measurements as a predictor of benefit of intensity-modulated radiotherapy to the supraclavicular fossa. OncoTargets and Therapy. 2013;6:1701-6.

Krengli M, Masini L, Caltavuturo T, et al. Prone versus supine position for adjuvant breast radiotherapy: A prospective study in patients with pendulous breasts. Radiation Oncology. 2013;8:232.

Lakosi F, Gulyban A, Janvary L, et al. Respiratory Motion, Anterior Heart Displacement and Heart Dosimetry: Comparison Between Prone (Pr) and Supine (Su) Whole Breast Irradiation. Pathology and Oncology Research. 2015;21:1051-8.

Lamberth F, Guilbert P, Gaillot-Petit N, Champagne C, Looten-Vieren L, Nguyen TD. Potential indications for helical tomotherapy in breast cancers. Cancer/Radiotherapie. 2014;18:7-14.

Lee HY, Chang JS, Lee IJ, et al. The deep inspiration breath hold technique using Abches reduces cardiac dose in patients undergoing left-sided breast irradiation. Radiat Oncol J. 2013;31:239-46.

Lee JA, Yoon WS, Chung SY, et al. Can intensity-modulated radiation therapy spare the central flapped area while encompassing the target volume in radiotherapy after immediate breast reconstruction? Journal of Medical Imaging and Radiation Oncology. 2013;57:595-602.

Li R, Xing L, Horst KC, Bush K. Nonisocentric treatment strategy for breast radiation therapy: A proof of concept study. Int J Radiat Oncol Biol Phys. 2014;88:920-6.

Liljegren A, Unukovych D, Gagliardi G, et al. No difference in dose distribution in organs at risk in postmastectomy radiotherapy with or without breast implant reconstruction. Radiat Oncol. 2014;9:14.

Lin JF, Yeh DC, Yeh HL, Chang CF, Lin JC. Dosimetric comparison of hybrid volumetric-modulated arc therapy, volumetric-modulated arc therapy, and intensity-modulated radiation therapy for left-sided early breast cancer. Med Dosim. 2015;40:262-7.

Lin LL, Vennarini S, Dimofte A, et al. Proton beam versus photon beam dose to the heart and left anterior descending artery for left-sided breast cancer. Acta Oncol. 2015;54:1032-9.

Lin Y, Wang B. Dosimetric absorption of intensity-modulated radiotherapy compared with conventional radiotherapy in breast-conserving surgery. Oncology Letters. 2015;9:9-14.

Liu ZM, Ge XL, Chen JY, et al. Adjuvant radiotherapy after breast conserving treatment for breast cancer: A dosimetric comparison between volumetric modulated arc therapy and intensity modulated radiotherapy. Asian Pacific Journal of Cancer Prevention. 2015;16:3257-65.

Lymberis SC, deWyngaert JK, Parhar P, et al. Prospective assessment of optimal individual position (prone versus supine) for breast radiotherapy: volumetric and dosimetric correlations in 100 patients. Int J Radiat Oncol Biol Phys. 2012;84:902-9.

Ma C, Zhang W, Lu J, et al. Dosimetric Comparison and Evaluation of Three Radiotherapy Techniques for Use after Modified Radical Mastectomy for Locally Advanced Left-sided Breast Cancer. Scientific Reports. 2015;5:122274.

Ma J, Li J, Xie J, et al. Post mastectomy linac IMRT irradiation of chest wall and regional nodes: dosimetry data and acute toxicities. Radiat Oncol. 2013;8:81.

MacDonald SM, Patel SA, Hickey S, et al. Proton therapy for breast cancer after mastectomy: early outcomes of a prospective clinical trial. Int J Radiat Oncol Biol Phys. 2013;86:484-90.

Majumdar D, Mohammed SS, Naseer MA, et al. Respiratory gated simultaneous integrated boost-intensity modulated radiotherapy (SIB-IMRT) after breast conservative surgery for carcinoma of the breast: The Salmaniya Medical complex experience. Gulf J Oncolog. 2011;10:53-9.

Mancosu P, Reggiori G, Alongi F, et al. Total monitor units influence on plan quality parameters in volumetric modulated arc therapy for breast case. Physica Medica. 2014;30:296-300.

Mansouri S, Naim A, Glaria L, Marsiglia H. Dosimetric evaluation of 3-D conformal and intensity-modulated radiotherapy for breast cancer after conservative surgery. Asian Pacific Journal of Cancer Prevention. 2014;15:4727-32.

Mast ME, van Kempen-Harteveld L, Heijenbrok MW, et al. Left-sided breast cancer radiotherapy with and without breath-hold: does IMRT reduce the cardiac dose even further? Radiother Oncol. 2013;108:248-53.

Mast ME, Vredeveld EJ, Credoe HM, et al. Whole breast proton irradiation for maximal reduction of heart dose in breast cancer patients. Breast cancer research and treatment. 2014;148:33-9.

Mavroidis P, Ferreira BC, Lopes MDC. Response-probability volume histograms and iso-probability of response charts in treatment plan evaluation. Med Phys. 2011;38:2382-97.

Mayadagli A, Gocen E, Aksu A, et al. Evaluation of conventional radiotherapy and conformal radiotherapy technique in terms of dose distribution following breast conserving therapy. Turkiye Klinikleri Journal of Medical Sciences. 2011;31:1514-20.

Mezenski P, Galecki J, Zawadzka A, Bulski W, Kukolowicz P. Comparison of 3D-CRT and IMRT techniques in the radiotherapy of breast cancer patients after breast sparing surgery with and without the lymph nodes involvment.Nowotwory Journal of Oncology. 2012;62:423-30.

Michalski A, Atyeo J, Cox J, Rinks M, Morgia M, Lamoury G. A dosimetric comparison of 3D-CRT, IMRT, and static tomotherapy with an SIB for large and small breast volumes. Med Dosim. 2014;39:163-8.

Mulliez T, Speleers B, Madani I, De Gersem W, Veldeman L, De Neve W. Whole breast radiotherapy in prone and supine position: is there a place for multi-beam IMRT? Radiat Oncol. 2013;8:151.

Mulliez T, Speleers B, Mahjoubi K, et al. Prone left-sided whole-breast irradiation: Significant heart dose reduction using end-inspiratory versus end-expiratory gating. Cancer/Radiotherapie. 2014;18:672-7.

Mulliez T, Veldeman L, Speleers B, et al. Heart dose reduction by prone deep inspiration breath hold in left-sided breast irradiation. Radiother Oncol. 2014;114:79-84.

Mulliez T, Veldeman L, van Greveling A, et al. Hypofractionated whole breast irradiation for patients with large breasts: a randomized trial comparing prone and supine positions. Radiother Oncol. 2013;108:203-8.

Murofushi KN, Oguchi M, Gosho M, Kozuka T, Sakurai H. Radiation-induced bronchiolitis obliterans organizing pneumonia (BOOP) syndrome in breast cancer patients is associated with age. Radiat Oncol. 2015;10:103.

Mydin AR, Gaffney H, Bergman A, et al. Does a three-field electron/minitangent photon technique offer dosimetric advantages to a multifield, photon-only technique for accelerated partial breast irradiation? American Journal of Clinical Oncology: Cancer Clinical Trials. 2010;33:336-40.

Nagar H, Zhou L, Biritz B, et al. Is there a tradeoff in using modified high tangent field radiation for treating an undissected node-positive Axilla? Clinical Breast Cancer. 2014;14:109-13.

Nakamura N, Takahashi O, Kamo M, et al. Effects of geometrical uncertainties on whole breast radiotherapy: A comparison of four different techniques. Journal of Breast Cancer. 2014;17:157-60.

Ng J, Shuryak I, Xu Y, Clifford Chao KS, Brenner DJ, Burri RJ. Predicting the risk of secondary lung malignancies associated with whole-breast radiation therapy. Int J Radiat Oncol Biol Phys. 2012;83:1101-6.

Nichols GP, Fontenot JD, Gibbons JP, Sanders ME. Evaluation of volumetric modulated arc therapy for postmastectomy treatment. Radiat Oncol. 2014;9:66.

Nicolini G, Fogliata A, Clivio A, Vanetti E, Cozzi L. Planning strategies in volumetric modulated are therapy for breast. Med Phys. 2011;38:4025-31.

Nitsche M, Temme N, Forster M, Reible M, Hermann RM. Tangential vs. defined radiotherapy in early breast cancer treatment without axillary lymph node dissection. A comparative study. Strahlentherapie und Onkologie. 2014;190:715-21.

Onal C, Sonmez A, Arslan G, et al. Dosimetric comparison of the field-in-field technique and tangential wedged beams for breast irradiation. Jpn J Radiol. 2012;30:218-26.

Opp D, Forster K, Li W, Zhang G, Harris EE. Evaluation of bolus electron conformal therapy compared with conventional techniques for the treatment of left chest wall postmastectomy in patients with breast cancer. Med Dosim. 2013;38:448-53.

Osei E, Darko J, Fleck A, et al. Dosimetric evaluation of whole-breast radiation therapy: Clinical experience. Med Dosim. 2015;40:355-65.

Osman SOS, Hol S, Poortmans PM, Essers M. Volumetric modulated arc therapy and breath-hold in image-guided locoregional left-sided breast irradiation. Radiother Oncol. 2014;112:17-22.

Palta M, Yoo S, Adamson JD, Prosnitz LR, Horton JK. Preoperative single fraction partial breast radiotherapy for early-stage breast cancer. Int J Radiat Oncol Biol Phys. 2012;82:37-42.

Pasler M, Georg D, Bartelt S, Lutterbach J. Node-positive left-sided breast cancer: does VMAT improve treatment plan quality with respect to IMRT? Strahlentherapie und Onkologie. 2013;189:380-6.

Pasler M, Lutterbach J, Bjornsgard M, Reichmann U, Bartelt S, Georg D. VMAT techniques for lymph node-positive left sided breast cancer. Z Med Phys. 2015;25:104-11.

Peters S, Schiefer H, Plasswilm L. A treatment planning study comparing Elekta VMAT and fixed field IMRT using the varian treatment planning system eclipse. Radiat Oncol. 2014;9:153.

Peulen H, Hanbeukers B, Boersma L, et al. Forward Intensity-Modulated Radiotherapy Planning in Breast Cancer To Improve Dose Homogeneity: Feasibility of Class Solutions. Int J Radiat Oncol Biol Phys. 2012;82:394-400.

Pignol JP, Keller BM, Ravi A. Doses to internal organs for various breast radiation techniques--implications on the risk of secondary cancers and cardiomyopathy. Radiat Oncol. 2011;6:5.

Pili G, Grimaldi L, Fidanza C, et al. Geometric and Dosimetric Approach to Determine Probability of Late Cardiac Mortality in Left Tangential Breast Irradiation: Comparison Between Wedged Beams and Field-in-Field Technique. Int J Radiat Oncol Biol Phys. 2011;81:894-900.

Popescu CC, Olivotto IA, Beckham WA, et al. Volumetric Modulated Arc Therapy Improves Dosimetry and Reduces Treatment Time Compared To Conventional Intensity-Modulated Radiotherapy for Locoregional Radiotherapy of Left-Sided Breast Cancer and Internal Mammary Nodes. Int J Radiat Oncol Biol Phys. 2010;76:287-95.

Qi XS, Liu TX, Liu AK, et al. Left-sided breast cancer irradiation using rotational and fixed-field radiotherapy. Med Dosim. 2014;39:227-34.

Qiu J, Liu Z, Yang B, Hou X, Zhang F. Low-dose-area-constrained helical TomoTherapy-based whole breast radiotherapy and dosimetric comparison with tangential field-in-field IMRT. Biomed Res Int. 2013;2013:513708.

Qiu JJ, Chang Z, Horton JK, Wu QRJ, Yoo S, Yin FF. Dosimetric comparison of 3D conformal, IMRT, and V-MAT techniques for accelerated partial-breast irradiation (APBI). Med Dosim. 2014;39:152-8.

Qiu JJ, Chang Z, Wu QJ, Yoo S, Horton J, Yin FF. Impact of volumetric modulated arc therapy technique on treatment with partial breast irradiation. Int J Radiat Oncol Biol Phys. 2010;78:288-96.

Reardon KA, Read PW, Morris MM, Reardon MA, Geesey C, Wijesooriya K. A comparative analysis of 3D conformal deep inspiratory-breath hold and free-breathing intensity-modulated radiation therapy for left-sided breast cancer. Med Dosim. 2013;38:190-5.

Register S, Takita C, Reis I, Zhao W, Amestoy W, Wright J. Deep inspiration breath-hold technique for left-sided breast cancer: An analysis of predictors for organ-at-risk sparing. Med Dosim. 2015;40:89-95.

Rudat V, Alaradi AA, Mohamed A, Ai-Yahya K, Altuwaijri S. Tangential beam IMRT versus tangential beam 3D-CRT of the chest wall in postmastectomy breast cancer patients: A dosimetric comparison. Radiat Oncol. 2011;6:26.

Rudra S, Al-Hallaq HA, Feng C, Chmura SJ, Hasan Y. Effect of RTOG breast/chest wall guidelines on dose-volume histogram parameters. J Appl Clin Med Phys. 2014;15:127-37.

Sager O, Beyzadeoglu M, Dincoglan F, et al. The role of active breathing control-moderate deep inspiration breath-hold (ABC-mDIBH) usage in non-mastectomized left-sided breast cancer radiotherapy: A dosimetric evaluation. UHOD-International Journal of Hematology and Oncology 2012;22:147-55.

Saha A, Mahata A, Shrimali R, Achari R, Mallick I, Chatterjee S. Helical tomotherapy based intensity modulated radiotherapy for the management of difficult clinical situations in breast cancer. Clinical Cancer Investigation Journal. 2015;4:543-7.

Sakumi A, Shiraishi K, Onoe T, et al. Single-arc volumetric modulated arc therapy planning for left breast cancer and regional nodes. J Radiat Res. 2012;53:151-3.

Salem A, Mohamad I, Dayyat A, et al. Combined photon-electron beams in the treatment of the supraclavicular lymph nodes in breast cancer: A novel technique that achieves adequate coverage while reducing lung dose. Med Dosim. 2015;40:210-7.

Sasaoka M, Futami T. Dosimetric evaluation of whole breast radiotherapy using field-in-field technique in early-stage breast cancer. Int J Clin Oncol. 2011;16:250-6.

Sas-Korczynska B, Sladowska A, Rozwadowska-Bogusz B, et al. Comparison between intensity modulated radiotherapy (IMRT) and 3D tangential beams technique used in patients with early-stage breast cancer who received breast-conserving therapy. Reports of Practical Oncology and Radiotherapy. 2010;15:79-86.

Schubert LK, Gondi V, Sengbusch E, et al. Dosimetric comparison of left-sided whole breast irradiation with 3DCRT, forward-planned IMRT, inverse-planned IMRT, helical tomotherapy, and topotherapy. Radiother Oncol. 2011;100:241-6.

Scorsetti M, Alongi F, Fogliata A, et al. Phase I-II study of hypofractionated simultaneous integrated boost using volumetric modulated arc therapy for adjuvant radiation therapy in breast cancer patients: a report of feasibility and early toxicity results in the first 50 treatments. Radiat Oncol. 2012;7:145.

Semaniak A, Jodkiewicz Z, Skowronska-Gardas A. Segmented photon beams technique for irradiation of postmastectomy patients. reports of practical oncology and radiotherapy. 2012;17:85-92.

Shiau AC, Hsieh CH, Tien HJ, et al. Left-sided whole breast irradiation with hybrid-IMRT and helical tomotherapy dosimetric comparison. BioMed research international. 2014;2014:741326.

Sonmez A, Onal C, Sonmez S, et al. Effects of setup errors on dose distribution for tangential wedge field and field-in-field techniques during breast irradiation. UHOD - Uluslararasi Hematoloji-Onkoloji Dergisi. 2014;24:130-8.

Stillie AL, Kron T, Herschtal A, et al. Does inverse-planned intensity-modulated radiation therapy have a role in the treatment of patients with left-sided breast cancer? J Med Imaging Radiat Oncol. 2011;55:311-9.

Subramaniam S, Thirumalaiswamy S, Srinivas C, et al. Chest wall radiotherapy with volumetric modulated arcs and the potential role of flattening filter free photon beams. Strahlentherapie und Onkologie. 2012;188:484-90.

Sun LM, Meng FY, Yang TH, Tsao MJ. Field-in-field plan does not improve the dosimetric outcome compared with the wedged beams plan for breast cancer radiotherapy. Med Dosim. 2014;39:79-82.

Sung K, Lee KC, Ahn SH, Lee SH, Choi J. Cardiac dose reduction with breathing adapted radiotherapy using self respiration monitoring system for left-sided breast cancer. Radiation Oncology Journal. 2014;32:84-94.

Swamy ST, Radha CA, Kathirvel M, Arun G, Subramanian S. Feasibility study of deep inspiration breath-hold based volumetric modulated arc therapy for locally advanced left sided breast cancer patients. Asian Pacific Journal of Cancer Prevention. 2014;15:9033-8.

Swanson T, Grills IS, Ye H, et al. Six-year Experience Routinely Using Moderate Deep Inspiration Breath-hold for the Reduction of Cardiac Dose in Left-sided Breast Irradiation for Patients With Early-stage or Locally Advanced Breast Cancer. American journal of clinical oncology. 2012;36:24-30.

Tan W, Liu D, Xue C, et al. Anterior Myocardial Territory May Replace the Heart as Organ at Risk in Intensity-Modulated Radiotherapy for Left-Sided Breast Cancer. Int J Radiat Oncol Biol Phys. 2012;82:1689-97.

Tan W, Wang X, Qiu D, et al. Dosimetric Comparison of Intensity-Modulated Radiotherapy Plans, With or Without Anterior Myocardial Territory and Left Ventricle as Organs at Risk, in Early-Stage Left-Sided Breast Cancer Patients. Int J Radiat Oncol Biol Phys. 2011;81:1544-51.

Tanaka H, Hayashi S, Kajiura Y, et al. Evaluation of the field-in-field technique with lung blocks for breast tangential radiotherapy. Nagoya Journal of Medical Science. 2015;77:339-45.

Tang X, Cullip T, Dooley J, et al. Dosimetric effect due to the motion during deep inspiration breath hold for left-sided breast cancer radiotherapy. J Appl Clin Med Phys. 2015;16:91-9.

Tanguturi SK, Lyatskaya Y, Chen Y, et al. Prospective assessment of deep inspiration breath-hold using 3-dimensional surface tracking for irradiation of left-sided breast cancer. Practical Radiation Oncology. 2015;5:358-65.

Teh AY, Walsh L, Purdie TG, et al. Concomitant intensity modulated boost during whole breast hypofractionated radiotherapy--a feasibility and toxicity study. Radiother Oncol. 2012;102:89-95.

Thorsen LBJ, Thomsen MS, Berg M, et al. CT-planned internal mammary node radiotherapy in the DBCG-IMN study: Benefit versus potentially harmful effects. Acta Oncol. 2014;53:1027-34.

Trifiletti DM, Wijesooriya K, Moyer G, et al. Intensity-modulated radiotherapy versus three-dimensional conformal radiotherapy during deep inspiratory breath hold for left-sided whole-breast irradiation: a comparative analysis. Journal of Radiotherapy in Practice. 2015;15:99-106.

Trofimova OP, Tkachev SI, Kostina NP, et al. Radiation pneumonitis in the treatment of early-stage breast cancer. Voprosy onkologii. 2015;61:116-20.

Tsai PF, Lin SM, Lee SH, et al. The feasibility study of using multiple partial volumetric-modulated arcs therapy in early stage left-sided breast cancer patients. J Appl Clin Med Phys. 2012;13:3806.

Tsuchiya K, Kinoshita R, Shimizu S, et al. Dosimetric comparison between intensity-modulated radiotherapy and standard wedged tangential technique for whole-breast radiotherapy in Asian women with relatively small breast volumes. Radiological Physics and Technology. 2014;7:67-72.

Uhl M, Sterzing F, Habl G, et al. Breast cancer and funnel chest : Comparing helical tomotherapy and three-dimensional conformal radiotherapy with regard to the shape of pectus excavatum. Strahlentherapie und Onkologie. 2012; 188:127-35.

Vaegler S, Bratengeier K, Beckmann G, Flentje M. Conformal breast irradiation with the arm of the affected side parallel to the body. Strahlentherapie und Onkologie. 2014;190:100-5.

van der Laan HP, Dolsma WV, Schilstra C, et al. Limited benefit of inversely optimised intensity modulation in breast conserving radiotherapy with simultaneously integrated boost. Radiother Oncol. 2010; 94:307-12.

van der Laan HP, Korevaar EW, Dolsma WV, Maduro JH, Langendijk JA. Minimising contralateral breast dose in post-mastectomy intensity-modulated radiotherapy by incorporating conformal electron irradiation. Radiother Oncol. 2010;94:307-12.

Van Parijs H, Miedema G, Vinh-Hung V, et al. Short course radiotherapy with simultaneous integrated boost for stage I-II breast cancer, early toxicities of a randomized clinical trial. Radiat Oncol. 2012;7:80.

Varga Z, Cserhati A, Rarosi F, et al. Individualized positioning for maximum heart protection during breast irradiation. Acta Oncologica. 2014;53:58-64.

Vera R, Trombetta M, Mukhopadhyay ND, Packard M, Arthur D. Long-term cosmesis and toxicity following 3-dimensional conformal radiation therapy in the delivery of accelerated partial breast irradiation. Practical Radiation Oncology. 2014;4:147-52.

Verhoeven K, Sweldens C, Petillion S, Laenen A, Peeters S, Janssen H, et al. Breathing adapted radiation therapy in comparison with prone position to reduce the doses to the heart, left anterior descending coronary artery, and contralateral breast in whole breast radiation therapy. Practical Radiation Oncology. 2014;4:123-9.

Vikstrom J, Hjelstuen MH, Mjaaland I, Dybvik KI. Cardiac and pulmonary dose reduction for tangentially irradiated breast cancer, utilizing deep inspiration breath-hold with audio-visual guidance, without compromising target coverage. Acta Oncol. 2011;50:42-50.

Viren T, Heikkila J, Myllyoja K, Koskela K, Lahtinen T, Seppala J. Tangential volumetric modulated arc therapy technique for left-sided breast cancer radiotherapy. Radiation Oncology. 2015;10:79.

Vrana D, Cwiertka K, Lukesova L. [Breast cancer adjuvant radiotherapy using active breathing control-moderate inspiration breath-hold - a case report]. Klinicka onkologie : casopis Ceske a Slovenske onkologicke spolecnosti. 2013;26:143-6.

Wang J, Li X, Deng Q, et al. Postoperative radiotherapy following mastectomy for patients with left-sided breast cancer: A comparative dosimetric study. Medical Dosimetry. 2015;40:190-4.

Wang W, Purdie TG, Rahman M, Marshall A, Liu FF, Fyles A. Rapid automated treatment planning process to select breast cancer patients for active breathing control to achieve cardiac dose reduction. Int J Radiat Oncol Biol Phys. 2012;82:386-93.

Wang W, Li JB, Hu HG, et al. Evaluation of dosimetric variance in whole breast forward planned IMRT. Journal of Radiation Research. 2013;54:755–61.

Wang X, Zhang X, Li X, et al. Accelerated partial-breast irradiation using intensity-modulated proton radiotherapy: do uncertainties outweigh potential benefits? Br J Radiol. 2013;86:20130176.

Wright P, Suilamo S, Lindholm P, Kulmala J. Isocentric integration of intensity-modulated radiotherapy with electron fields improves field junction dose uniformity in postmastectomy radiotherapy. Acta Oncologica. 2014;53:1019-26.

Wu S, He Z, Guo J, Li F, Lin Q, Guan X. Dosimetric comparison of normal structures associated with accelerated partial breast irradiation and whole breast irradiation delivered by intensity modulated radiotherapy for early breast cancer after breast conserving surgery. Clinical and Translational Oncology. 2014;16:69-76.

Wu S, Lai Y, He Z, et al. Dosimetric comparison of the simultaneous integrated boost in whole-breast irradiation after breast-conserving surgery: IMRT, IMRT plus an electron boost and VMAT. PLoS ONE. 2015;10.

Würschmidt F, Stoltenberg S, Kretschmer M, Petersen C. Incidental dose to coronary arteries is higher in prone than in supine whole breast irradiation: A dosimetric comparison in adjuvant radiotherapy of early stage breast cancer. Strahlentherapie und Onkologie. 2014;190:563-8.

Xie X, Ouyang S, Wang H, et al. Dosimetric comparison of left-sided whole breast irradiation with 3D-CRT, IP-IMRT and hybrid IMRT. Oncology Reports. 2014;31:2195-205.

Xu N, Ho MW, Li Z, Morris CG, Mendenhall NP. Can Proton Therapy Improve the Therapeutic Ratio in Breast Cancer Patients at Risk for Nodal Disease? American journal of clinical oncology. 2013.

Yang B, Wei XD, Zhao YT, Ma CM. Dosimetric evaluation of integrated IMRT treatment of the chest wall and supraclavicular region for breast cancer after modified radical mastectomy. Medical Dosimetry. 2014;39:185-9.

Yang DS, Lee JA, Yoon WS, et al. Whole breast irradiation for small-sized breasts after conserving surgery: Is the field-in-field technique optimal? Breast Cancer. 2014;21:162-9.

Yang W, McKenzie EM, Burnison M, et al. Clinical experience using a video-guided spirometry system for deep inhalation breath-hold radiotherapy of left-sided breast cancer. J Appl Clin Med Phys. 2015;16:251-60.

Yavas G, Yavas C, Acar H. Dosimetric comparison of whole breast radiotherapy using field in field and conformal radiotherapy techniques in early stage breast cancer. Iranian Journal of Radiation Research. 2012;10:131-8.

Yim J, Suttie C, Bromley R, Morgia M, Lamoury G. Intensity modulated radiotherapy and 3D conformal radiotherapy for whole breast irradiation: A comparative dosimetric study and introduction of a novel qualitative index for plan evaluation, the normal tissue index. Journal of Medical Radiation Sciences. 2015;62:184-91.

Yin Y, Chen J, Sun T, et al. Dosimetric research on intensity-modulated arc radiotherapy planning for left breast cancer after breast-preservation surgery. Med Dosim. 2012;37:287-92.

Zhang L, Yang ZZ, Chen XX, et al. Dose coverage of axillary level I-III areas during whole breast irradiation with simplified intensity modulated radiation therapy in early stage breast cancer patients. Oncotarget. 2015;6:18183-91.

Zhang Q, Chen JY, Hu WG, Guo XM. Modified partially wide tangents technique in post-mastectomy radiotherapy for patients with left-sided breast cancer. Chinese medical journal. 2010;123:2825-31.

Zhang Q, Yu XL, Hu WG, et al. Dosimetric comparison for volumetric modulated arc therapy and intensitymodulated radiotherapy on the left-sided chest wall and internal mammary nodes irradiation in treating post-mastectomy breast cancer. Radiology and Oncology. 2015;49:91-8.

Zhou G, Xu S, Dai X, et al. Clinical Dosimetric Study of Three Radiotherapy Techniques for Postoperative Breast Cancer: Helical Tomotherapy, IMRT, and 3D-CRT. Technol Cancer Res Treat. 2011;10:15-23.

	Number of regimens	V20ipsi Average* (SE)	V20ipsi Range†
**(a) Partial breast, Supine** (χ^2_3 = 7.9; p = 0.05)			
Protons	3	0.6 (0.4)	0.1– 1.4
Brachytherapy	1	1.0	1.0– 1.0
Oblique fields	4	2.5 (0.8)	1.0– 4.3
Tangents	2	2.5 (1.0)	1.6– 3.5
IMRT‡	5	3.0 (2.2)	0.2–11.8
**(a)**	**15**	**2.2 (0.8)**	**0.1–11.8**
**(b_1) Breast or chest wall, Supine** (χ^2_3 = 33.3; p < 0.001)			
Protons	4	8.3 (2.9)	2.8–16.5
IMRT	108	13.7 (0.6)	3.2–37.2
Tangents	99	13.8 (0.5)	4.7–27.7
Other§	3	25.3 (2.1)	21.1–27.7
**Subtotal**	**214**	**13.8 (0.4)**	**2.8–37.2**
**(b_2) Breast or chest wall, Prone**			
Tangents	4	1.3 (0.4)	0.5– 2.3
IMRT	3	3.8 (3.2)	0.2–10.2
**Subtotal**	**7**	**2.3 (1.4)**	**0.2–10.2**
**(b)**	**221**	**13.4 (0.4)**	**0.2–37.2**
**(c) Breast or chest wall with axilla or SCF, Supine** (χ^2_3 = 2710.3; p < 0.001)			
Protons	2	9.5 (0.5)	9.0–10.0
IMRT	14	21.8 (1.8)	11.0–34.1
Tangents	16	23.8 (1.4)	16.3–31.4
Oblique fields	2	35.5 (0.1)	35.4–35.6
**(c)**	**34**	**22.8 (1.3)**	**9.0–35.6**
**(d_1) Breast or chest wall with IMC + axilla or SCF, Supine** (χ^2_4 = 53.8; p < 0.001)			
Protons	4	18.4 (4.2)	12.7–31.0
IMRT	15	25.5 (1.5)	16.9–34.5
Wide tangents	20	34.0 (1.7)	18.9–44.5
**Subtotal**	**39**	**28.9 (1.4)**	**12.7–44.5**
**(d_2) Breast or chest wall with IMC + axilla or SCF, Prone**			
IMRT	2	12.4 (2.7)	9.7–15.1
**(d)**	**41**	**28.3 (1.4)**	**9.7–44.5**
**Total**	**311**	**15.9 (0.5)**	**0.1–44.5**

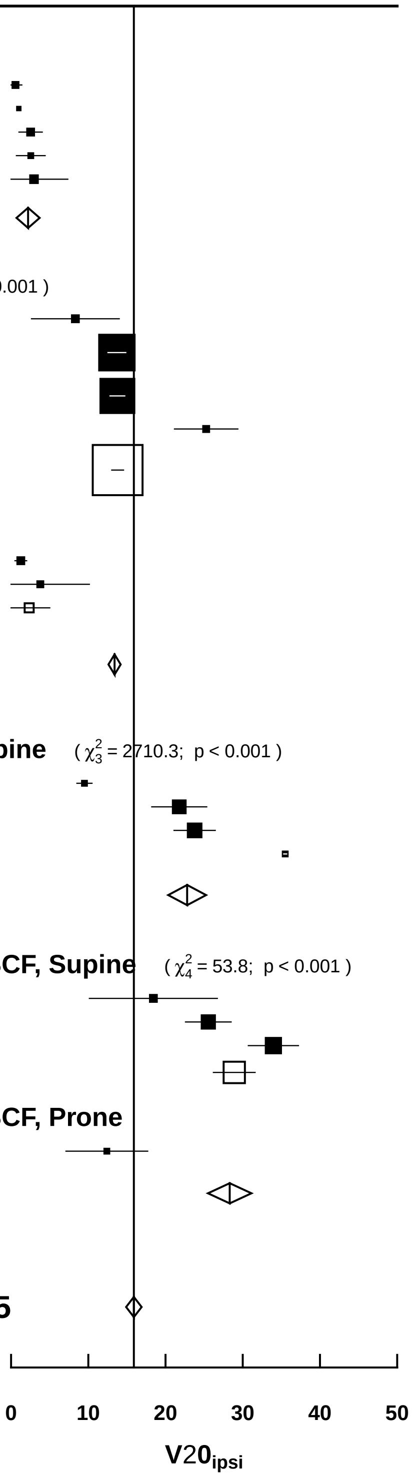

Figure E1. Percent volume of ipsilateral lung receiving 20 Gy or more ($V20_{ipsi}$) from breast cancer radiation therapy according to region irradiated and technique used. Regimens using breathing adaptation (e.g. deep inspiration breath hold) and studies of women with unfavourable anatomy were excluded.

∗ Average of ipsilateral $V20_{ipsi}$ for reported regimens.
† Range of ipsilateral $V20_{ipsi}$ for reported regimens.
‡ Static field IMRT and rotational IMRT are included jointly as IMRT.
§ Other techniques included two dynamic conformal arc therapy regimens and on unspecified 3D conformal regimen.
χ^2 and p are for heterogeneity.
Abbreviations: SE: standard error. CI: confidence interval. IMRT: intensity modulated radiotherapy. IMC: internal mammary chain. SCF: supraclavicular fossa.

	Number of regimens	Average* (SE)	Range†	V5ipsi Average & 95% CI
**(a) Partial breast, Supine** (χ^2_3 = 7.2; p = 0.07)				
Protons	3	4.6 (0.5)	3.5– 5.3	
Tangents	2	12.3 (6.1)	6.3–18.4	
IMRT‡	6	15.7 (6.6)	4.5–46.6	
Oblique fields	2	21.1 (10.0)	11.1–31.2	
Brachytherapy	1	23.9	23.9–23.9	
**(a)**	**14**	**14.2 (3.4)**	**3.5–46.6**	
**(b_1) Breast or chest wall, Supine** (χ^2_2 = 54.8; p < 0.001)				
Tangents	52	25.3 (1.5)	11.3–85.0	
Protons	1	34.4	34.4–34.4	
Other§	3	47.8 (9.7)	28.4–57.5	
IMRT	70	51.1 (3.3)	8.1–90.0	
**Subtotal**	**126**	**40.2 (2.2)**	**8.1–90.0**	
**(b_2) Breast or chest wall, Prone**				
IMRT	5	13.1 (11.2)	0.8–57.8	
**(b)**	**131**	**39.2 (2.2)**	**0.8–90.0**	
**(c) Breast or chest wall with axilla or SCF, Supine** (χ^2_1 = 3.7; p = 0.06)				
Tangents	3	37.3 (6.2)	30.3–49.6	
IMRT	3	56.0 (7.5)	45.0–70.4	
**(c)**	**6**	**46.6 (6.0)**	**30.3–70.4**	
**(d_1) Breast or chest wall with IMC + axilla or SCF, Supine** (χ^2_3 = 17.3; p < 0.001)				
Protons	1	50.0	50.0–50.0	
Wide tangents	8	60.7 (5.2)	45.3–84.5	
IMRT	12	71.5 (6.2)	41.2–99.3	
**Subtotal**	**21**	**66.4 (4.2)**	**41.2–99.3**	
**(d_2) Breast or chest wall with IMC + axilla or SCF, Prone**				
IMRT	2	53.1 (1.4)	51.7–54.5	
**(d)**	**23**	**65.2 (3.9)**	**41.2–99.3**	
**Total**	**174**	**40.9 (2.0)**	**0.8–99.3**	

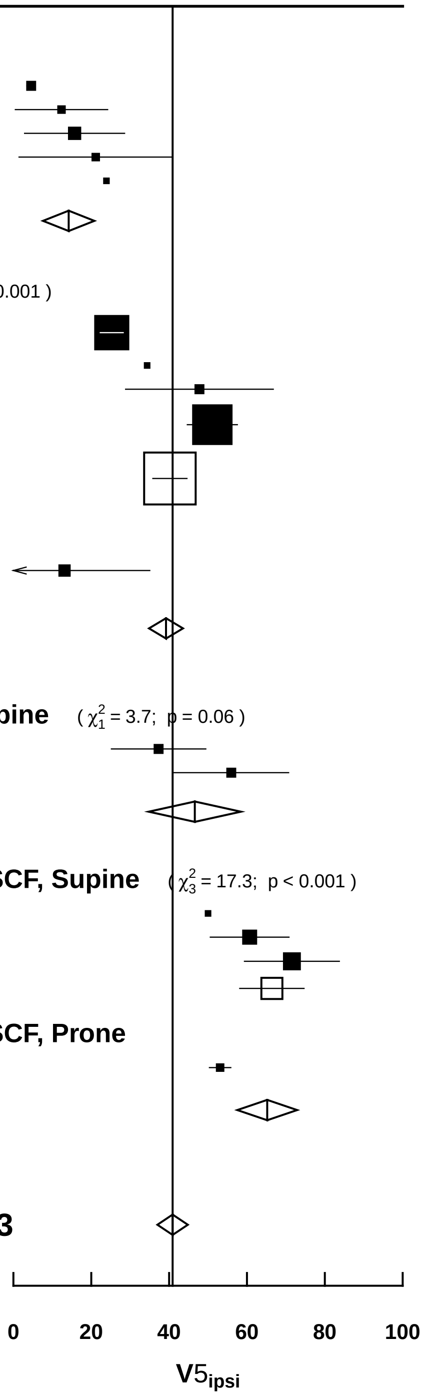

Figure E2. Percent volume of ipsilateral lung receiving 5 Gy or more ($V5_{ipsi}$) from breast cancer radiation therapy according to regions irradiated and technique used Regimens using breathing adaptation (e.g. deep inspiration breath hold) and studies of women with unfavourable anatomy were excluded.

∗ Average of ipsilateral $V5_{ipsi}$ for reported regimens.
† Range of ipsilateral $V5_{ipsi}$ for reported regimens.
‡ Static field IMRT and rotational IMRT are included jointly as IMRT.
§ Other techniques included two dynamic conformal arc therapy regimens and on unspecified 3D conformal regimen.
χ^2 and p are for heterogeneity.
Abbreviations: SE: standard error. CI: confidence interval. IMRT: intensity modulated radiotherapy. IMC: internal mammary chain. SCF: supraclavicular fossa.

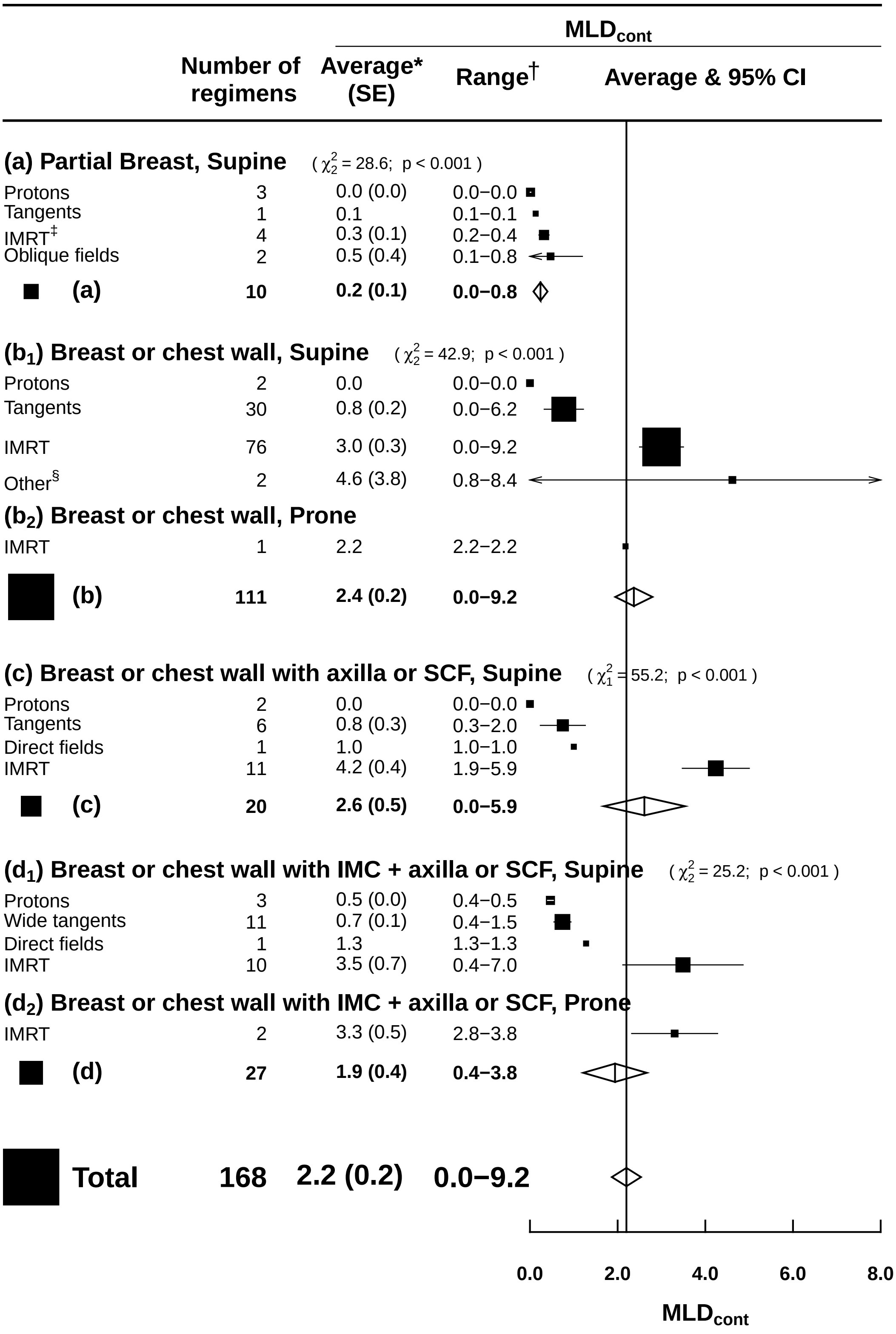

Figure E3. Mean contralateral lung doses (MLD$_{cont}$) from breast cancer radiotherapy according to region irradiated and technique used. Regimens using breathing adaptation (e.g. deep inspiration breath hold) and studies of women with unfavourable anatomy were excluded.

$*$ Average of MLD$_{cont}$ for reported regimens.
† Range of MLD$_{cont}$ for reported regimens.
‡ Static field IMRT and rotational IMRT are included jointly as IMRT.
§ Other techniques included two dynamic conformal arc therapy regimens and on unspecified 3D conformal regimen.
χ^2 and p are for heterogeneity.
Abbreviations: SE: standard error. CI: confidence interval. IMRT: intensity modulated radiotherapy. IMC: internal mammary chain. SCF: supraclavicular fossa.

	Number of regimens	MLD$_{whole}$ Average* (SE)	MLD$_{whole}$ Range†
**(a) Partial breast, Supine** ($\chi^2_2 = 17.3$; $p < 0.001$)			
Protons	3	0.4 (0.0)	0.3– 0.4
IMRT‡	4	1.1 (0.2)	0.7– 1.7
Tangents	1	1.6	1.6– 1.6
Oblique fields	3	2.8 (1.2)	1.4– 5.1
Brachytherapy	1	4.0	4.0– 4.0
**(a)**	**12**	**1.6 (0.4)**	**0.3– 5.1**
**(b_1) Breast or chest wall, Supine** ($\chi^2_3 = 195.0$; $p < 0.001$)			
Protons	3	1.4 (0.3)	0.9– 1.8
Oblique fields	1	3.3	3.3– 3.3
Tangents	42	4.6 (0.3)	2.1–10.7
IMRT	92	6.3 (0.3)	1.6–13.1
Other§	3	7.2 (1.0)	5.4– 8.7
**Subtotal**	**141**	**5.7 (0.2)**	**0.9–13.1**
**(b_2) Breast or chest wall, Prone** ($\chi^2_1 = 1.0$; $p = 0.3$)			
Tangents	3	0.8 (0.5)	0.2– 1.7
IMRT	3	2.4 (1.5)	0.8– 5.4
**Subtotal**	**6**	**1.6 (0.8)**	**0.2– 5.4**
**(b)**	**147**	**5.5 (0.2)**	**0.2–13.1**
**(c) Breast or chest wall with axilla or SCF, Supine** ($\chi^2_2 = 11.8$; $p = 0.003$)			
Protons	2	3.5 (1.3)	2.2– 4.8
Tangents	11	6.8 (0.3)	5.0– 8.3
IMRT	12	8.2 (0.6)	4.2–12.1
Direct fields	1	8.8	8.8– 8.8
**(c)**	**26**	**7.3 (0.4)**	**2.2–12.1**
**(d) Breast or chest wall with IMC + axilla or SCF, Supine** ($\chi^2_2 = 17.5$; $p < 0.001$)			
Protons	4	4.9 (0.9)	3.0– 7.0
IMRT	12	8.6 (0.4)	6.5–10.6
Wide tangents	16	9.3 (0.4)	5.3–11.9
Direct fields	1	12.1	12.1–12.1
**(d)**	**33**	**8.6 (0.4)**	**3.0–12.1**
**Total**	**218**	**6.0 (0.2)**	**0.2–13.1**

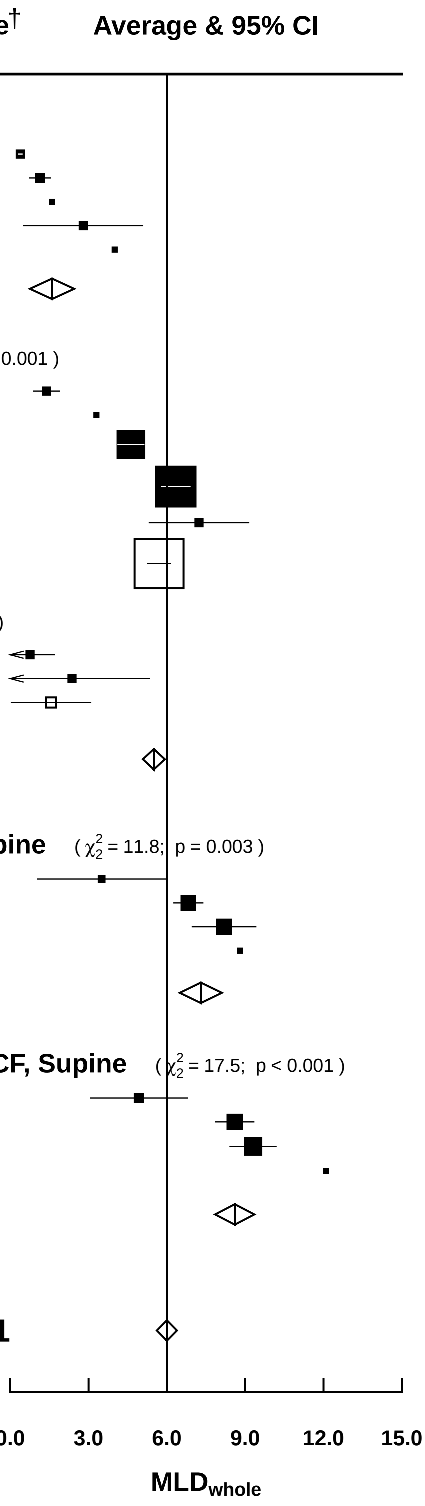

Figure E4. Mean whole lung doses (MLD$_{whole}$) from breast cancer radiotherapy according to region irradiated and technique used. Regimens using breathing adaptation (e.g. deep inspiration breath hold) and studies of women with unfavourable anatomy were excluded.

∗ Average of MLD$_{whole}$ for reported regimens.
† Range of MLD$_{whole}$ for reported regimens.
‡ Static field IMRT and rotational IMRT are included jointly as IMRT.
§ Other techniques included two dynamic conformal arc therapy regimens and on unspecified 3D conformal regimen.
χ^2 and p are for heterogeneity.
Abbreviations: SE: standard error. CI: confidence interval. IMRT: intensity modulated radiotherapy. IMC: internal mammary chain. SCF: supraclavicular fossa.
